# Structural Basis of Detection and Signaling of DNA Single-Strand Breaks by Human PARP-1

**DOI:** 10.1016/j.molcel.2015.10.032

**Published:** 2015-12-03

**Authors:** Sebastian Eustermann, Wing-Fung Wu, Marie-France Langelier, Ji-Chun Yang, Laura E. Easton, Amanda A. Riccio, John M. Pascal, David Neuhaus

**Affiliations:** 1 Medical Research Council, Laboratory of Molecular Biology, Francis Crick Avenue, Cambridge CB2 0QH, UK; 2 Department of Molecular Biology and Biochemistry, Sidney Kimmel Cancer Center, Thomas Jefferson University, 233 South 10th Street, Bluemle Life Sciences Building, Room 804, Philadelphia, PA 19107, USA

## Abstract

Poly(ADP-ribose)polymerase 1 (PARP-1) is a key eukaryotic stress sensor that responds in seconds to DNA single-strand breaks (SSBs), the most frequent genomic damage. A burst of poly(ADP-ribose) synthesis initiates DNA damage response, whereas PARP-1 inhibition kills BRCA-deficient tumor cells selectively, providing the first anti-cancer therapy based on synthetic lethality. However, the mechanism underlying PARP-1’s function remained obscure; inherent dynamics of SSBs and PARP-1’s multi-domain architecture hindered structural studies. Here we reveal the structural basis of SSB detection and how multi-domain folding underlies the allosteric switch that determines PARP-1’s signaling response. Two flexibly linked N-terminal zinc fingers recognize the extreme deformability of SSBs and drive co-operative, stepwise self-assembly of remaining PARP-1 domains to control the activity of the C-terminal catalytic domain. Automodifcation in *cis* explains the subsequent release of monomeric PARP-1 from DNA, allowing repair and replication to proceed. Our results provide a molecular framework for understanding PARP inhibitor action and, more generally, allosteric control of dynamic, multi-domain proteins.

## Highlights

• We identify the structural basis of DNA single-strand break detection by PARP-1
• An NMR/X-ray approach reveals how multi-domain allostery underlies activity switch
• The molecular model of PARP-1 assembly couples damage detection to PAR signaling
• Automodification in *cis* releases enzyme so DNA repair and replication can proceed

## Graphical Abstract

**Figure:**
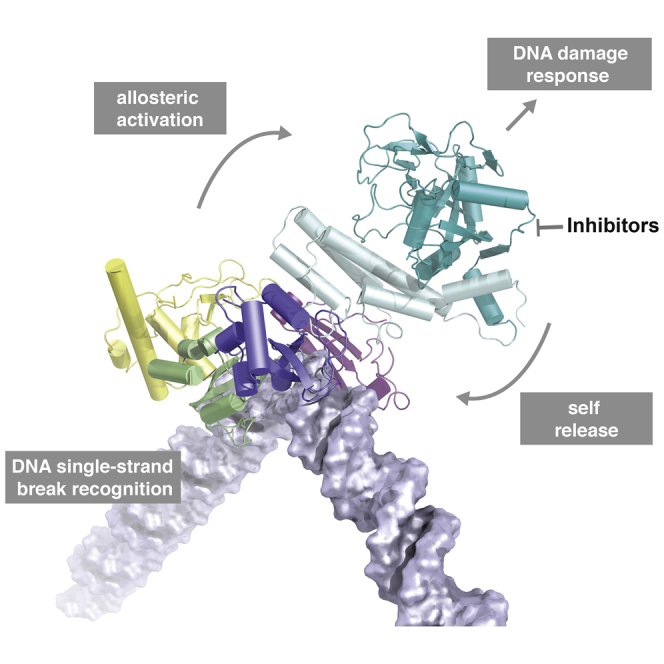

## Teaser

Eustermann et al. reveal the structural basis for recognition of DNA single-strand breaks by human PARP-1 and how this drives a multi-domain folding cascade to operate the allosteric switch initiating DNA damage signaling. The structural model of PARP-1 on a DNA break helps rationalize inhibitor action.

## Introduction

Poly(ADP-ribose)polymerase 1 (PARP-1) is a highly abundant chromatin-associated protein found in the nuclei of all higher eukaryotes. It is the founding member of a family of enzymes that modify a wide variety of target proteins with poly(ADP-ribose) (PAR), a highly negatively charged, branched-chain posttranslational modification derived from NAD^+^ (D’Amours et al., 1999, Krishnakumar and Kraus, 2010). Acute DNA damage-dependent activation of PARP-1, the major PAR-producing enzyme in eukaryotes, is one of the earliest cellular responses to genotoxic stress (Polo and Jackson, 2011) and links DNA damage response signaling and recruitment of DNA repair factors to a concerted modulation of chromatin structure (Durkacz et al., 1980, Schreiber et al., 2006, Chou et al., 2010). Excessive activation leads to cell death through NAD^+^ depletion (Fouquerel et al., 2014), whereas basal levels are required for other key functions such as transcriptional regulation (Kim et al., 2004, Schreiber et al., 2006). PARP-1 is central to the cellular stress response and has been implicated in a number of pathophysiological conditions (Luo and Kraus, 2012). Most prominently, it has emerged as an important target for cancer therapy (Bryant et al., 2005, Farmer et al., 2005). Numerous PARP inhibitors are in clinical trials, and, very recently, the first, olaparib (AstraZeneca), has been approved for treatment of advanced BRCA-dependent ovarian cancer (Sonnenblick et al., 2015). These inhibitors represent an entirely novel class of cancer therapeutics based on synthetic lethality. A collapse of genome integrity caused by the cumulative effects of PARP inhibition and defective homologous recombination (HR) repair kills BRCA-deficient tumor cells, whereas cells with intact HR repair are largely unaffected by PARP inhibitors under normal conditions. Similar effects involving other repair pathway deficiencies are now also coming to light (Murai et al., 2012, Mendes-Pereira et al., 2009), and possible causes of resistance to PARP inhibitors are being investigated (Lord and Ashworth, 2013). However, a major obstacle in understanding PARP-1’s role during genomic maintenance and the cause of the synthetic lethal effect of inhibitors (Helleday, 2011) has been that the molecular basis of its function remains poorly understood.

**Figure 1:**
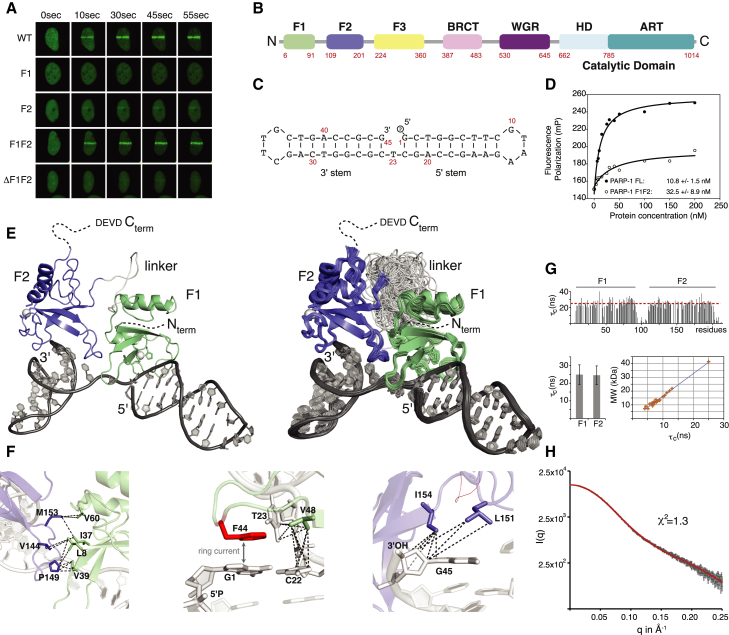
Structural Basis of SSB Recognition by PARP-1 (A) Live-cell imaging shows recruitment of GFP-labeled PARP-1 and PARP-1 fragments to sites of laser-induced DNA damage. WT, wild-type. (B) Domain structure of PARP-1. (C) Gapped dumbbell DNA ligand used in this work as a mimic of an SSB. (D) Fluorescence polarization experiments show that F1F2 binds the DNA ligand only about 3-fold less strongly than full-length PARP-1. FL, full-length. (E) NMR/X-ray hybrid structure of F1F2 bound to an SSB. Overall views of (left) the lowest-energy structure, and (right) the ensemble of all 78 accepted structures (see Experimental Procedures and Supplemental Experimental Procedures for details of structure determination). (F) Measured NOE contacts (dashed lines; Table S2) that define the F1-F2 interface (left), the hydrophobic interactions of F1 with the 5′ stem and T23 (center), and of F2 with the 3′ stem (right). The stacking interaction between F44 (red) and G1 was inferred from strong CSPs caused by their aromatic ring currents. (G) Effective τ_c_ values obtained from ^15^N relaxation (TRACT) experiments with the F1F2 complex are consistent with a 40-kDa species (1:1 stoichiometry) in which both fingers bind simultaneously and the linker remains flexible. (H) The experimental SAXS profile of the PARP-1 F1F2 dumbbell-DNA complex (3 mg.ml^−1^) agrees with the back-calculated SAXS profile averaged over the ensemble shown in (E). See also Figures S1, S2, S3, and S4 and Tables S1 and S2.

DNA single-strand breaks (SSBs) are by far the most frequent form of DNA damage, resulting both directly from oxidative damage and as intermediates in other DNA repair pathways (Caldecott, 2008). Although repair of such chemically diverse DNA lesions has been much studied, the detailed structural mechanism by which they are efficiently detected and signalled to the DNA repair machinery has remained elusive. PARP-1 has long been known as a crucial first-line sensor of SSBs (de Murcia and Ménissier de Murcia, 1994, Satoh and Lindahl, 1992), and functional studies have consistently highlighted its physiological importance as well as its central role for PARP inhibitor action (Helleday, 2011, Bryant et al., 2005, Caldecott, 2014, Murai et al., 2012). Despite the diversity of SSBs, PARP-1 recruitment to sites of genomic damage and PAR-mediated signaling must be both rapid and robust while, at the same time, maintaining the dynamic range and tight control required for PARP-1’s cellular function. PARP-1 comprises six domains connected by flexible linkers (Figure 1B), and, in the free state, these domains are independent, behaving like “beads on a string” (Lilyestrom et al., 2010). Recent crystal structures of different combinations of domains from PARP-1 bound to the ends of short DNA duplexes as mimics of DNA double-strand breaks (DSBs) provided important insights by showing the existence of individual domain-domain interactions required for activation (Langelier et al., 2012, Ali et al., 2012). However, these static views did not establish the mechanism by which the observed interactions arise or show whether they would form a rapid yet adjustable switch for PARP-1 activation. Mutually exclusive DNA-binding modes were observed, leading to substantially different proposals regarding damage recognition and activation. As for many key signaling proteins, PARP-1’s highly dynamic and modular architecture has hindered structural studies. However, it is likely that these dynamics underlie the way PARP-1 achieves its complex roles in genome maintenance. Recent studies of other systems have shown the importance of allosteric and cooperative effects within highly dynamic multi-domain proteins in defining cellular responses, although the principles underlying such effects are only now emerging (Chao et al., 2011, Mackereth et al., 2011).

Here we used an integrated nuclear magnetic resonance (NMR)/X-ray approach to establish the molecular mechanism by which PARP-1 senses SSBs and becomes allosterically activated. Because key interactions are detected in solution using NMR, inherent flexibility no longer poses the obstacle that it does for crystallography, allowing us to interrogate complexes representing successive assembly states of the system and, thereby, build up the stepwise, co-operative, multi-domain folding pathway that underlies the operation of PARP-1’s DNA damage-dependent activity switch during genomic maintenance.

## Results and Discussion

### Capturing Recognition of DNA Single-Strand Breaks In Vitro

**Figure 2:**
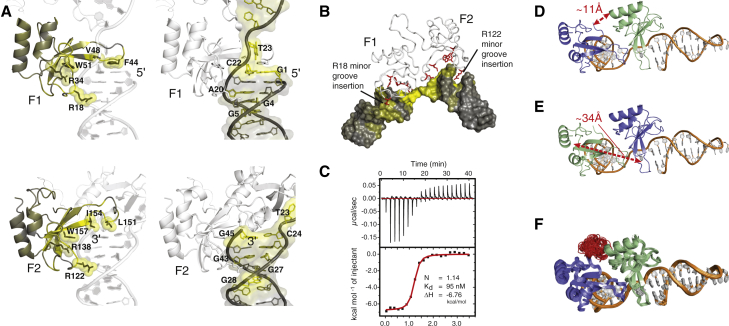
Interactions of F1 and F2 on Either Side of the SSB (A) CSPs on DNA binding mapped to the structure for protein amide groups (left) and DNA 1′-CH groups (right), for the F1-5′ DNA stem interface (top), and for the F2-3′ DNA stem interface (bottom). See Figure S2 for details and definitions of gray to yellow color ramps. Large CSPs occur for the key interacting protein residues labeled. For the DNA, CSPs match the 7-base pair footprints of the fingers on either side of the break (de Murcia and Ménissier de Murcia, 1994), with the largest effects at the exposed stem ends (C22-G1 and C24-G45) and sites of arginine insertions into the minor groove (Arg122 of F2 near C27, G28, and G43 in the 3′ stem and Arg18 of F1 near G4, G5, and A20 in the 5′ stem). (B) CSPs measured for the DNA on F1F2 binding, illustrating the largest perturbations that occur at the DNA damage site. Protein side chains interacting with the DNA backbone are shown in red. (C) Isothermal calorimetry shows that high-affinity binding of PARP-1 F1F2 to the gapped DNA dumbbell ligand occurs with 1:1 stoichiometry and is fully saturated at higher protein:DNA ratios (note that the apparent K_d_ is unreliable under these stoichiometric conditions). (D and E) Schematics showing how reversing the directionality of the protein on the SSB would affect the F1-F2 linker (which was shown to be flexible by NMR; Figure 1G). If F1 bound the 3′ stem and F2 the 5′, then the distance spanned by the linker would increase from ∼11 Å in the actual complex (D) to ∼34 Å in the hypothetical reversed complex (E). (F) Further calculations show that the observed arrangement of F1 and F2 is also consistent with the artificially shortened linker (Δ94–102). Ali et al. (2012) have reported that PARP-1 Δ94–102 localizes to laser-induced DNA damage in a very similar manner as wild-type protein in vivo.

To understand the molecular basis of the PARP-1 response to genomic damage, we first sought to dissect how DNA damage is recognized. While crystallographic studies so far have employed mimics of DSBs, we set out to determine the structural basis of SSB recognition using solution-state NMR spectroscopy. We used a minimal protein construct comprising PARP-1’s flexibly linked N-terminal zinc fingers F1F2 (Figure 1B), corresponding to the naturally occurring caspase-3 cleavage product of PARP-1. Our live-cell imaging experiments show that, similar to full-length PARP-1, F1F2 localizes within seconds to sites of laser-induced DNA damage, whereas isolated F1 or F2 domains showed no recruitment or only residual levels, respectively (Figure 1A). This shows that the two fingers must act co-operatively, corroborating previous functional studies (de Murcia et al., 1994, Molinete et al., 1993) and the mutational analysis by Ali et al. (2012). To recapitulate these features of PARP-1 recruitment in vitro, we employed our previously established model system for DNA single-strand breaks (Figure 1C; Eustermann et al., 2011). Given the micromolar concentration of PARP-1 inside eukaryotic nuclei (D’Amours et al., 1999), the measured nanomolar affinities for this ligand are in agreement with PARP-1’s function as a bona fide DNA damage sensor, and F1F2 binds SSBs only slightly less strongly than does full-length PARP-1 (Figure 1D). The interaction is sequence-independent (Figure S1), DNA-damage specific, and co-operative. Binding of isolated F2 to the DNA dumbbell is approximately 10-fold weaker than for F1F2, and F1 is much weaker still (Eustermann et al., 2011). Notably, isothermal calorimetry (ITC) (Figure 2C) and transverse relaxation-optimized spectroscopy (TROSY) for rotational correlation times (TRACT) NMR (Figure 1G) experiments confirmed that SSB recognition occurs as a monomer with a 1:1 stoichiometry (Lilyestrom et al., 2010, Eustermann et al., 2011) and showed that both fingers are bound simultaneously, consistent with their cooperative role in vitro and in vivo. Previously reported models based on DNA duplexes as mimics of DSBs have either suggested that cooperative action of F1 and F2 results in DNA damage-induced dimerization (Ali et al., 2012) or did not include F2 but have suggested monomeric DSB activation (Langelier et al., 2012). Based on our findings, we concluded that a complex of F1F2 with dumbbell DNA represents a minimal structural unit required to capture the first stage of PARP-1’s interaction with SSBs.

The challenges of determining a structure of this size by NMR were overcome mainly by combining TROSY-based experiments (Fernández and Wider, 2003) with a targeted isotope-labeling strategy and, where necessary, using ligation via the enzyme sortase A (Kobashigawa et al., 2009) to produce chains with different isotopic labeling patterns in different domains to reduce spectral overlap and facilitate interpretation. A similar approach was used to make signal assignments for the larger complexes described later in this paper. This approach allowed us to obtain extensive protein and DNA assignments and measure key structural information such as residual dipolar couplings (Figures S4a–S4c), protein backbone dynamics (Figure 1G; Figure S5f), and assigned nuclear Overhauser effect (NOE) contacts within domains and at the domain-domain and protein-DNA interaction interfaces (Figure 1F; Figures S2d–S2k). Intriguingly, our NMR data identified local contacts of F1 and F2 with DNA that were analogous to those observed in previous crystal structures of the isolated fingers on DNA blunt ends (Langelier et al., 2011) despite occurring in a structurally different context. Building on related approaches for characterizing flexible multi-domain proteins and their complexes (Mackereth et al., 2011, Göbl et al., 2014), we were therefore able to incorporate direct knowledge of these crystal structures while our NMR data provided the key information to determine the overall structure as well as other aspects of the system where flexibility poses problems for crystallography (Experimental Procedures; Supplemental Experimental Procedures; Table 1; Table S2).

### Structural Basis of DNA Single-Strand Break Recognition

The most striking feature of the determined structure of F1F2 bound to the gapped DNA dumbbell (Figure 1E) is the way in which binding of the two flexibly linked finger domains on either side of the break opens up the structure of the DNA. It is immediately clear that undamaged double-helical DNA could never adopt such a conformation. The fingers, and, consequently, also the DNA stems to which they are bound, become mutually oriented through acquisition of a small but defined DNA-dependent, hydrophobic F1-F2 interface (average area, 359 ± 35 Å^2^) (identified by NOE contacts; Figure 1F; Figures S2e–S2g), and they adopt a single directionality on the DNA with F2 on the 3′ stem and F1 on the 5′ stem (as evidenced by the data in Figures 1F, 2A, and 2B), whereas the linker remains flexible in the complex (Figure 1G). The average bend angle of the DNA around the break (approximately 107° ± 1° across the ensemble) matches closely early positive-stain electron microscopy measurements (102° ± 44°) (Le Cam et al., 1994) and was cross-validated further by our measured small-angle X-ray scattering (SAXS) data that showed excellent agreement with the back-calculated scattering curve of the structural ensemble (Figure 1H). Not only is a severe kink induced at the SSB, but, crucially, the two stems are also twisted apart in such a way that the faces of all four flanking bases are exposed to allow interactions with the protein. Intriguingly, the two fingers each show a highly similar mode of interaction with their respective stems. The observed NOE and chemical shift perturbation (CSP) data clearly show that local interactions of F1 and F2 with DNA bases and adjacent minor grooves show the same pattern on both sides of the break (Figures 1F, 2A, and 2B Table S2), closely paralleling those observed in crystal structures of isolated fingers, each bound across the 3′ terminus of a DNA stem (Langelier et al., 2011). However, although such observations from the crystal structures might have suggested that binding of the fingers was mutually exclusive, our solution structure shows how the flexibility of an SSB accommodates the simultaneous binding of F2 and F1 despite the asymmetric nature of the 5′ and 3′ stems (Figures 1E–1G). Steric clashes between the fingers are avoided as the continuous DNA strand linking the stems adopts a conformation very far from B-form. In effect, the F1 binding site is cryptic, becoming exposed only as a result of the severe distortions of the linking strand imposed by binding of F2 to the other site. The resulting overall protein-DNA interface is large enough to provide PARP-1 with sufficient affinity and specificity for F1F2 binding, explaining the cooperative role of the fingers in vivo (Figure 1A). Notably, neither finger makes direct contacts with the DNA termini (Figure 1F), consistent with the role of PARP-1 as a first-line sensor of a wide variety of chemically diverse SSB types. This explains our previous observation that SSB detection by F1F2 is essentially independent of terminal modifications such as 3′ phosphorylation (a common result of oxidative DNA backbone damage) (Eustermann et al., 2011).

**Figure 3:**
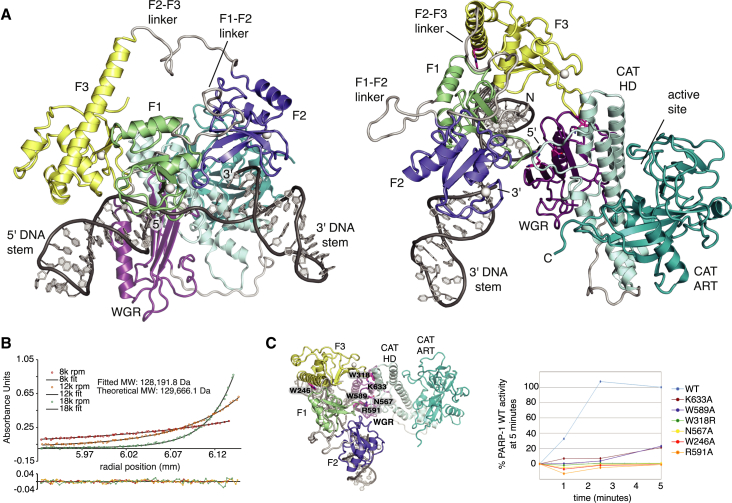
Structural Model of Full-Length PARP-1 Bound to a DNA Single-Strand Break (A) Superposition of the hybrid structure of F1F2 bound to a DNA dumbbell and the previous crystal structure of F1, F3, and WGR-CAT bound to a DNA duplex (PDB: 4DQY) led directly to the domain arrangement shown (see also Figures S5g and S5h, Experimental Procedures, and Supplemental Experimental Procedures). The F1-F2 and F2-F3 linkers are flexible (see the NMR ^15^N relaxation data in Figure S5f), whereas the BRCT domain (not required for activity) and its linkers are omitted but may also adopt a wide variety of locations. The structure of PARP-1 on an SSB was corroborated using biophysical and mutational analysis (see B and C), NMR spectroscopy that elucidated its dynamic assembly process (Figure 4), and HXMS experiments (Dawicki-McKenna et al., 2015). (B) Analytical ultracentrifugation shows that full-length PARP-1 binds to the gapped DNA dumbbell with a 1:1 stoichiometry. MW, molecular weight. (C) Catalytic activities of wild-type PARP-1 and the designated mutants were assessed using a colorimetric activity assay (Supplemental Experimental Procedures) using the dumbbell gap DNA as the activating ligand.

A key finding of our study is that F1F2 binds in only one direction on an SSB, and the direction it selects (F2 on the 3′ stem, F1 on the 5′) is the only one that triggers activation (Figure 3). Given the dynamic nature of the system, the similarity of the fingers and the absence of direct contacts with the termini, it is perhaps surprising that PARP-1 shows this directional selectivity on SSBs. Our study suggests three possible contributions. First, the F1-F2 interface we see can only form when the fingers bind in the observed sense. Second, the linker path in a hypothetical reversed complex would be much longer (e.g., swapping F1 and F2 would increase the distance A91Cα-T109Cα from ∼11 to ∼34 Å), suggesting that the linker could act as an “entropic spring,” favoring the shorter path (Figures 2D and 2E). Third, if F2, the finger with the higher affinity, wins the competition to bind first, then this will presumably direct it to the 3′ stem because DNA distortions required to reveal this site are much smaller than those required to reveal the “cryptic” second site on the 5′ stem.

Taken together, our results identify a consensus that many DNA structures that activate PARP-1 have in common, comprising two flexibly linked DNA stems with exposed bases at the ends, independent of 3′ or 5′ modifications. When F2 has initiated recognition by binding at the 3′ stem, subsequent scanning for the second site by the flexibly linked F1 domain resembles a “fly-casting” mechanism (Shoemaker et al., 2000), elegantly explaining how PARP-1 efficiently recognizes DNA single-strand breaks with different gap lengths. Interestingly, such structures may also exist at stalled replication forks, which also efficiently activate PARP-1, depending on the length of single-stranded region they contain (Bryant et al., 2009). Indeed, one may even speculate that PARP-1 could recognize DSBs by an analogous mechanism, provided the two stems at the DNA break are held in sufficiently close proximity either directly by PARP-1 binding or by DSB sensors that are known to tether DSB ends (e.g., the Mre11-Rad50-Nbs1 [MRN] complex; Moreno-Herrero et al., 2005). Under other circumstances, where a DNA damage site is more rigid, binding and activation become uncoupled. Others have shown, using pull-downs from cell lysate, that PARP-1 is a prominent sensor of abasic sites but that activation only occurs after apurinic/apyrimidinic (AP) endonuclease transforms such lesions into SSBs (Khodyreva et al., 2010). Detecting “DNA deformability” at damage sites is a mechanism seen in other DNA repair systems (Lamers et al., 2000, Min and Pavletich, 2007). However, unlike other repair factors, PARP-1 uses two flexibly linked recognition modules, and our data establish a distinct mechanism for versatile yet specific DNA damage recognition, providing a unified explanation of PARP-1’s involvement in diverse DNA repair pathways.

### DNA Damage Recognition Drives PARP-1’s Allosteric Activity Switch

Having shown how PARP-1 recognizes SSBs, we sought next to understand how this leads to allosteric activation. Combination of our structure of F1F2 on an SSB with the previous crystal structures of F1, F3, and WGR-CAT on a DSB (Langelier et al., 2012) leads directly to a structural model of full-length PARP-1 assembled on an SSB (Figure 3A). Perhaps surprisingly, there are no steric clashes, and all domains can be linked in a single polypeptide chain, fully consistent with our analytical ultra-centrifugation (AUC) data for full-length SSB-bound PARP-1 (Figure 3B). Previous crystallographic analysis of DSB recognition by F1F2 led to the suggestion that PARP-1 is activated by DNA-induced dimerization (Ali et al., 2012), a mechanism found in many DNA binding proteins and suggested previously for PARP-1. However, the monomeric arrangement of the two fingers on an SSB that we observe in solution differs substantially. Our data emphasize the critical importance of directional binding of F1 and F2 because only when F1 is positioned on the cryptic site on the 5′ stem can F1 subsequently interact with the F3 and WGR domains (Figure 3A). This is highly relevant because Langelier et al. (2012) observed a ternary interaction of the F3, WGR, and CAT domains that distorts the regulatory HD subdomain, thereby destabilizing CAT and priming it for productive catalysis. A separate study by Pascal, Black, and colleagues using hydrogen exchange mass spectrometry (HXMS) has now shown that the HD subdomain can undergo local unfolding to release an unanticipated inhibitory effect (Dawicki-McKenna et al., 2015 [this issue of *Molecular Cell*]). Intriguingly, there are no direct contacts in our model between the CAT domain and F1, F2, or the DNA, showing that communication must occur through F3 and WGR. Our mutational analysis shows that interactions of the latter domains within monomeric PARP-1 are essential for activation by SSBs (Figure 3C). Taking this together, we hypothesized that the manner in which the domains of PARP-1 fold up onto an SSB must provide the free energy responsible for destabilizing CAT and that it is this process that underlies the rapid and robust, yet tunable, operation of the of the switch controlling PARP-1 activity.

**Figure 4:**
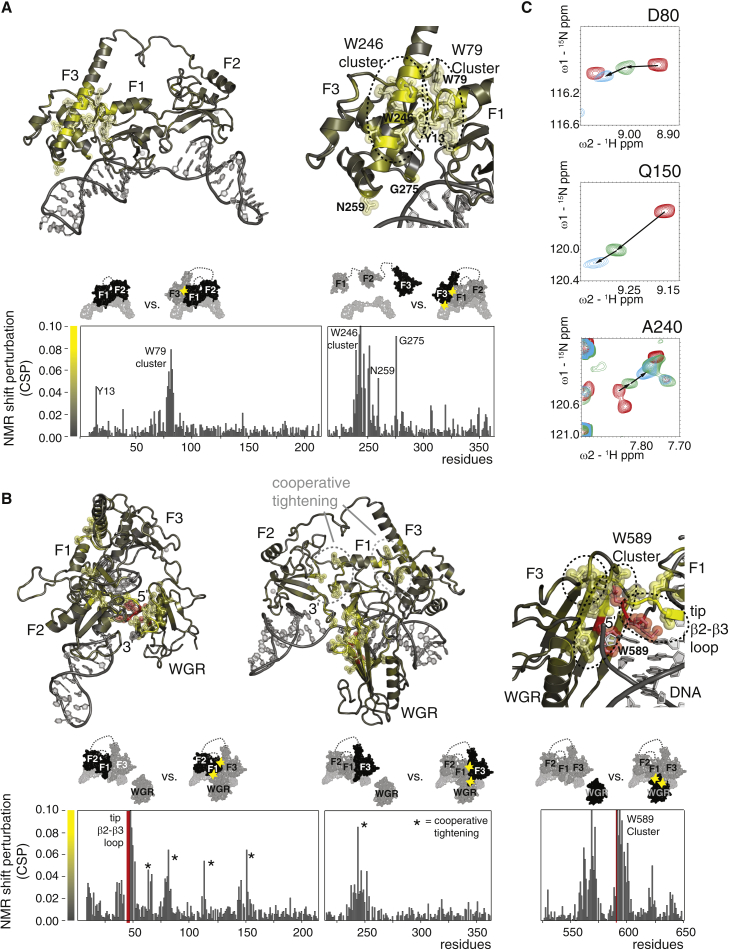
NMR Analysis Uncovers the Multi-Domain Folding Pathway of PARP-1 Domains on an SSB that Underlies DNA-Dependent Switching of Its Activity (A and B) The 56-kDa F1F2F3-DNA and 71-kDa F1F2F3-WGR-DNA complexes represent intermediate steps toward full assembly. SSB recognition by F1F2 triggers interactions that place F3 (A) and WGR (B) in the correct spatial orientation for the interaction with CAT to trigger its activation. These interactions are evidenced by the amide group CSPs shown in the histograms and mapped on the structures (same co-ordinates as in Figure 3) from gray (CSP = 0) to yellow (CSP = 0.08; CSP > 0.08 is shown in yellow, and side chains are shown for CSP > 0.04). Four residues (red) close to the DNA 5′ terminus became undetectable because of line broadening during WGR titrations. CSPs were measured using sortase-ligated samples to reduce spectral complexity (see the schematics above the histograms; ^15^N-labeled domains are shown in dark gray, and interaction sites are highlighted in yellow). (C) In addition to direct interactions, CSPs also show co-operative strengthening throughout the F1F2F3-WGR-DNA complex; addition of WGR reinforces CSPs seen at the F1-F2 and F1-F3 interfaces. Representative examples are shown for each interface (F1-F2, Q150; F1-F3, D80 and A240), superposing spectra of F1F2F3 (red), F1F2F3-DNA (green), and F1F2F3-WGR-DNA (cyan). See also Figure S5.

Because static views of fully assembled PARP-1 complexes do not reveal the nature of such events, we turned again to NMR spectroscopy to follow the DNA-induced folding of PARP-1 by identifying and characterizing possible intermediate steps. Comparative analysis of DNA-dependent chemical shift perturbations (CSPs) clearly revealed that SSB binding by F1F2 triggers interactions of F3 with both F1 and the 5′ stem of the DNA (Figure 4). However, these interactions are delicately poised. They occur only when F3 is covalently linked to F1F2 (data not shown) and require correct spatial pre-organization of the contact surfaces on F1 and the DNA so that both interfaces to F3 can form simultaneously. ^15^N relaxation NMR experiments show that the F1-F2 and F2-F3 linkers are both highly flexible in the 56-kDa complex (Figure S5f) and also show that the F3 domain is not as rigidly associated with the rest of the complex as F1 or F2. Measured ^15^N R_1ρ_ rates for F3 are slower than those for F1 or F2, suggesting a looser, more transient interaction. In fact, a single point mutation of F3 at the F1 interaction surface can completely release F3 from F1 and the DNA. All of the CSPs characteristic of the F3-F1 and F3-DNA interactions are missing for F1F2F3 W246A (Figure S5e), and, importantly, the same mutation abolishes activation of full-length PARP-1 (Figure 3C). The arrangement of F1F2 on the SSB is also responsible for directing assembly of the WGR domain. The tip of the F1 base-stacking loop and the 5′ DNA terminus together create a composite surface for WGR interaction, and these interactions are strong enough to observe as CSPs in NMR titrations of the WGR domain with pre-assembled F1F2F3-SSB, resulting in a 71-kDa complex (however, the small difference in affinities of full-length PARP-1 and F1F2 for the SSB suggest that WGR binding must still be relatively weak; Figure 1D). Not only do these experiments show significant CSPs at points of direct contact that are required for activity (Figure 4B), but, also, many of the SSB-dependent CSPs already observed for the other interfaces of F1F2F3 become more pronounced upon addition of WGR, showing that WGR binding causes co-operative strengthening of inter-domain interactions throughout the complex (Figure 4C).

**Figure 5:**
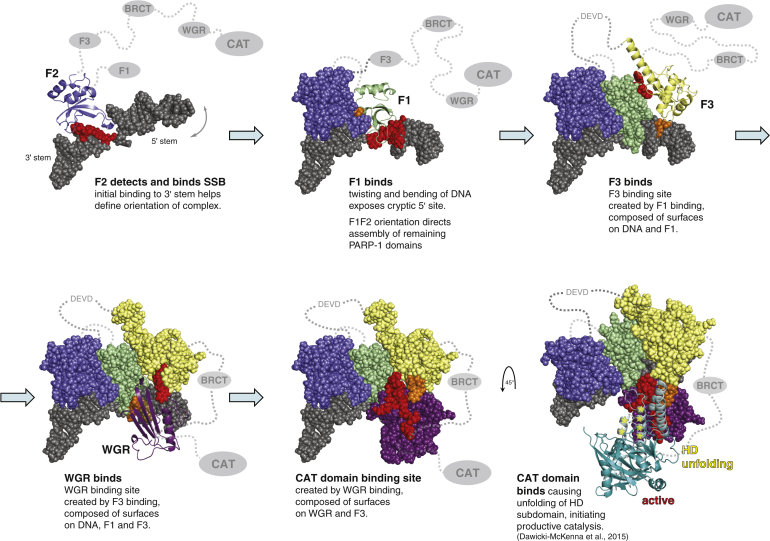
Allosteric Activation Mechanism of PARP-1 by DNA Single-Strand Breaks In the absence of DNA, PARP-1 domains behave independently, connected by disordered linkers. SSB recognition by F1 and F2 drives multidomain folding, which provides a cooperative switch for activation of the C-terminal catalytic domain (see main text); F3 and WGR are thereby positioned in the correct spatial orientation to trigger their ternary interaction with the catalytic domain. This relieves autoinhibition of the enzyme by causing local unfolding of the HD subdomain (indicated by stars in the figure), which is the subject of the accompanying paper by Dawicki-McKenna (2015). Interfacial residues on different components are colored red and orange. DEVD indicates the caspase cleavage site between F2 and F3. In this figure, we show the pathway as a sequence of discrete steps, but, in reality, they are probably not fully separated. For instance, although F2 likely initiates binding, F1 also co-operates in high-affinity DNA damage recognition. Similarly, although we show F3 binding ahead of WGR, in principle, these events could occur in either order or both. Nevertheless, our data show that elements of the pathway represent intermediate steps because they can occur in isolation. For instance, F1F2 binds an SSB and achieves directional selectivity on its own, and interactions of F3 and WGR occur with the F1F2-DNA complex in the absence of the CAT domain. BRCT, BRCA-1 C terminus.

By showing how high-affinity SSB detection by F1F2 provides the driving force to bring together F3 and WGR in the correct spatial orientation in the absence of CAT, we reveal intermediate steps on a multi-domain folding pathway that explain why CAT binding and consequent destabilization take place, initiating productive PAR catalysis. Association of each domain is required to create the binding platform for the next. Because individual interactions subsequent to DNA damage recognition are weak and are built from small parts on separate components, these must be pre-organized by previous assembly steps to form an organized whole (Figure 5). Our data provide direct insights into this process, showing how each step reduces the conformational space of the system, ultimately reducing the entropic cost of the ternary F3, WGR, and CAT interaction and providing the free energy for CAT destablization. In contrast, in the absence of DNA, pre-organization among the domains is missing, the resulting partial inter-domain interfaces are not individually strong enough to form, except perhaps transiently, and, consequently, PARP-1 remains inactive. The co-operative nature of this DNA-induced self-assembly process ensures that PARP-1 is robustly switched between inactive and active states. The process has parallels to protein folding and is an example of what Hunter and Anderson (2009) termed “chelating co-operativity.” Furthermore, by identifying the underlying intermediate steps and their dynamics, we provide a framework for understanding the selective regulation of PARP-1. For instance, caspase-3 inactivation of PARP-1, a hallmark of apoptosis (Lazebnik et al., 1994), results from abrogation of the finely balanced interactions of F3 caused by cleavage of the F2-F3 linker. Just as in the case of the W246A mutation, this leads to breakdown of the co-operative pathway of allosteric communication. Interestingly, PARP-1 has been identified recently as one of the most heavily acetylated cellular proteins in response to UV irradiation (Elia et al., 2015), and modification sites map to interaction surfaces, e.g., of F1, F2, and WGR. We propose that the large number and variety of post-translational modifications and interaction partners of PARP-1 (Luo and Kraus, 2012) may allow fine-tuning of its activity in a pathway-specific manner.

### PARP-1 Automodification Occurs In *cis*

The structural model of full-length PARP-1 represents the enzyme in a state primed for productive PAR catalysis. It remains an open question what specificity may exist in substrate selection. Proteomics studies have so far not yielded a defined consensus modification site (Zhang et al., 2013), and PARP-1 modifies a wide variety of proteins. We suggest that specificity could depend mainly on substrate recruitment by parts of PARP-1 other than its catalytic domain. The role of these would simply be to bring the target into proximity for modification, as suggested similarly for PAR-ylation by TNKS2 (Guettler et al., 2011). The major target of PAR-ylation is PARP-1 itself, modification of which serves as an important signal for the DNA damage response and releases PARP-1 from DNA (Satoh and Lindahl, 1992, D’Amours et al., 1999, Murai et al., 2012). The monomeric mechanism of SSB-induced activation presented above predicts that such automodification should occur in *cis*, with the same PARP-1 molecule that detects DNA damage also serving as the substrate for automodification.

**Figure 6:**
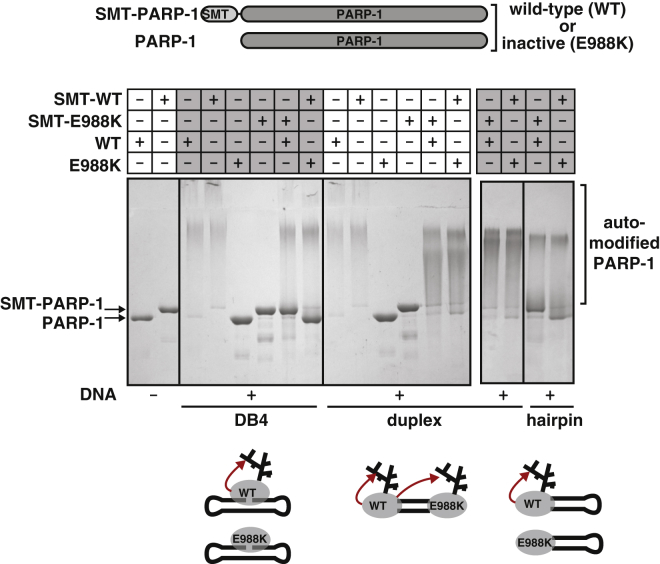
Automodification of PARP-1 on SSBs Occurs In *cis* Active WT or inactive mutant (E988K) versions were made for both normal and N-terminal SUMO-tagged (SMT) variants of PARP-1 (distinguishable on SDS-PAGE). On mixing active and inactive versions with different tags in the presence of an SSB mimic (DB4; Supplemental Experimental Procedures), only the active WT is automodified (slower-migrating smeared band), indicating selective in *cis* modification of this type of DNA damage. In contrast, both active and inactive PARP-1 molecules are modified in the presence of a DNA duplex, which allows binding on both DNA ends. Blocking one end of the duplex with a hairpin restores selective in *cis* modification.

However, to date, only automodification in *trans* has been reported, based mainly on observed bimolecular reaction kinetics when active calf thymus DNA containing a highly diverse range of DNA ligands was used for activation (Mendoza-Alvarez and Alvarez-Gonzalez, 1993). Indeed, the supposed in *trans* nature of automodification has been invoked widely to support the case for involvement of PARP-1 dimers in catalysis (Pion et al., 2005, Ali et al., 2012). Our in vitro activity assays shown in Figure 6 resolve this paradox. Only when two DNA binding sites are closely adjacent (e.g., a DNA duplex) does automodification occur in *trans*, whereas, on SSB (dumbbell) and DSB (single hairpin) mimics, PARP-1 automodification occurs almost exclusively in *cis*. Selective in *cis* modification likely presents specific regions of PARP-1 for automodification. Indeed, our structural model of SSB-bound full-length PARP-1 together with HXMS data (Dawicki-McKenna et al., 2015) shows that the BRCT-WGR linker, which is known to be automodified, remains flexible and is able to reach the active site of PARP-1. Future proteomic analyses that focus on DNA damage models like those presented in Figure 6, as well as further mechanistic studies of PARP-1’s dynamic nature in particular of the catalytic domain, will likely improve our understanding of how preferential automodification sites regulate biological functions of PARP-1 and other chromatin-associated targets. In *cis* automodification of the activated PARP-1 monomer explains elegantly how the enzyme can rapidly release itself from DNA damage, limiting NAD^+^ consumption and allowing DNA repair to proceed.

### Conclusions

In this work, we show how the dynamic response of PARP-1’s multi-domain structure to DNA single-strand breaks provides a molecular basis for understanding its central role as a cellular sensor of genotoxic stress. By using NMR spectroscopy in conjunction with other biophysical and functional techniques, we identify and interrogate successive states on a multi-domain folding pathway of PARP-1 in solution, demonstrating both the structural basis of SSB recognition and the allosteric mechanism through which recruitment to sites of genomic damage is intimately coupled to regulation of PARP-1’s catalytic activity for PAR-mediated signaling (summarized in Figure 5).

Many DNA-binding factors recognize their cognate target sites through co-operative homo- and hetero-oligomerization, thereby preventing unwanted activity (e.g., in the absence of interaction partners or at unspecific DNA binding sites). Well known examples include the nuclear hormone receptors (recently reviewed in Helsen et al., 2012). Although DNA-damage-induced dimerization has been proposed previously for PARP-1 (Mendoza-Alvarez and Alvarez-Gonzalez, 1993, Pion et al., 2003, Ali et al., 2012), our data show how the enzyme detects DNA damage as a monomer through co-operative action of its two flexibly linked N-terminal zinc fingers. Based on our findings, we propose a mechanism in which F1 is positioned through binding of F2 via a fly-casting process (Shoemaker et al., 2000). The dynamic features we identify explain how PARP-1 can serve as versatile yet specific first-line sensor of SSBs and related DNA structures in eukaryotes, efficiently recognizing the many chemically diverse lesions constantly arising from oxidative DNA damage or as intermediates during DNA repair.

Rather than using DNA-binding to bring together separate protein molecules to initiate allosteric activation, PARP-1 instead uses DNA-binding to bring together protein domains within the same molecule. The unique directionality of F1F2 binding establishes a platform for dynamic self-assembly of remaining PARP-1 domains onto the complex. Our data explain how this co-operative, multi-domain folding process acts as a rapid and robust switch for activation, effectively channeling energy from high-affinity DNA binding for activation of PARP-1’s catalytic domain, where it triggers productive PAR synthesis via local unfolding of an inhibitory HD subdomain, as observed by Dawicki-McKenna et al. (2015). Overall, this allosteric mechanism ensures that the resulting burst of PAR-mediated signaling and modulation of chromatin structure occurs only at sites of genomic lesions, while automodification in *cis* efficiently removes the monomeric enzyme so that DNA repair and replication can proceed.

Originally, it was suggested that delayed SSB repair (SSBR) caused by PARP-1 inhibition leads to collapsed replication forks and an increase of toxic DSBs (Bryant et al., 2005, Farmer et al., 2005), whereas, more recently, it has been suggested that PARP-1 is also a first-line sensor of stalled forks, providing a bypass for a defective homologous recombination (HR)-mediated replication restart (Bryant et al., 2009). Intriguingly, our data explain how PARP-1 can achieve both of these roles by using the same recognition and activation mechanism, implying that PARP inhibitors may target the same state of the enzyme in more than one pathway. Furthermore, stalling of PARP-1 on DNA lesions has very recently been proposed to form a crucial contribution to synthetic lethality because it may block repair and replication (Murai et al., 2012, Caldecott, 2014, Helleday, 2011, Shen et al., 2015). The SSB-bound conformation of PARP-1 presented here is most likely analogous to such a trapped state of the enzyme, in which high-affinity binding and domain assembly have occurred but subsequent release is prevented by inhibition of PAR automodification. Significantly, recent results have shown that the ability of clinically used PARP inhibitors to kill tumor cells does not correlate with their ability to prevent PAR production but, rather, with their ability to strengthen DNA binding of the inhibited state, which, in turn, must involve allosteric communication between the inhibitor binding site and DNA binding domains of PARP-1 (Murai et al., 2012, Marchand et al., 2014, Mansoorabadi et al., 2014). A detailed knowledge of the cooperative mechanism of PARP-1 assembly on DNA damage, such as provided here, will be essential to understand these effects and may play a key role in the future design of improved inhibitors.

To fulfill its functions, PARP-1 requires a combination of rapid response to an initial stimulus coupled with tight spatio-temporal control of catalytic activity, while also allowing more subtle degrees of regulation. The mechanism of SSB detection and allosteric activation we describe here is ideally suited to meet these demands. Ligand-induced multi-domain folding of domains within a single polypeptide chain represents an extremely efficient way to communicate a signal while maintaining, because of its dynamics, the potential for versatility and modulation so far more often associated with more complex but slower protein interaction networks. It seems likely that other signaling systems may have evolved similar solutions. Allosteric control of dynamic multi-domain proteins has emerged as one of the key molecular mechanisms underlying complex cellular signaling events, and the principles described here may well apply more generally.

## Experimental Procedures

Full experimental procedures for cloning, expression, and purification of deuterated, back-labeled protein samples for NMR spectroscopy (including those having different isotope labeling patterns in different domains and prepared using sortase ligation), DNA preparation and purification, isothermal calorimetry, fluorescence anisotropy, analytical ultracentrifugation, activity assays, live-cell imaging, and NMR experiments and assignments are provided in the Supplemental Experimental Procedures. The strategy used here for determining the structure of the F1F2-dumbbell complex was based on a combination of recently published methods and was developed specifically for this project. Because it has not been published previously, in the following we describe the approach. More detailed descriptions are given in the Supplemental Experimental Procedures.

### Structure Determination

Analysis of residual dipolar coupling (RDC) and NOE data showed that the internal structures of F1 and F2 are preserved on DNA complex formation and are similar to those determined previously by NMR spectroscopy (Eustermann et al., 2011) and crystallography (Langelier et al., 2011). For both F1 and F2, the match between experimental RDCs and those back-calculated using these crystal structures (PDB: 3ODA for F1 and 3ODC for F2) showed that the structures were equivalent to approximately the degree expected for X-ray structures in this resolution range (2.6–2.8 Å) (Bax and Grishaev, 2005). The Q values (calculated as Q = root-mean-square [RMS](D_i_^obs^-D_i_^calc^)/RMS(D_i_^obs^)) for F1 and F2 were 18.9% and 29.9%, respectively (Figure S2n). In addition, the many intra-finger NOEs observed and assigned in spectra of the complex were fully consistent with the previously known structures (Eustermann et al., 2011, Langelier et al., 2011). For the DNA, extensive comparison of free- and bound-state NOE contacts as well as observation of imino NH signals from all the AT and most of the GC base pairs showed that the two DNA stems preserved their conformation in the complex (Figure S3). In the absence of deposited DNA structures for the tetraloop sequences, RNA tetraloops 1MSY (Correll and Swinger, 2003) and 1RNG (Jucker and Pardi, 1995) were used as a basis for modeling. NOE contacts measured for the DNA dumbbell in both free and bound states (Figure S3) are largely consistent with these structures (except that the T35 base is in the anti conformation in the DNA case; Supplemental Experimental Procedures).

NOE contacts, CSP, and TRACT (Lee et al., 2006) data for the F1F2-DNA complex showed clearly that the dumbbell accommodates simultaneous binding of both fingers, with F2 binding the 3′ DNA stem and F1 the 5′ stem. Nine intermolecular NOE contacts were assigned, linking the 3′-terminal nucleotide G45 to residues Leu151 and Ile154 of F2 and five others linking C22 on the 5′ stem to residue Val48 of F1 (Figure 1F; Figure S2, Table S2). Additional evidence for this arrangement came from comparisons of amide group TROSY spectra of complexes of F1F2 with different DNA dumbbells. Changes in the DNA 3′ stem sequence caused CSPs almost exclusively in signals from F2, whereas changes in the 5′ stem sequence caused CSPs almost exclusively in signals from F1 (Figure S1).

The NOE and CSP data further established that the binding mode of F1 to the 5′ DNA stem and of F2 to the 3′ DNA stem in solution were both highly similar to that seen in both crystal structures of the individual PARP-1 F1 and F2 fingers, each bound to similar sites on DNA blunt ends (PDB: 3ODA and 3ODC, respectively) (Langelier et al., 2011). In both cases, the observed intermolecular NOE contacts described above exactly parallel contacts seen in the crystal structures (Figure 1F; Table S2). For F1 to bind to the DNA 5′ stem in the same way as F2 binds to the 3′ stem requires that the continuous DNA strand linking the stems must adopt a conformation very far from B-form if it is to avoid steric clashes with the protein, and a clear sign confirming it does so was provided by three intermolecular NOE contacts connecting Val48 to the linking nucleotide, T23. Further evidence for the overall interaction mode of fingers with stems comes from CSPs observed both for protein and DNA signals on complexation that parallel the interfaces seen in the crystal structures. These include large upfield shifts for both Phe44 and G1, strongly suggesting that they are mutually stacked, as well as CSPs on the DNA consistent with insertion of Arg18 of F1 and Arg122 of F2 into the DNA minor groove (Figures 2A and 2B).

To test whether F1 and F2 form a direct F1-F2 interaction in the complex, which could play an important role in determining the mutual orientation of the fingers, sortase-ligated constructs having different labeling in each finger were used to allow selective measurement of NOE contacts between F1 and F2 freed from overlap with other signals (Figure S2). This revealed a network of interdomain NOE contacts linking Pro149, Met153, and Val144 on F2 to Leu8, Ile37, Val39, and Val60 on F1 (Figure 1F; Figure S2; Table S2).

These data were used together to calculate a structure of the complex using a hybrid approach combining information from X-ray crystallography and NMR spectroscopy, using the program XPLOR-NIH (Schwieters et al., 2003). Because the NMR data clearly established that F1 and F2 bind to the 5′ and 3′ stems of the DNA dumbbell, respectively, and that the internal structures of the fingers, DNA stems, and the interfaces between each finger and its associated stem were very similar to those in crystal structures 3ODA and 3ODC (although in a different overall context), the calculations were restrained to reproduce these features by using non-crystallographic symmetry (NCS) terms in the force field (relative to fixed copies of starting structures in which these features were present; Supplemental Experimental Procedures). Fitting RDC data for both fingers to a single optimized alignment tensor using the implicit Saupe tensor alignment constraint (ISAC) protocol of Sass et al. (2001) defined the orientation of the fingers with respect to one another, while the NOE-derived inter-finger distance restraints defined the structure of their mutual interface, and intermolecular NOE-derived restraints helped define the DNA conformation at the gap. Regions of the complex for which there was no a priori information (e.g., the DNA linker joining the two stems), where the two fingers make contact, or that were shown to be dynamic by NMR relaxation experiments (e.g., the N- and C-terminal tails and the interfinger linker; Figure 1) were treated as being flexible. This was achieved in different ways in each case. The dihedrals of the DNA linker were randomized in the starting structures, for atoms of the F1-F2 interface the NCS force constant was reduced to a low value, and atoms of the protein linker were added in a second calculation step only after the relative positions of the fingers had been defined. Using a simulated annealing protocol similar to a conventional NMR structure determination, we were thus able to combine all of this information and determine an ensemble of structures consistent with the data, the result of which is shown in Figure 1E, with statistics summarized in Table 1 (see Supplemental Experimental Procedures for further description). The backbone precision across the ordered regions is approximately 0.34 Å, implying that the NMR-based constraints define the overall architecture quite closely. As with essentially any solution structure, the calculated ensemble spread reflects the precision with which the mean structure has been established from averaged data. In general, it does not necessarily provide a measure of the true spread in solution. However, the fact that we observed interdomain NOE contacts with reasonable strength shows that there must be a significant population of structures in solution in which these contacts are present, and cross-validation with SAXS measurements confirms independently that the overall shape of the complex is accurate (Figure 1H).

## Author Contributions

D.N. and S.E. designed the project. S.E., W.F.W., and L.E.E. cloned, expressed, and purified proteins for NMR. S.E., W.F.W., and J.C.Y. designed, collected, and processed NMR experiments. S.E., W.F.W., J.C.Y., and D.N. analyzed and assigned NMR data. D.N. and S.E. designed and carried out structural calculations and analyses. S.E. and W.F.W. carried out and analyzed ITC experiments and SAXS experiments. M.F.L. and J.M.P. designed and performed the in *cis* modification and AUC experiments. M.F.L. expressed and purified proteins and performed biochemical analysis of full-length PARP-1 and mutants. A.A.R. performed live-cell imaging experiments. S.E., M.F.L., J.M.P., and D.N. wrote the manuscript.

## Figures and Tables

**Table 1:** Structural Statistics for the Ensemble of 78 Accepted Structures of the PARP-1 F1F2 Dumbbell Complex

**Template Restraints, Protein (Supported by NMR Data; see Experimental Procedures)**	

Strong NCS (force constant 10^4^ kcal.mol^−1^) to 3ODA	F1, res. 9–36, 49–59, and 66–89 (all atoms)
Weak NCS (force constant 50 kcal.mol^−1^) to 3ODA	F1, res. 6–8, 37–48, 60–65, and 90–91 (N, Cα, C′), res. 44 (all carbons)
Strong NCS (force constant 10^4^ kcal.mol^−1^) to 3ODC	F2, res. 114–140 and 155–199 (all atoms)
Weak NCS (force constant 50 kcal.mol^−1^) to 3ODC	F2, res. 109–113, 141–154, and 200–201 (N, Cα, C′)

**Template Restraints, DNA (Supported by NMR Data; see Experimental Procedures)**	

Distance (O3′, O5′, intra- and inter-strand)	stem 1, 48; stem 2, 48
Dihedral angle	stem 1, 193; stem 2, 168
tetraloops, 95
Base pair H-bond distance	stem 1, 24 (6 GC, 3 AT base pairs)
stem 2, 25 (7 GC, 2 AT base pairs)
tetraloops, 6 (2 base pairs)

**NMR-Derived Restraints on Domain Interactions and Orientation**	

Interdomain NOE-derived distances	6 (from 15 NOEs; Table S2)
Intermolecular NOE-derived distances	12 (from 17 NOEs; Table S2)

**RDCs (NH)**	

F1	42
F2	43

**XPLOR-NIH Energy Terms (kcal.mol^−1^)**	

E(total)	4159 ± 714
E(tensor)	1123 ± 4.0
E(distance)	0.70 ± 0.39
E(NCS)	268 ± 8
E(VDW)	973 ± 499

**Violations**	

NOE (max, mean ± SD)	0.177 Å, 0.046 ± 0.035 Å
Q_RDC_ (mean ± SD)	24.47% ± 0.04%

**Deviations from Ideal Geometry (RMSD)**	

Bonds	0.0042 Å
Angles	0.780°
Impropers	0.656°

**Protein Ramachandran Statistics**	

Residues 6–91, 109–201	F1: 91.7%, 7.5%, 0.8%, 0.0%
(core, allowed, generously allowed, disallowed)	F2: 87.0%, 13.0%, 0.0%, 0.0%

**Co-ordinate Precision (Mean RMSD to Mean Structure)**^b^	

F1, F2, whole DNA (all heavy)	0.524 ± 0.172 Å
F1, F2, whole DNA (backbone)	0.352 ± 0.205 Å
F1, F2, DNA stem 1, DNA stem 2 (backbone)	0.340 ± 0.211 Å
F1, DNA stem1 (backbone)	0.036 ± 0.027 Å
F2, DNA stem 2 (backbone)	0.042 ± 0.025 Å

Abbreviations: res, residues; VDW, van der Waals; RMSD, root-mean-square deviation. For further details, see Supplemental Experimental Procedures. ^a^Prior to addition of disordered protein residues 1–5, 92–108, and 202–214.

b F1, residues 6–91; F2, residues 109–201; DNA stem 1, nt 1–22; DNA stem 2, nt 24–45 (excluding C24 O5′ and P).

## Accession Numbers

The co-ordinates for the ensemble of 78 structures of the F1F2-DNA dumbbell complex reported in this paper have been deposited in the PDB under ID code PDB: 2n8a. Chemical shift assignments are reported as follows: F1F2-DNA complex, BMRB: 25888; F1F2 free, BMRB: 25889; DNA free, BMRB: 25890; F1F2F3-DNA complex, BMRB: 25891; F1F2F3 free, BMRB: 25892; F3 free, BMRB: 25893; F1F2F3-WGR-DNA complex, BMRB: 25894; WGR free, BMRB: 25895.

## Supplemental Information

Document S1. Supplemental Experimental Procedures, Figures S1–S5, and Tables S1 and S2

Document S2. Article plus Supplemental Information
