# Supplementary material for: Structural Basis of Detection and Signaling of DNA Single-Strand Breaks by Human PARP-1
Source: Mol Cell. 2015 Dec 3;60(5):742–54. doi: 10.1016/j.molcel.2015.10.032 (PMC4678113; doi:10.1016/j.molcel.2015.10.032)
Supplement: Document S2. Article plus Supplemental Information [file mmc2.pdf]

Article

Molecular Cell

Structural Basis of Detection and Signaling of DNA Single-Strand Breaks by Human PARP-1

Graphical Abstract

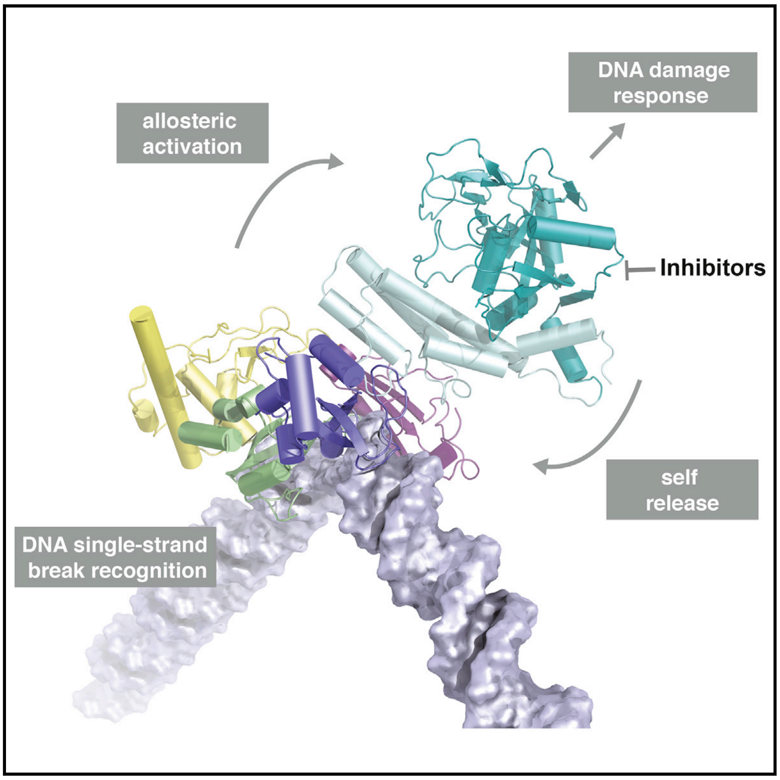

Authors

Sebastian Eustermann, Wing-Fung Wu, Marie-France Langelier, ..., Amanda A. Riccio, John M. Pascal, David Neuhaus

Correspondence

dn@mrc-lmb.cam.ac.uk

In Brief

Eustermann et al. reveal the structural basis for recognition of DNA single-strand breaks by human PARP-1 and how this drives a multi-domain folding cascade to operate the allosteric switch initiating DNA damage signaling. The structural model of PARP-1 on a DNA break helps rationalize inhibitor action.

Highlights

- We identify the structural basis of DNA single-strand break detection by PARP-1
- An NMR/X-ray approach reveals how multi-domain allostery underlies activity switch
- The molecular model of PARP-1 assembly couples damage detection to PAR signaling
- Automodification in *cis* releases enzyme so DNA repair and replication can proceed

Accession Numbers

2n8a

Eustermann et al., 2015, Molecular Cell *60*, 742–754
December 3, 2015 ©2015 The Authors
http://dx.doi.org/10.1016/j.molcel.2015.10.032

Molecular Cell
Article

Structural Basis of Detection and Signaling of DNA Single-Strand Breaks by Human PARP-1

Sebastian Eustermann,[1,3] Wing-Fung Wu,[1,4] Marie-France Langelier,[2,5] Ji-Chun Yang,[1] Laura E. Easton,[1] Amanda A. Riccio,[2,5] John M. Pascal,[2,5] and David Neuhaus[1,*]
[1]Medical Research Council, Laboratory of Molecular Biology, Francis Crick Avenue, Cambridge CB2 0QH, UK
[2]Department of Molecular Biology and Biochemistry, Sidney Kimmel Cancer Center, Thomas Jefferson University, 233 South 10th Street, Bluemle Life Sciences Building, Room 804, Philadelphia, PA 19107, USA
[3]Present address: Gene Center and Department of Biochemistry, Ludwig-Maximilians-University Munich, Feodor-Lynen-Strasse 25, 81377 Munich, Germany
[4]Present address: Centre of Influenza Research, School of Public Health, Li Ka Shing Faculty of Medicine, The University of Hong Kong, 21 Sassoon Road, Pokfulam, Hong Kong, China
[5]Present address: Department of Biochemistry and Molecular Medicine, Université de Montréal, Montréal, QC H3C 3J7, Canada
*Correspondence: dn@mrc-lmb.cam.ac.uk
http://dx.doi.org/10.1016/j.molcel.2015.10.032
This is an open access article under the CC BY license (http://creativecommons.org/licenses/by/4.0/).

SUMMARY

Poly(ADP-ribose)polymerase 1 (PARP-1) is a key eukaryotic stress sensor that responds in seconds to DNA single-strand breaks (SSBs), the most frequent genomic damage. A burst of poly(ADP-ribose) synthesis initiates DNA damage response, whereas PARP-1 inhibition kills BRCA-deficient tumor cells selectively, providing the first anti-cancer therapy based on synthetic lethality. However, the mechanism underlying PARP-1's function remained obscure; inherent dynamics of SSBs and PARP-1's multi-domain architecture hindered structural studies. Here we reveal the structural basis of SSB detection and how multi-domain folding underlies the allosteric switch that determines PARP-1's signaling response. Two flexibly linked N-terminal zinc fingers recognize the extreme deformability of SSBs and drive co-operative, stepwise self-assembly of remaining PARP-1 domains to control the activity of the C-terminal catalytic domain. Automodifcation in *cis* explains the subsequent release of monomeric PARP-1 from DNA, allowing repair and replication to proceed. Our results provide a molecular framework for understanding PARP inhibitor action and, more generally, allosteric control of dynamic, multi-domain proteins.

INTRODUCTION

Poly(ADP-ribose)polymerase 1 (PARP-1) is a highly abundant chromatin-associated protein found in the nuclei of all higher eukaryotes. It is the founding member of a family of enzymes that modify a wide variety of target proteins with poly(ADP-ribose) (PAR), a highly negatively charged, branched-chain posttranslational modification derived from NAD^+ (D'Amours et al., 1999; Krishnakumar and Kraus, 2010). Acute DNA damage-dependent activation of PARP-1, the major PAR-producing enzyme in eukaryotes, is one of the earliest cellular responses to genotoxic stress (Polo and Jackson, 2011) and links DNA damage response signaling and recruitment of DNA repair factors to a concerted modulation of chromatin structure (Durkacz et al., 1980; Schreiber et al., 2006; Chou et al., 2010). Excessive activation leads to cell death through NAD^+ depletion (Fouquerel et al., 2014), whereas basal levels are required for other key functions such as transcriptional regulation (Kim et al., 2004; Schreiber et al., 2006). PARP-1 is central to the cellular stress response and has been implicated in a number of pathophysiological conditions (Luo and Kraus, 2012). Most prominently, it has emerged as an important target for cancer therapy (Bryant et al., 2005; Farmer et al., 2005). Numerous PARP inhibitors are in clinical trials, and, very recently, the first, olaparib (AstraZeneca), has been approved for treatment of advanced BRCA-dependent ovarian cancer (Sonnenblick et al., 2015). These inhibitors represent an entirely novel class of cancer therapeutics based on synthetic lethality. A collapse of genome integrity caused by the cumulative effects of PARP inhibition and defective homologous recombination (HR) repair kills BRCA-deficient tumor cells, whereas cells with intact HR repair are largely unaffected by PARP inhibitors under normal conditions. Similar effects involving other repair pathway deficiencies are now also coming to light (Murai et al., 2012; Mendes-Pereira et al., 2009), and possible causes of resistance to PARP inhibitors are being investigated (Lord and Ashworth, 2013). However, a major obstacle in understanding PARP-1's role during genomic maintenance and the cause of the synthetic lethal effect of inhibitors (Helleday, 2011) has been that the molecular basis of its function remains poorly understood.

DNA single-strand breaks (SSBs) are by far the most frequent form of DNA damage, resulting both directly from oxidative damage and as intermediates in other DNA repair pathways (Caldecott, 2008). Although repair of such chemically diverse DNA lesions has been much studied, the detailed structural mechanism by which they are efficiently detected and signalled

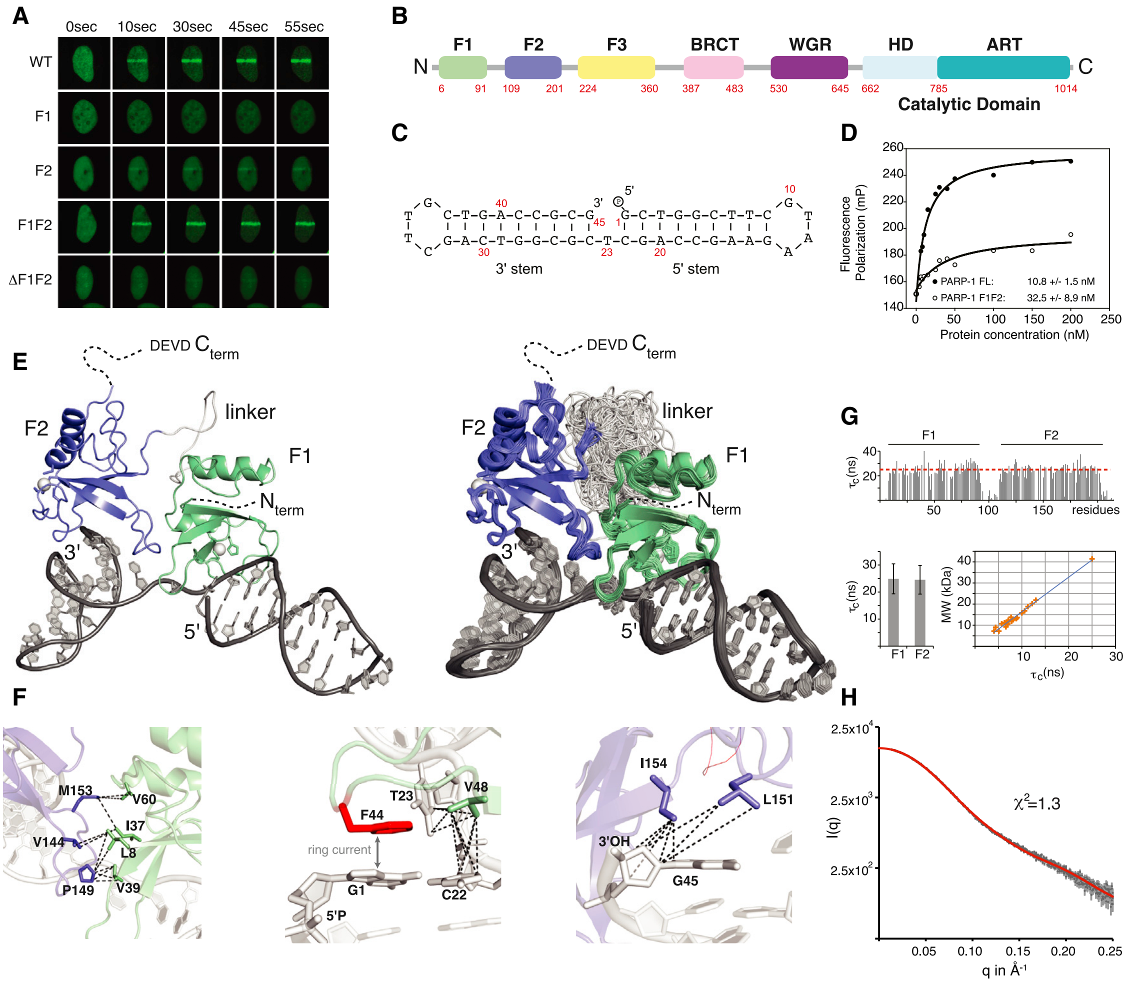

Figure 1. Structural Basis of SSB Recognition by PARP-1

(A) Live-cell imaging shows recruitment of GFP-labeled PARP-1 and PARP-1 fragments to sites of laser-induced DNA damage. WT, wild-type.

(B) Domain structure of PARP-1.

(C) Gapped dumbbell DNA ligand used in this work as a mimic of an SSB.

(D) Fluorescence polarization experiments show that F1F2 binds the DNA ligand only about 3-fold less strongly than full-length PARP-1. FL, full-length.

(E) NMR/X-ray hybrid structure of F1F2 bound to an SSB. Overall views of (left) the lowest-energy structure, and (right) the ensemble of all 78 accepted structures (see Experimental Procedures and Supplemental Experimental Procedures for details of structure determination).

(F) Measured NOE contacts (dashed lines; Table S2) that define the F1-F2 interface (left), the hydrophobic interactions of F1 with the 5′ stem and T23 (center), and of F2 with the 3′ stem (right). The stacking interaction between F44 (red) and G1 was inferred from strong CSPs caused by their aromatic ring currents.

(G) Effective τ_c values obtained from ^{15}N relaxation (TRACT) experiments with the F1F2 complex are consistent with a 40-kDa species (1:1 stoichiometry) in which both fingers bind simultaneously and the linker remains flexible.

(H) The experimental SAXS profile of the PARP-1 F1F2 dumbbell-DNA complex (3 mg.ml^{-1}) agrees with the back-calculated SAXS profile averaged over the ensemble shown in (E).

See also Figures S1, S2, S3, and S4 and Tables S1 and S2.

to the DNA repair machinery has remained elusive. PARP-1 has long been known as a crucial first-line sensor of SSBs (de Murcia and Ménissier de Murcia, 1994; Satoh and Lindahl, 1992), and functional studies have consistently highlighted its physiological importance as well as its central role for PARP inhibitor action (Helleday, 2011; Bryant et al., 2005; Caldecott, 2014; Murai et al., 2012). Despite the diversity of SSBs, PARP-1 recruitment to sites of genomic damage and PAR-mediated signaling must be both rapid and robust while, at the same time, maintaining the dynamic range and tight control required for PARP-1's cellular function. PARP-1 comprises six domains connected by flexible linkers (Figure 1B), and, in the free state, these domains are independent, behaving like "beads on a string" (Lilyestrom et al., 2010). Recent crystal structures of different combinations of domains from PARP-1 bound to the ends of short DNA duplexes as mimics of DNA double-strand breaks (DSBs) provided important insights by showing the existence of individual domain-domain interactions required for activation (Langelier et al., 2012; Ali et al., 2012). However, these static views did not establish the mechanism by which the observed interactions

arise or show whether they would form a rapid yet adjustable switch for PARP-1 activation. Mutually exclusive DNA-binding modes were observed, leading to substantially different proposals regarding damage recognition and activation. As for many key signaling proteins, PARP-1's highly dynamic and modular architecture has hindered structural studies. However, it is likely that these dynamics underlie the way PARP-1 achieves its complex roles in genome maintenance. Recent studies of other systems have shown the importance of allosteric and cooperative effects within highly dynamic multi-domain proteins in defining cellular responses, although the principles underlying such effects are only now emerging (Chao et al., 2011; Mackereth et al., 2011).

Here we used an integrated nuclear magnetic resonance (NMR)/X-ray approach to establish the molecular mechanism by which PARP-1 senses SSBs and becomes allosterically activated. Because key interactions are detected in solution using NMR, inherent flexibility no longer poses the obstacle that it does for crystallography, allowing us to interrogate complexes representing successive assembly states of the system and, thereby, build up the stepwise, co-operative, multi-domain folding pathway that underlies the operation of PARP-1's DNA damage-dependent activity switch during genomic maintenance.

RESULTS AND DISCUSSION

Capturing Recognition of DNA Single-Strand Breaks In Vitro

To understand the molecular basis of the PARP-1 response to genomic damage, we first sought to dissect how DNA damage is recognized. While crystallographic studies so far have employed mimics of DSBs, we set out to determine the structural basis of SSB recognition using solution-state NMR spectroscopy. We used a minimal protein construct comprising PARP-1's flexibly linked N-terminal zinc fingers F1F2 (Figure 1B), corresponding to the naturally occurring caspase-3 cleavage product of PARP-1. Our live-cell imaging experiments show that, similar to full-length PARP-1, F1F2 localizes within seconds to sites of laser-induced DNA damage, whereas isolated F1 or F2 domains showed no recruitment or only residual levels, respectively (Figure 1A). This shows that the two fingers must act co-operatively, corroborating previous functional studies (de Murcia et al., 1994; Molinete et al., 1993) and the mutational analysis by Ali et al. (2012). To recapitulate these features of PARP-1 recruitment in vitro, we employed our previously established model system for DNA single-strand breaks (Figure 1C; Eustermann et al., 2011). Given the micromolar concentration of PARP-1 inside eukaryotic nuclei (D'Amours et al., 1999), the measured nanomolar affinities for this ligand are in agreement with PARP-1's function as a bona fide DNA damage sensor, and F1F2 binds SSBs only slightly less strongly than does full-length PARP-1 (Figure 1D). The interaction is sequence-independent (Figure S1), DNA-damage specific, and co-operative. Binding of isolated F2 to the DNA dumbbell is approximately 10-fold weaker than for F1F2, and F1 is much weaker still (Eustermann et al., 2011). Notably, isothermal calorimetry (ITC) (Figure 2C) and transverse relaxation-optimized spectroscopy (TROSY) for rotational correlation times (TRACT) NMR (Figure 1G) experiments confirmed that SSB recognition occurs as a monomer with a 1:1 stoichiometry (Lilyestrom et al., 2010; Eustermann et al., 2011) and showed that both fingers are bound simultaneously, consistent with their cooperative role in vitro and in vivo. Previously reported models based on DNA duplexes as mimics of DSBs have either suggested that cooperative action of F1 and F2 results in DNA damage-induced dimerization (Ali et al., 2012) or did not include F2 but have suggested monomeric DSB activation (Langelier et al., 2012). Based on our findings, we concluded that a complex of F1F2 with dumbbell DNA represents a minimal structural unit required to capture the first stage of PARP-1's interaction with SSBs.

The challenges of determining a structure of this size by NMR were overcome mainly by combining TROSY-based experiments (Fernández and Wider, 2003) with a targeted isotope-labeling strategy and, where necessary, using ligation via the enzyme sortase A (Kobashigawa et al., 2009) to produce chains with different isotopic labeling patterns in different domains to reduce spectral overlap and facilitate interpretation. A similar approach was used to make signal assignments for the larger complexes described later in this paper. This approach allowed us to obtain extensive protein and DNA assignments and measure key structural information such as residual dipolar couplings (Figures S4a–S4c), protein backbone dynamics (Figure 1G; Figure S5f), and assigned nuclear Overhauser effect (NOE) contacts within domains and at the domain-domain and protein-DNA interaction interfaces (Figure 1F; Figures S2d–S2k). Intriguingly, our NMR data identified local contacts of F1 and F2 with DNA that were analogous to those observed in previous crystal structures of the isolated fingers on DNA blunt ends (Langelier et al., 2011) despite occurring in a structurally different context. Building on related approaches for characterizing flexible multi-domain proteins and their complexes (Mackereth et al., 2011; Göbl et al., 2014), we were therefore able to incorporate direct knowledge of these crystal structures while our NMR data provided the key information to determine the overall structure as well as other aspects of the system where flexibility poses problems for crystallography (Experimental Procedures; Supplemental Experimental Procedures; Table 1; Table S2).

Structural Basis of DNA Single-Strand Break Recognition

The most striking feature of the determined structure of F1F2 bound to the gapped DNA dumbbell (Figure 1E) is the way in which binding of the two flexibly linked finger domains on either side of the break opens up the structure of the DNA. It is immediately clear that undamaged double-helical DNA could never adopt such a conformation. The fingers, and, consequently, also the DNA stems to which they are bound, become mutually oriented through acquisition of a small but defined DNA-dependent, hydrophobic F1-F2 interface (average area, 359 ± 35 Å^2) (identified by NOE contacts; Figure 1F; Figures S2e–S2g), and they adopt a single directionality on the DNA with F2 on the 3′ stem and F1 on the 5′ stem (as evidenced by the data in Figures 1F, 2A, and 2B), whereas the linker remains flexible in the complex (Figure 1G). The average bend angle of the DNA around the break (approximately 107° ± 1° across the ensemble)

Table 1. Structural Statistics for the Ensemble of 78 Accepted Structures of the PARP-1 F1F2 Dumbbell Complex

**Template Restraints, Protein (Supported by NMR Data; see Experimental Procedures)**	
Strong NCS (force constant 10^4 kcal.mol^{-1}) to 3ODA	F1, res. 9–36, 49–59, and 66–89 (all atoms)
Weak NCS (force constant 50 kcal.mol^{-1}) to 3ODA	F1, res. 6–8, 37–48, 60–65, and 90–91 (N, Cα, C′), res. 44 (all carbons)
Strong NCS (force constant 10^4 kcal.mol^{-1}) to 3ODC	F2, res. 114–140 and 155–199 (all atoms)
Weak NCS (force constant 50 kcal.mol^{-1}) to 3ODC	F2, res. 109–113, 141–154, and 200–201 (N, Cα, C′)
**Template Restraints, DNA (Supported by NMR Data; see Experimental Procedures)**	
Distance (O3′, O5′, intra- and inter-strand)	stem 1, 48; stem 2, 48
Dihedral angle	stem 1, 193; stem 2, 168
	tetraloops, 95
Base pair H-bond distance	stem 1, 24 (6 GC, 3 AT base pairs)
	stem 2, 25 (7 GC, 2 AT base pairs)
	tetraloops, 6 (2 base pairs)
**NMR-Derived Restraints on Domain Interactions and Orientation**	
Interdomain NOE-derived distances	6 (from 15 NOEs; Table S2)
Intermolecular NOE-derived distances	12 (from 17 NOEs; Table S2)
**RDCs (NH)**	
F1	42
F2	43
**XPLOR-NIH Energy Terms (kcal.mol^{-1})**	
E(total)	4159 ± 714
E(tensor)	1123 ± 4.0
E(distance)	0.70 ± 0.39
E(NCS)	268 ± 8
E(VDW)	973 ± 499
**Violations**	
NOE (max, mean ± SD)	0.177 Å, 0.046 ± 0.035 Å
Q_{RDC} (mean ± SD)	24.47% ± 0.04%
**Deviations from Ideal Geometry (RMSD)**	
Bonds	0.0042 Å
Angles	0.780°
Impropers	0.656°
**Protein Ramachandran Statistics**	
Residues 6–91, 109–201	F1: 91.7%, 7.5%, 0.8%, 0.0%
(core, allowed, generously allowed, disallowed)	F2: 87.0%, 13.0%, 0.0%, 0.0%
**Co-ordinate Precision (Mean RMSD to Mean Structure)[b]**	
F1, F2, whole DNA (all heavy)	0.524 ± 0.172 Å
F1, F2, whole DNA (backbone)	0.352 ± 0.205 Å
F1, F2, DNA stem 1, DNA stem 2 (backbone)	0.340 ± 0.211 Å
F1, DNA stem1 (backbone)	0.036 ± 0.027 Å
F2, DNA stem 2 (backbone)	0.042 ± 0.025 Å

Abbreviations: res, residues; VDW, van der Waals; RMSD, root-mean-square deviation. For further details, see Supplemental Experimental Procedures. [a]Prior to addition of disordered protein residues 1–5, 92–108, and 202–214.

[b]F1, residues 6–91; F2, residues 109–201; DNA stem 1, nt 1–22; DNA stem 2, nt 24–45 (excluding C24 O5′ and P).

matches closely early positive-stain electron microscopy measurements (102° ± 44°) (Le Cam et al., 1994) and was cross-validated further by our measured small-angle X-ray scattering (SAXS) data that showed excellent agreement with the back-calculated scattering curve of the structural ensemble (Figure 1H). Not only is a severe kink induced at the SSB, but, crucially, the two stems are also twisted apart in such a way that the faces of all four flanking bases are exposed to allow interactions with the protein. Intriguingly, the two fingers each show a highly similar mode of interaction with their respective stems. The observed NOE and chemical shift perturbation (CSP) data clearly show that local interactions of F1 and F2 with DNA bases and adjacent minor grooves show the same pattern on both sides of the break (Figures 1F, 2A, and 2B Table S2), closely paralleling those observed in crystal structures of isolated fingers, each bound across the 3′ terminus of a DNA stem (Langelier et al., 2011). However, although such observations from the crystal structures might have suggested that binding of the fingers was mutually exclusive, our solution structure shows how the flexibility of an SSB accommodates the simultaneous binding of F2 and F1 despite the asymmetric nature of the 5′ and 3′ stems (Figures 1E–1G). Steric clashes between the fingers are avoided as the continuous DNA strand linking the stems adopts a conformation very far from B-form. In effect, the F1 binding site is cryptic, becoming exposed only as a result of the severe distortions of the linking strand imposed by binding of F2 to the other site. The resulting overall protein-DNA interface is large enough to provide PARP-1 with sufficient affinity and specificity for F1F2 binding, explaining the cooperative role of the fingers in vivo (Figure 1A). Notably, neither finger makes direct contacts with the DNA termini (Figure 1F), consistent with the role of PARP-1 as a first-line sensor of a wide variety of chemically diverse SSB types. This explains our previous observation that SSB detection by F1F2 is essentially independent of terminal modifications such as 3′ phosphorylation (a common result of oxidative DNA backbone damage) (Eustermann et al., 2011).

A key finding of our study is that F1F2 binds in only one direction on an SSB, and the direction it selects (F2 on the 3′ stem, F1 on the 5′) is the only one that triggers activation (Figure 3). Given the dynamic nature of the system, the similarity of the fingers and the absence of direct contacts with the termini, it is perhaps surprising that PARP-1 shows this directional selectivity on SSBs. Our study suggests three possible contributions. First, the F1-F2 interface we see can only form when the fingers bind in the observed sense. Second, the linker path in a hypothetical reversed complex would be much longer (e.g., swapping F1 and F2 would increase the distance A91Cα-T109Cα from ∼11 to

CellPress

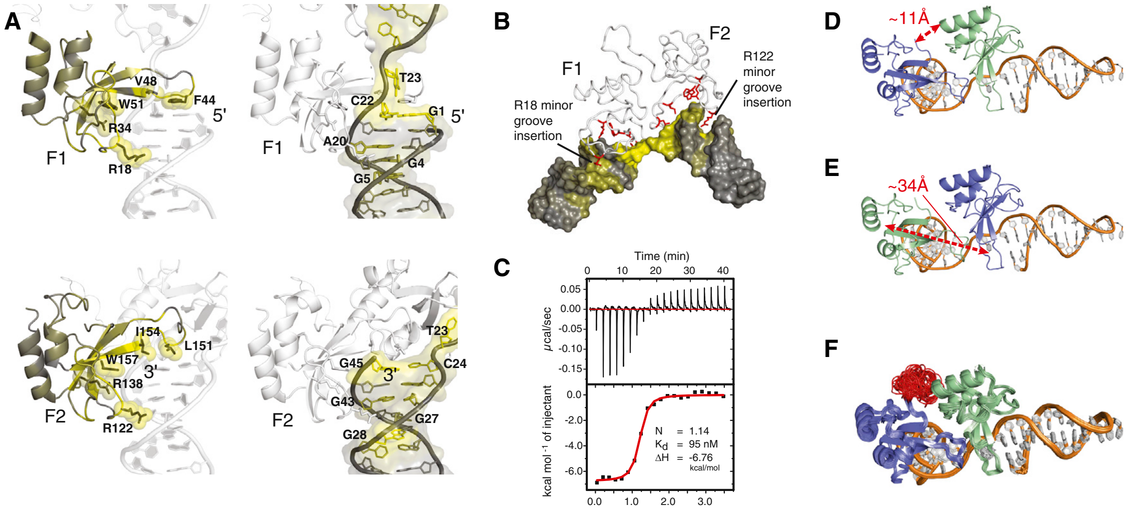

Figure 2. Interactions of F1 and F2 on Either Side of the SSB

(A) CSPs on DNA binding mapped to the structure for protein amide groups (left) and DNA 1′-CH groups (right), for the F1-5′ DNA stem interface (top), and for the F2-3′ DNA stem interface (bottom). See Figure S2 for details and definitions of gray to yellow color ramps. Large CSPs occur for the key interacting protein residues labeled. For the DNA, CSPs match the 7-base pair footprints of the fingers on either side of the break (de Murcia and Ménissier de Murcia, 1994), with the largest effects at the exposed stem ends (C22-G1 and C24-G45) and sites of arginine insertions into the minor groove (Arg122 of F2 near C27, G28, and G43 in the 3′ stem and Arg18 of F1 near G4, G5, and A20 in the 5′ stem).

(B) CSPs measured for the DNA on F1F2 binding, illustrating the largest perturbations that occur at the DNA damage site. Protein side chains interacting with the DNA backbone are shown in red.

(C) Isothermal calorimetry shows that high-affinity binding of PARP-1 F1F2 to the gapped DNA dumbbell ligand occurs with 1:1 stoichiometry and is fully saturated at higher protein:DNA ratios (note that the apparent K_d is unreliable under these stoichiometric conditions).

(D and E) Schematics showing how reversing the directionality of the protein on the SSB would affect the F1-F2 linker (which was shown to be flexible by NMR; Figure 1G). If F1 bound the 3′ stem and F2 the 5′, then the distance spanned by the linker would increase from ~11 Å in the actual complex (D) to ~34 Å in the hypothetical reversed complex (E).

(F) Further calculations show that the observed arrangement of F1 and F2 is also consistent with the artificially shortened linker (Δ94–102). Ali et al. (2012) have reported that PARP-1 Δ94–102 localizes to laser-induced DNA damage in a very similar manner as wild-type protein in vivo.

~34 Å), suggesting that the linker could act as an "entropic spring," favoring the shorter path (Figures 2D and 2E). Third, if F2, the finger with the higher affinity, wins the competition to bind first, then this will presumably direct it to the 3′ stem because DNA distortions required to reveal this site are much smaller than those required to reveal the "cryptic" second site on the 5′ stem.

Taken together, our results identify a consensus that many DNA structures that activate PARP-1 have in common, comprising two flexibly linked DNA stems with exposed bases at the ends, independent of 3′ or 5′ modifications. When F2 has initiated recognition by binding at the 3′ stem, subsequent scanning for the second site by the flexibly linked F1 domain resembles a "fly-casting" mechanism (Shoemaker et al., 2000), elegantly explaining how PARP-1 efficiently recognizes DNA single-strand breaks with different gap lengths. Interestingly, such structures may also exist at stalled replication forks, which also efficiently activate PARP-1, depending on the length of single-stranded region they contain (Bryant et al., 2009). Indeed, one may even speculate that PARP-1 could recognize DSBs by an analogous mechanism, provided the two stems at the DNA break are held in sufficiently close proximity either directly by PARP-1 binding or by DSB sensors that are known to tether DSB ends (e.g., the Mre11-Rad50-Nbs1 [MRN] complex; Moreno-Herrero et al., 2005). Under other circumstances, where a DNA damage site is more rigid, binding and activation become uncoupled. Others have shown, using pull-downs from cell lysate, that PARP-1 is a prominent sensor of abasic sites but that activation only occurs after apurinic/apyrimidinic (AP) endonuclease transforms such lesions into SSBs (Khodyreva et al., 2010). Detecting "DNA deformability" at damage sites is a mechanism seen in other DNA repair systems (Lamers et al., 2000; Min and Pavletich, 2007). However, unlike other repair factors, PARP-1 uses two flexibly linked recognition modules, and our data establish a distinct mechanism for versatile yet specific DNA damage recognition, providing a unified explanation of PARP-1's involvement in diverse DNA repair pathways.

DNA Damage Recognition Drives PARP-1's Allosteric Activity Switch

Having shown how PARP-1 recognizes SSBs, we sought next to understand how this leads to allosteric activation. Combination of our structure of F1F2 on an SSB with the previous crystal structures of F1, F3, and WGR-CAT on a DSB (Langelier et al., 2012) leads directly to a structural model of full-length PARP-1 assembled on an SSB (Figure 3A). Perhaps surprisingly, there are no steric clashes, and all domains can be linked in a single polypeptide chain, fully consistent with our analytical ultracentrifugation (AUC) data for full-length SSB-bound PARP-1

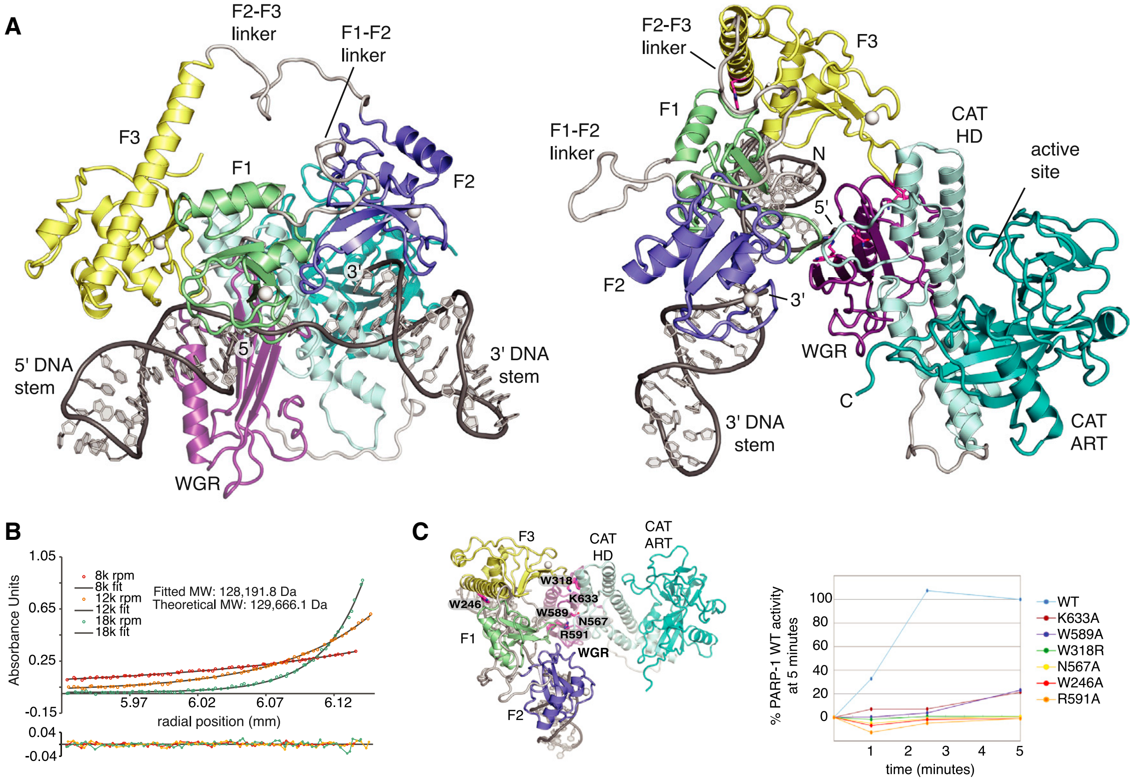

Figure 3. Structural Model of Full-Length PARP-1 Bound to a DNA Single-Strand Break

(A) Superposition of the hybrid structure of F1F2 bound to a DNA dumbbell and the previous crystal structure of F1, F3, and WGR-CAT bound to a DNA duplex (PDB: 4DQY) led directly to the domain arrangement shown (see also Figures S5g and S5h, Experimental Procedures, and Supplemental Experimental Procedures). The F1-F2 and F2-F3 linkers are flexible (see the NMR ^{15}N relaxation data in Figure S5f), whereas the BRCT domain (not required for activity) and its linkers are omitted but may also adopt a wide variety of locations. The structure of PARP-1 on an SSB was corroborated using biophysical and mutational analysis (see B and C), NMR spectroscopy that elucidated its dynamic assembly process (Figure 4), and HXMS experiments (Dawicki-McKenna et al., 2015).

(B) Analytical ultracentrifugation shows that full-length PARP-1 binds to the gapped DNA dumbbell with a 1:1 stoichiometry. MW, molecular weight.

(C) Catalytic activities of wild-type PARP-1 and the designated mutants were assessed using a colorimetric activity assay (Supplemental Experimental Procedures) using the dumbbell gap DNA as the activating ligand.

(Figure 3B). Previous crystallographic analysis of DSB recognition by F1F2 led to the suggestion that PARP-1 is activated by DNA-induced dimerization (Ali et al., 2012), a mechanism found in many DNA binding proteins and suggested previously for PARP-1. However, the monomeric arrangement of the two fingers on an SSB that we observe in solution differs substantially. Our data emphasize the critical importance of directional binding of F1 and F2 because only when F1 is positioned on the cryptic site on the 5′ stem can F1 subsequently interact with the F3 and WGR domains (Figure 3A). This is highly relevant because Langelier et al. (2012) observed a ternary interaction of the F3, WGR, and CAT domains that distorts the regulatory HD subdomain, thereby destabilizing CAT and priming it for productive catalysis. A separate study by Pascal, Black, and colleagues using hydrogen exchange mass spectrometry (HXMS) has now shown that the HD subdomain can undergo local unfolding to release an unanticipated inhibitory effect (Dawicki-McKenna et al., 2015 [this issue of *Molecular Cell*]). Intriguingly, there are no direct contacts in our model between the CAT domain and F1, F2, or the DNA, showing that communication must occur through F3 and WGR. Our mutational analysis shows that interactions of the latter domains within monomeric PARP-1 are essential for activation by SSBs (Figure 3C). Taking this together, we hypothesized that the manner in which the domains of PARP-1 fold up onto an SSB must provide the free energy responsible for destabilizing CAT and that it is this process that underlies the rapid and robust, yet tunable, operation of the of the switch controlling PARP-1 activity.

Because static views of fully assembled PARP-1 complexes do not reveal the nature of such events, we turned again to NMR spectroscopy to follow the DNA-induced folding of PARP-1 by identifying and characterizing possible intermediate steps. Comparative analysis of DNA-dependent chemical shift perturbations (CSPs) clearly revealed that SSB binding by F1F2 triggers interactions of F3 with both F1 and the 5′ stem of the DNA (Figure 4). However, these interactions are delicately poised. They occur only when F3 is covalently linked to F1F2 (data not shown) and require correct spatial pre-organization of the contact surfaces on F1 and the DNA so that both interfaces to F3 can form simultaneously. ^{15}N relaxation NMR experiments show that the F1-F2 and F2-F3 linkers are both highly flexible in the 56-kDa complex (Figure S5f) and also show that the F3

domain is not as rigidly associated with the rest of the complex as F1 or F2. Measured ^{15}N $R_{1\rho}$ rates for F3 are slower than those for F1 or F2, suggesting a looser, more transient interaction. In fact, a single point mutation of F3 at the F1 interaction surface can completely release F3 from F1 and the DNA. All of the CSPs characteristic of the F3-F1 and F3-DNA interactions are missing for F1F2F3 W246A (Figure S5e), and, importantly, the same mutation abolishes activation of full-length PARP-1 (Figure 3C). The arrangement of F1F2 on the SSB is also responsible for directing assembly of the WGR domain. The tip of the F1 base-stacking loop and the 5′ DNA terminus together create a composite surface for WGR interaction, and these interactions are strong enough to observe as CSPs in NMR titrations of the WGR domain with pre-assembled F1F2F3-SSB, resulting in a 71-kDa complex (however, the small difference in affinities of full-length PARP-1 and F1F2 for the SSB suggest that WGR binding must still be relatively weak; Figure 1D). Not only do these experiments show significant CSPs at points of direct contact that are required for activity (Figure 4B), but, also, many of the SSB-dependent CSPs already observed for the other interfaces of F1F2F3 become more pronounced upon addition of WGR, showing that WGR binding causes co-operative strengthening of inter-domain interactions throughout the complex (Figure 4C).

By showing how high-affinity SSB detection by F1F2 provides the driving force to bring together F3 and WGR in the correct spatial orientation in the absence of CAT, we reveal intermediate steps on a multi-domain folding pathway that explain why CAT binding and consequent destabilization take place, initiating productive PAR catalysis. Association of each domain is required to create the binding platform for the next. Because individual interactions subsequent to DNA damage recognition are weak and are built from small parts on separate components, these must be pre-organized by previous assembly steps to form an organized whole (Figure 5). Our data provide direct insights into this process, showing how each step reduces the conformational space of the system, ultimately reducing the entropic cost of the ternary F3, WGR, and CAT interaction and providing the free energy for CAT destablization. In contrast, in the absence of DNA, pre-organization among the domains is missing, the resulting partial inter-domain interfaces are not individually strong enough to form, except perhaps transiently, and, consequently, PARP-1 remains inactive. The co-operative nature of this DNA-induced self-assembly process ensures that PARP-1 is robustly switched between inactive and active states. The process has parallels to protein folding and is an example of what Hunter and Anderson (2009) termed “chelating co-operativity.” Furthermore, by identifying the underlying intermediate steps and their dynamics, we provide a framework for understanding the selective regulation of PARP-1. For instance, caspase-3 inactivation of PARP-1, a hallmark of apoptosis (Lazebnik et al., 1994), results from abrogation of the finely balanced interactions of F3 caused by cleavage of the F2-F3 linker. Just as in the case of the W246A mutation, this leads to breakdown of the co-operative pathway of allosteric communication. Interestingly, PARP-1 has been identified recently as one of the most heavily acetylated cellular proteins in response to UV irradiation (Elia et al., 2015), and modification sites map to interaction surfaces, e.g., of F1, F2, and WGR. We propose that the large number and variety of post-translational modifications and interaction partners of PARP-1 (Luo and Kraus, 2012) may allow fine-tuning of its activity in a pathway-specific manner.

PARP-1 Automodification Occurs In *cis*

The structural model of full-length PARP-1 represents the enzyme in a state primed for productive PAR catalysis. It remains an open question what specificity may exist in substrate selection. Proteomics studies have so far not yielded a defined consensus modification site (Zhang et al., 2013), and PARP-1 modifies a wide variety of proteins. We suggest that specificity could depend mainly on substrate recruitment by parts of PARP-1 other than its catalytic domain. The role of these would simply be to bring the target into proximity for modification, as suggested similarly for PAR-ylation by TNKS2 (Guettler et al., 2011). The major target of PAR-ylation is PARP-1 itself, modification of which serves as an important signal for the DNA damage response and releases PARP-1 from DNA (Satoh and Lindahl, 1992; D’Amours et al., 1999; Murai et al., 2012). The monomeric mechanism of SSB-induced activation presented above predicts that such automodification should occur in *cis*, with the same PARP-1 molecule that detects DNA damage also serving as the substrate for automodification.

However, to date, only automodification in *trans* has been reported, based mainly on observed bimolecular reaction kinetics when active calf thymus DNA containing a highly diverse range of DNA ligands was used for activation (Mendoza-Alvarez and Alvarez-Gonzalez, 1993). Indeed, the supposed in *trans* nature of automodification has been invoked widely to support the case for involvement of PARP-1 dimers in catalysis (Pion et al., 2005; Ali et al., 2012). Our in vitro activity assays shown in Figure 6 resolve this paradox. Only when two DNA binding sites are closely adjacent (e.g., a DNA duplex) does automodification occur in *trans*, whereas, on SSB (dumbbell) and DSB (single hairpin) mimics, PARP-1 automodification occurs almost exclusively in *cis*. Selective in *cis* modification likely presents specific regions of PARP-1 for automodification. Indeed, our structural model of SSB-bound full-length PARP-1 together with HXMS data (Dawicki-McKenna et al., 2015) shows that the BRCT-WGR linker, which is known to be automodified, remains flexible and is able to reach the active site of PARP-1. Future proteomic analyses that focus on DNA damage models like those presented in Figure 6, as well as further mechanistic studies of PARP-1’s dynamic nature in particular of the catalytic domain, will likely improve our understanding of how preferential automodification sites regulate biological functions of PARP-1 and other chromatin-associated targets. In *cis* automodification of the activated PARP-1 monomer explains elegantly how the enzyme can rapidly release itself from DNA damage, limiting NAD^+ consumption and allowing DNA repair to proceed.

Conclusions

In this work, we show how the dynamic response of PARP-1’s multi-domain structure to DNA single-strand breaks provides a molecular basis for understanding its central role as a cellular sensor of genotoxic stress. By using NMR spectroscopy in

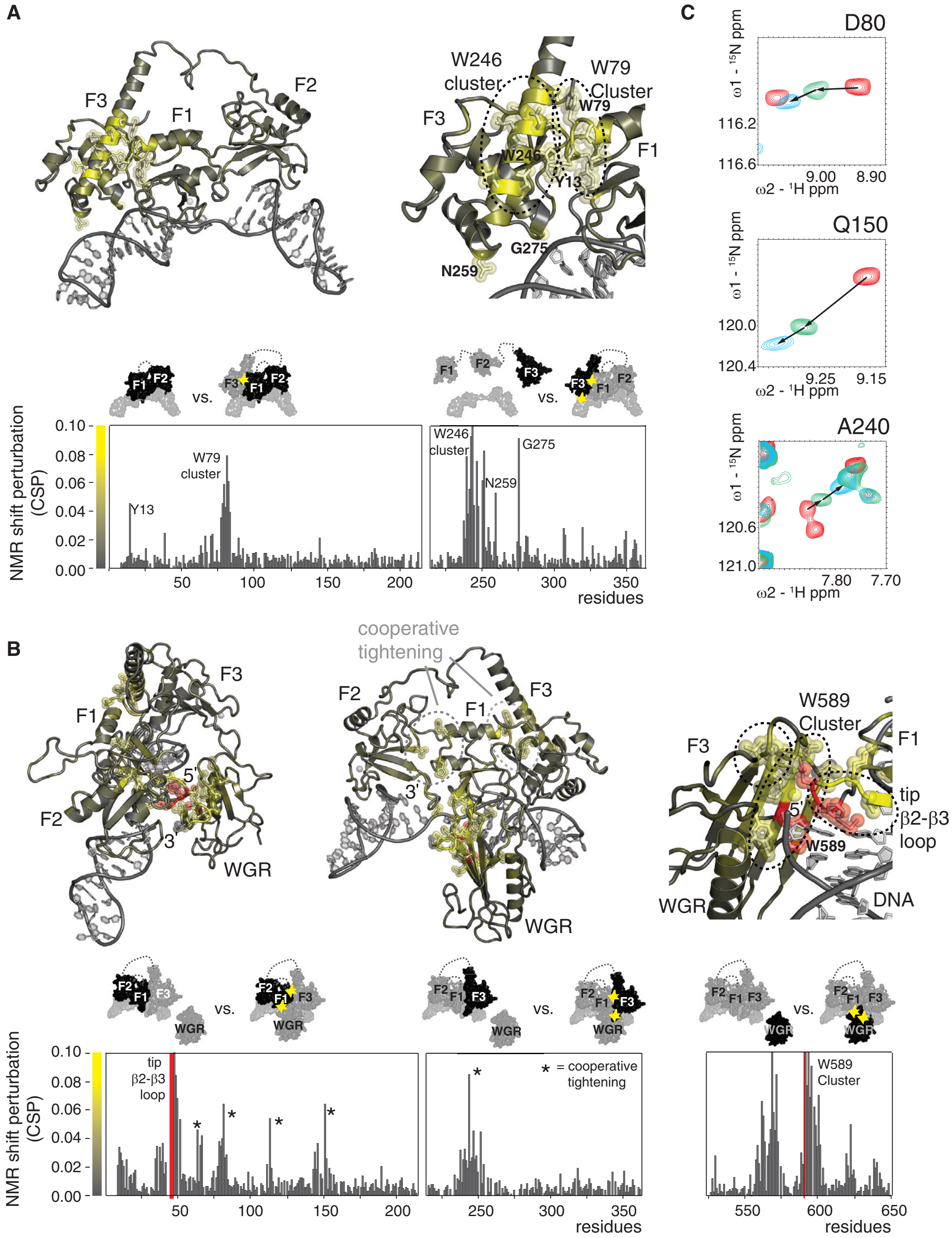

Figure 4. NMR Analysis Uncovers the Multi-Domain Folding Pathway of PARP-1 Domains on an SSB that Underlies DNA-Dependent Switching of Its Activity

(A and B) The 56-kDa F1F2F3-DNA and 71-kDa F1F2F3-WGR-DNA complexes represent intermediate steps toward full assembly. SSB recognition by F1F2 triggers interactions that place F3 (A) and WGR (B) in the correct spatial orientation for the interaction with CAT to trigger its activation. These interactions are evidenced by the amide group CSPs shown in the histograms and mapped on the structures (same co-ordinates as in Figure 3) from gray (CSP = 0) to yellow (CSP = 0.08; CSP > 0.08 is shown in yellow, and side chains are shown for CSP > 0.04). Four residues (red) close to the DNA 5′ terminus became undetectable

(legend continued on next page)

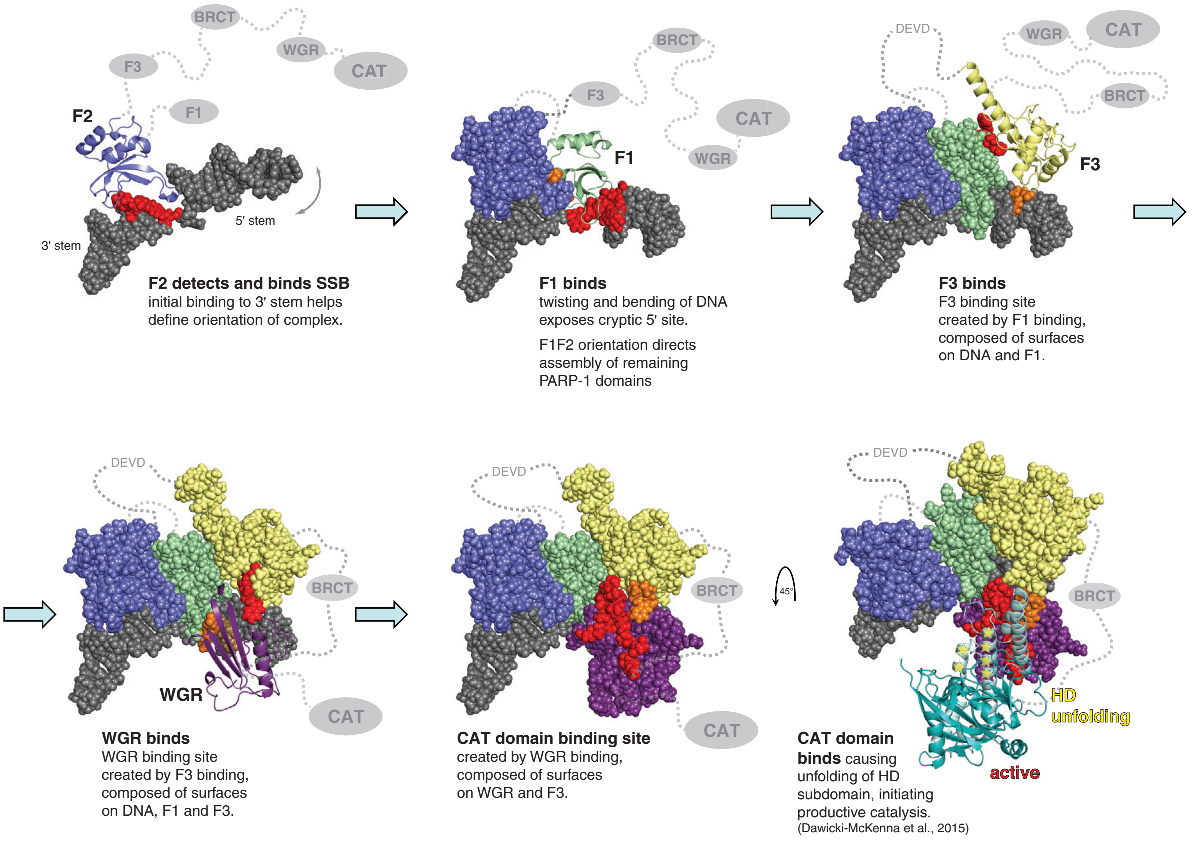

Figure 5. Allosteric Activation Mechanism of PARP-1 by DNA Single-Strand Breaks

In the absence of DNA, PARP-1 domains behave independently, connected by disordered linkers. SSB recognition by F1 and F2 drives multidomain folding, which provides a cooperative switch for activation of the C-terminal catalytic domain (see main text); F3 and WGR are thereby positioned in the correct spatial orientation to trigger their ternary interaction with the catalytic domain. This relieves autoinhibition of the enzyme by causing local unfolding of the HD subdomain (indicated by stars in the figure), which is the subject of the accompanying paper by Dawicki-McKenna (2015). Interfacial residues on different components are colored red and orange. DEVD indicates the caspase cleavage site between F2 and F3. In this figure, we show the pathway as a sequence of discrete steps, but, in reality, they are probably not fully separated. For instance, although F2 likely initiates binding, F1 also co-operates in high-affinity DNA damage recognition. Similarly, although we show F3 binding ahead of WGR, in principle, these events could occur in either order or both. Nevertheless, our data show that elements of the pathway represent intermediate steps because they can occur in isolation. For instance, F1F2 binds an SSB and achieves directional selectivity on its own, and interactions of F3 and WGR occur with the F1F2-DNA complex in the absence of the CAT domain. BRCT, BRCA-1 C terminus.

conjunction with other biophysical and functional techniques, we identify and interrogate successive states on a multi-domain folding pathway of PARP-1 in solution, demonstrating both the structural basis of SSB recognition and the allosteric mechanism through which recruitment to sites of genomic damage is intimately coupled to regulation of PARP-1's catalytic activity for PAR-mediated signaling (summarized in Figure 5).

Many DNA-binding factors recognize their cognate target sites through co-operative homo- and hetero-oligomerization, thereby preventing unwanted activity (e.g., in the absence of interaction partners or at unspecific DNA binding sites). Well known examples include the nuclear hormone receptors (recently reviewed in Helsen et al., 2012). Although DNA-damage-induced dimerization has been proposed previously for PARP-1 (Mendoza-Alvarez and Alvarez-Gonzalez, 1993; Pion et al., 2003; Ali et al., 2012), our data show how the enzyme detects DNA damage as a monomer through co-operative action of its two flexibly linked N-terminal zinc fingers. Based on our findings, we propose a mechanism in which F1 is positioned through binding of F2 via a fly-casting process (Shoemaker et al., 2000). The dynamic features we identify explain how PARP-1 can serve as versatile yet specific first-line sensor of SSBs and related DNA

because of line broadening during WGR titrations. CSPs were measured using sortase-ligated samples to reduce spectral complexity (see the schematics above the histograms; ^{15}N-labeled domains are shown in dark gray, and interaction sites are highlighted in yellow).

(C) In addition to direct interactions, CSPs also show co-operative strengthening throughout the F1F2F3-WGR-DNA complex; addition of WGR reinforces CSPs seen at the F1-F2 and F1-F3 interfaces. Representative examples are shown for each interface (F1-F2, Q150; F1-F3, D80 and A240), superposing spectra of F1F2F3 (red), F1F2F3-DNA (green), and F1F2F3-WGR-DNA (cyan).

See also Figure S5.

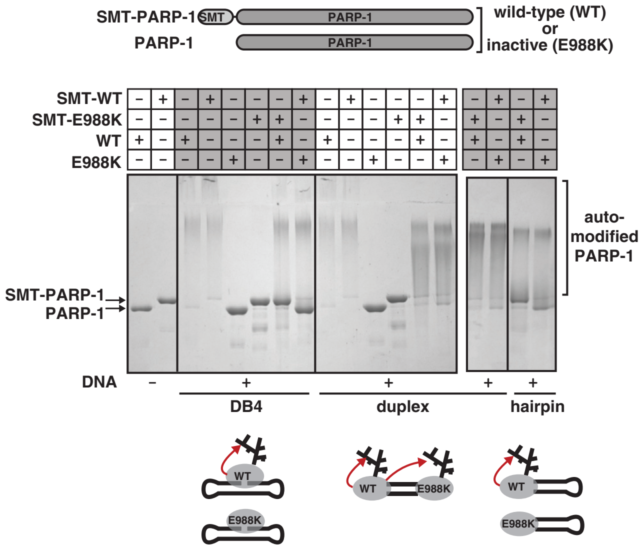

Figure 6. Automodification of PARP-1 on SSBs Occurs In *cis*

Active WT or inactive mutant (E988K) versions were made for both normal and N-terminal SUMO-tagged (SMT) variants of PARP-1 (distinguishable on SDS-PAGE). On mixing active and inactive versions with different tags in the presence of an SSB mimic (DB4; Supplemental Experimental Procedures), only the active WT is automodified (slower-migrating smeared band), indicating selective in *cis* modification of this type of DNA damage. In contrast, both active and inactive PARP-1 molecules are modified in the presence of a DNA duplex, which allows binding on both DNA ends. Blocking one end of the duplex with a hairpin restores selective in *cis* modification.

structures in eukaryotes, efficiently recognizing the many chemically diverse lesions constantly arising from oxidative DNA damage or as intermediates during DNA repair.

Rather than using DNA-binding to bring together separate protein molecules to initiate allosteric activation, PARP-1 instead uses DNA-binding to bring together protein domains within the same molecule. The unique directionality of F1F2 binding establishes a platform for dynamic self-assembly of remaining PARP-1 domains onto the complex. Our data explain how this co-operative, multi-domain folding process acts as a rapid and robust switch for activation, effectively channeling energy from high-affinity DNA binding for activation of PARP-1's catalytic domain, where it triggers productive PAR synthesis via local unfolding of an inhibitory HD subdomain, as observed by Dawicki-McKenna et al. (2015). Overall, this allosteric mechanism ensures that the resulting burst of PAR-mediated signaling and modulation of chromatin structure occurs only at sites of genomic lesions, while automodification in *cis* efficiently removes the monomeric enzyme so that DNA repair and replication can proceed.

Originally, it was suggested that delayed SSB repair (SSBR) caused by PARP-1 inhibition leads to collapsed replication forks and an increase of toxic DSBs (Bryant et al., 2005; Farmer et al., 2005), whereas, more recently, it has been suggested that PARP-1 is also a first-line sensor of stalled forks, providing a bypass for a defective homologous recombination (HR)-mediated replication restart (Bryant et al., 2009). Intriguingly, our data explain how PARP-1 can achieve both of these roles by using the same recognition and activation mechanism, implying that PARP inhibitors may target the same state of the enzyme in more than one pathway. Furthermore, stalling of PARP-1 on DNA lesions has very recently been proposed to form a crucial contribution to synthetic lethality because it may block repair and replication (Murai et al., 2012; Caldecott, 2014; Helleday, 2011; Shen et al., 2015). The SSB-bound conformation of PARP-1 presented here is most likely analogous to such a trapped state of the enzyme, in which high-affinity binding and domain assembly have occurred but subsequent release is prevented by inhibition of PAR automodification. Significantly, recent results have shown that the ability of clinically used PARP inhibitors to kill tumor cells does not correlate with their ability to prevent PAR production but, rather, with their ability to strengthen DNA binding of the inhibited state, which, in turn, must involve allosteric communication between the inhibitor binding site and DNA binding domains of PARP-1 (Murai et al., 2012; Marchand et al., 2014; Mansoorabadi et al., 2014). A detailed knowledge of the cooperative mechanism of PARP-1 assembly on DNA damage, such as provided here, will be essential to understand these effects and may play a key role in the future design of improved inhibitors.

To fulfill its functions, PARP-1 requires a combination of rapid response to an initial stimulus coupled with tight spatio-temporal control of catalytic activity, while also allowing more subtle degrees of regulation. The mechanism of SSB detection and allosteric activation we describe here is ideally suited to meet these demands. Ligand-induced multi-domain folding of domains within a single polypeptide chain represents an extremely efficient way to communicate a signal while maintaining, because of its dynamics, the potential for versatility and modulation so far more often associated with more complex but slower protein interaction networks. It seems likely that other signaling systems may have evolved similar solutions. Allosteric control of dynamic multi-domain proteins has emerged as one of the key molecular mechanisms underlying complex cellular signaling events, and the principles described here may well apply more generally.

EXPERIMENTAL PROCEDURES

Full experimental procedures for cloning, expression, and purification of deuterated, back-labeled protein samples for NMR spectroscopy (including those having different isotope labeling patterns in different domains and prepared using sortase ligation), DNA preparation and purification, isothermal calorimetry, fluorescence anisotropy, analytical ultracentrifugation, activity assays, live-cell imaging, and NMR experiments and assignments are provided in the Supplemental Experimental Procedures. The strategy used here for determining the structure of the F1F2-dumbbell complex was based on a combination of recently published methods and was developed specifically for this project. Because it has not been published previously, in the following we describe the approach. More detailed descriptions are given in the Supplemental Experimental Procedures.

Structure Determination

Analysis of residual dipolar coupling (RDC) and NOE data showed that the internal structures of F1 and F2 are preserved on DNA complex formation and are similar to those determined previously by NMR spectroscopy (Eustermann et al., 2011) and crystallography (Langelier et al., 2011). For both F1 and F2, the match between experimental RDCs and those back-calculated using these crystal structures (PDB: 3ODA for F1 and 3ODC for F2) showed that the structures were equivalent to approximately the degree expected for

X-ray structures in this resolution range (2.6–2.8 Å) (Bax and Grishaev, 2005). The Q values (calculated as Q = root-mean-square [RMS](D_i^{obs}-D_i^{calc})/RMS(D_i^{obs})) for F1 and F2 were 18.9% and 29.9%, respectively (Figure S2n). In addition, the many intra-finger NOEs observed and assigned in spectra of the complex were fully consistent with the previously known structures (Eustermann et al., 2011; Langelier et al., 2011). For the DNA, extensive comparison of free- and bound-state NOE contacts as well as observation of imino NH signals from all the AT and most of the GC base pairs showed that the two DNA stems preserved their conformation in the complex (Figure S3). In the absence of deposited DNA structures for the tetraloop sequences, RNA tetraloops 1MSY (Correll and Swinger, 2003) and 1RNG (Jucker and Pardi, 1995) were used as a basis for modeling. NOE contacts measured for the DNA dumbbell in both free and bound states (Figure S3) are largely consistent with these structures (except that the T35 base is in the anti conformation in the DNA case; Supplemental Experimental Procedures).

NOE contacts, CSP, and TRACT (Lee et al., 2006) data for the F1F2-DNA complex showed clearly that the dumbbell accommodates simultaneous binding of both fingers, with F2 binding the 3′ DNA stem and F1 the 5′ stem. Nine intermolecular NOE contacts were assigned, linking the 3′-terminal nucleotide G45 to residues Leu151 and Ile154 of F2 and five others linking C22 on the 5′ stem to residue Val48 of F1 (Figure 1F; Figure S2, Table S2). Additional evidence for this arrangement came from comparisons of amide group TROSY spectra of complexes of F1F2 with different DNA dumbbells. Changes in the DNA 3′ stem sequence caused CSPs almost exclusively in signals from F2, whereas changes in the 5′ stem sequence caused CSPs almost exclusively in signals from F1 (Figure S1).

The NOE and CSP data further established that the binding mode of F1 to the 5′ DNA stem and of F2 to the 3′ DNA stem in solution were both highly similar to that seen in both crystal structures of the individual PARP-1 F1 and F2 fingers, each bound to similar sites on DNA blunt ends (PDB: 3ODA and 3ODC, respectively) (Langelier et al., 2011). In both cases, the observed intermolecular NOE contacts described above exactly parallel contacts seen in the crystal structures (Figure 1F; Table S2). For F1 to bind to the DNA 5′ stem in the same way as F2 binds to the 3′ stem requires that the continuous DNA strand linking the stems must adopt a conformation very far from B-form if it is to avoid steric clashes with the protein, and a clear sign confirming it does so was provided by three intermolecular NOE contacts connecting Val48 to the linking nucleotide, T23. Further evidence for the overall interaction mode of fingers with stems comes from CSPs observed both for protein and DNA signals on complexation that parallel the interfaces seen in the crystal structures. These include large upfield shifts for both Phe44 and G1, strongly suggesting that they are mutually stacked, as well as CSPs on the DNA consistent with insertion of Arg18 of F1 and Arg122 of F2 into the DNA minor groove (Figures 2A and 2B).

To test whether F1 and F2 form a direct F1-F2 interaction in the complex, which could play an important role in determining the mutual orientation of the fingers, sortase-ligated constructs having different labeling in each finger were used to allow selective measurement of NOE contacts between F1 and F2 freed from overlap with other signals (Figure S2). This revealed a network of interdomain NOE contacts linking Pro149, Met153, and Val144 on F2 to Leu8, Ile37, Val39, and Val60 on F1 (Figure 1F; Figure S2; Table S2).

These data were used together to calculate a structure of the complex using a hybrid approach combining information from X-ray crystallography and NMR spectroscopy, using the program XPLOR-NIH (Schwieters et al., 2003). Because the NMR data clearly established that F1 and F2 bind to the 5′ and 3′ stems of the DNA dumbbell, respectively, and that the internal structures of the fingers, DNA stems, and the interfaces between each finger and its associated stem were very similar to those in crystal structures 3ODA and 3ODC (although in a different overall context), the calculations were restrained to reproduce these features by using non-crystallographic symmetry (NCS) terms in the force field (relative to fixed copies of starting structures in which these features were present; Supplemental Experimental Procedures). Fitting RDC data for both fingers to a single optimized alignment tensor using the implicit Saupe tensor alignment constraint (ISAC) protocol of Sass et al. (2001) defined the orientation of the fingers with respect to one another, while the NOE-derived inter-finger distance restraints defined the structure of their mutual interface, and intermolecular NOE-derived restraints helped define the DNA conformation at the gap. Regions of the complex for which there was no a priori information (e.g., the DNA linker joining the two stems), where the two fingers make contact, or that were shown to be dynamic by NMR relaxation experiments (e.g., the N- and C-terminal tails and the interfinger linker; Figure 1) were treated as being flexible. This was achieved in different ways in each case. The dihedrals of the DNA linker were randomized in the starting structures, for atoms of the F1-F2 interface the NCS force constant was reduced to a low value, and atoms of the protein linker were added in a second calculation step only after the relative positions of the fingers had been defined. Using a simulated annealing protocol similar to a conventional NMR structure determination, we were thus able to combine all of this information and determine an ensemble of structures consistent with the data, the result of which is shown in Figure 1E, with statistics summarized in Table 1 (see Supplemental Experimental Procedures for further description). The backbone precision across the ordered regions is approximately 0.34 Å, implying that the NMR-based constraints define the overall architecture quite closely. As with essentially any solution structure, the calculated ensemble spread reflects the precision with which the mean structure has been established from averaged data. In general, it does not necessarily provide a measure of the true spread in solution. However, the fact that we observed interdomain NOE contacts with reasonable strength shows that there must be a significant population of structures in solution in which these contacts are present, and cross-validation with SAXS measurements confirms independently that the overall shape of the complex is accurate (Figure 1H).

ACCESSION NUMBERS

The co-ordinates for the ensemble of 78 structures of the F1F2-DNA dumbbell complex reported in this paper have been deposited in the PDB under ID code PDB: 2n8a. Chemical shift assignments are reported as follows: F1F2-DNA complex, BMRB: 25888; F1F2 free, BMRB: 25889; DNA free, BMRB: 25890; F1F2F3-DNA complex, BMRB: 25891; F1F2F3 free, BMRB: 25892; F3 free, BMRB: 25893; F1F2F3-WGR-DNA complex, BMRB: 25894; WGR free, BMRB: 25895.

SUPPLEMENTAL INFORMATION

Supplemental Information includes Supplemental Experimental Procedures, five figures, and two tables and can be found with this article online at http://dx.doi.org/10.1016/j.molcel.2015.10.032.

AUTHOR CONTRIBUTIONS

D.N. and S.E. designed the project. S.E., W.F.W., and L.E.E. cloned, expressed, and purified proteins for NMR. S.E., W.F.W., and J.C.Y. designed, collected, and processed NMR experiments. S.E., W.F.W., J.C.Y., and D.N. analyzed and assigned NMR data. D.N. and S.E. designed and carried out structural calculations and analyses. S.E. and W.F.W. carried out and analyzed ITC experiments and SAXS experiments. M.F.L. and J.M.P. designed and performed the in *cis* modification and AUC experiments. M.F.L. expressed and purified proteins and performed biochemical analysis of full-length PARP-1 and mutants. A.A.R. performed live-cell imaging experiments. S.E., M.F.L., J.M.P., and D.N. wrote the manuscript.

ACKNOWLEDGMENTS

We thank Chris Johnson for ITC and Donna Williams and Matthew Watson for DNA synthesis; Fuyuhiko Inagaki for a generous gift of the plasmid for the wild-type *Staph Aureus* Δ59 sortase enzyme; Jakob Nielsen for help with the free DNA assignment; Henrik Wegener for help with the RDC analysis; Kiyoshi Nagai, and Karl-Peter Hopfner for helpful discussions and support; and Julianne Brummer, Paul Cole, Arek Kulczyk, and Tennie Videler for earlier contributions to parts of the project not described in this paper. We thank Gregor Witte and the staff at beamline EMBL/DESY P12 for help and support during SAXS data collection. We also thank the staff at the SIBYLS 12.3.1 beamline, where initial

SAXS data (not reported in the paper) were recorded under their mail-in program, DOE grant DE-AC02-05CH11231. We acknowledge the support of the Medical Research Council (Grant U105178934 to D.N.), Worldwide Cancer Research (Grant 10-0193 to D.N. and S.E.), Boehringer Ingelheim Fonds (studentship to S.E.), EMBO (long-term fellowship to S.E.) and the Shared Resources of the Sydney Kimmel Cancer Center at Thomas Jefferson University (Grant P30CA56036) and the NIH (Grant GM087282) (to J.M.P.).

Received: August 17, 2015
Revised: September 26, 2015
Accepted: October 16, 2015
Published: November 25, 2015

REFERENCES

Ali, A.A., Timinszky, G., Arribas-Bosacoma, R., Kozlowski, M., Hassa, P.O., Hassler, M., Ladurner, A.G., Pearl, L.H., and Oliver, A.W. (2012). The zinc-finger domains of PARP1 cooperate to recognize DNA strand breaks. Nat. Struct. Mol. Biol. *19*, 685–692.

Bax, A., and Grishaev, A. (2005). Weak alignment NMR: a hawk-eyed view of biomolecular structure. Curr. Opin. Struct. Biol. *15*, 563–570.

Bryant, H.E., Schultz, N., Thomas, H.D., Parker, K.M., Flower, D., Lopez, E., Kyle, S., Meuth, M., Curtin, N.J., and Helleday, T. (2005). Specific killing of BRCA2-deficient tumours with inhibitors of poly(ADP-ribose) polymerase. Nature *434*, 913–917.

Bryant, H.E., Petermann, E., Schultz, N., Jemth, A.S., Loseva, O., Issaeva, N., Johansson, F., Fernandez, S., McGlynn, P., and Helleday, T. (2009). PARP is activated at stalled forks to mediate Mre11-dependent replication restart and recombination. EMBO J. *28*, 2601–2615.

Caldecott, K.W. (2008). Single-strand break repair and genetic disease. Nat. Rev. Genet. *9*, 619–631.

Caldecott, K.W. (2014). Protein ADP-ribosylation and the cellular response to DNA strand breaks. DNA Repair (Amst.) *19*, 108–113.

Chao, L.H., Stratton, M.M., Lee, I.H., Rosenberg, O.S., Levitz, J., Mandell, D.J., Kortemme, T., Groves, J.T., Schulman, H., and Kuriyan, J. (2011). A mechanism for tunable autoinhibition in the structure of a human Ca2+/calmodulin- dependent kinase II holoenzyme. Cell *146*, 732–745.

Chou, D.M., Adamson, B., Dephoure, N.E., Tan, X., Nottke, A.C., Hurov, K.E., Gygi, S.P., Colaiácovo, M.P., and Elledge, S.J. (2010). A chromatin localization screen reveals poly (ADP ribose)-regulated recruitment of the repressive polycomb and NuRD complexes to sites of DNA damage. Proc. Natl. Acad. Sci. USA *107*, 18475–18480.

Correll, C.C., and Swinger, K. (2003). Common and distinctive features of GNRA tetraloops based on a GUAA tetraloop structure at 1.4 A resolution. RNA *9*, 355–363.

D'Amours, D., Desnoyers, S., D'Silva, I., and Poirier, G.G. (1999). Poly(ADP-ribosyl)ation reactions in the regulation of nuclear functions. Biochem. J. *342*, 249–268.

Dawicki-McKenna, J.M., Langelier, M.F., DeNizio, J.E., Riccio, A.A., Cao, C.D., Karch, K.R., McCauley, M., Steffen, J.D., Black, B.E., and Pascal, J.M. (2015). PARP-1 activation requires local unfolding of an autoinhibitory domain. Mol. Cell *60*, this issue, 755–768.

de Murcia, G., and Ménissier de Murcia, J. (1994). Poly(ADP-ribose) polymerase: a molecular nick-sensor. Trends Biochem. Sci. *19*, 172–176.

de Murcia, G., Schreiber, V., Molinete, M., Saulier, B., Poch, O., Masson, M., Niedergang, C., and Ménissier de Murcia, J. (1994). Structure and function of poly(ADP-ribose) polymerase. Mol. Cell. Biochem. *138*, 15–24.

Durkacz, B.W., Omidiji, O., Gray, D.A., and Shall, S. (1980). (ADP-ribose)n participates in DNA excision repair. Nature *283*, 593–596.

Elia, A.E., Boardman, A.P., Wang, D.C., Huttlin, E.L., Everley, R.A., Dephoure, N., Zhou, C., Koren, I., Gygi, S.P., and Elledge, S.J. (2015). Quantitative Proteomic Atlas of Ubiquitination and Acetylation in the DNA Damage Response. Mol. Cell *59*, 867–881.

Eustermann, S., Videler, H., Yang, J.C., Cole, P.T., Gruszka, D., Veprintsev, D., and Neuhaus, D. (2011). The DNA-binding domain of human PARP-1 interacts with DNA single-strand breaks as a monomer through its second zinc finger. J. Mol. Biol. *407*, 149–170.

Farmer, H., McCabe, N., Lord, C.J., Tutt, A.N., Johnson, D.A., Richardson, T.B., Santarosa, M., Dillon, K.J., Hickson, I., Knights, C., et al. (2005). Targeting the DNA repair defect in BRCA mutant cells as a therapeutic strategy. Nature *434*, 917–921.

Fernández, C., and Wider, G. (2003). TROSY in NMR studies of the structure and function of large biological macromolecules. Curr. Opin. Struct. Biol. *13*, 570–580.

Fouquerel, E., Goellner, E.M., Yu, Z., Gagné, J.P., Barbi de Moura, M., Feinstein, T., Wheeler, D., Redpath, P., Li, J., Romero, G., et al. (2014). ARTD1/PARP1 negatively regulates glycolysis by inhibiting hexokinase 1 independent of NAD+ depletion. Cell Rep. *8*, 1819–1831.

Göbl, C., Madl, T., Simon, B., and Sattler, M. (2014). NMR approaches for structural analysis of multidomain proteins and complexes in solution. Prog. Nucl. Magn. Reson. Spectrosc. *80*, 26–63.

Guettler, S., LaRose, J., Petsalaki, E., Gish, G., Scotter, A., Pawson, T., Rottapel, R., and Sicheri, F. (2011). Structural basis and sequence rules for substrate recognition by Tankyrase explain the basis for cherubism disease. Cell *147*, 1340–1354.

Helleday, T. (2011). The underlying mechanism for the PARP and BRCA synthetic lethality: clearing up the misunderstandings. Mol. Oncol. *5*, 387–393.

Helsen, C., Kerkhofs, S., Clinckemalie, L., Spans, L., Laurent, M., Boonen, S., Vanderschueren, D., and Claessens, F. (2012). Structural basis for nuclear hormone receptor DNA binding. Mol. Cell. Endocrinol. *348*, 411–417.

Hunter, C.A., and Anderson, H.L. (2009). What is cooperativity? Angew. Chem. Int. Ed. Engl. *48*, 7488–7499.

Jucker, F.M., and Pardi, A. (1995). Solution structure of the CUUG hairpin loop: a novel RNA tetraloop motif. Biochemistry *34*, 14416–14427.

Khodyreva, S.N., Prasad, R., Ilina, E.S., Sukhanova, M.V., Kutuzov, M.M., Liu, Y., Hou, E.W., Wilson, S.H., and Lavrik, O.I. (2010). Apurinic/apyrimidinic (AP) site recognition by the 5′-dRP/AP lyase in poly(ADP-ribose) polymerase-1 (PARP-1). Proc. Natl. Acad. Sci. USA *107*, 22090–22095.

Kim, M.Y., Mauro, S., Gévry, N., Lis, J.T., and Kraus, W.L. (2004). NAD^+-dependent modulation of chromatin structure and transcription by nucleosome binding properties of PARP-1. Cell *119*, 803–814.

Kobashigawa, Y., Kumeta, H., Ogura, K., and Inagaki, F. (2009). Attachment of an NMR-invisible solubility enhancement tag using a sortase-mediated protein ligation method. J. Biomol. NMR *43*, 145–150.

Krishnakumar, R., and Kraus, W.L. (2010). The PARP side of the nucleus: molecular actions, physiological outcomes, and clinical targets. Mol. Cell *39*, 8–24.

Lamers, M.H., Perrakis, A., Enzlin, J.H., Winterwerp, H.H.K., de Wind, N., and Sixma, T.K. (2000). The crystal structure of DNA mismatch repair protein MutS binding to a G x T mismatch. Nature *407*, 711–717.

Langelier, M.-F., Planck, J.L., Roy, S., and Pascal, J.M. (2011). Crystal structures of poly(ADP-ribose) polymerase-1 (PARP-1) zinc fingers bound to DNA: structural and functional insights into DNA-dependent PARP-1 activity. J. Biol. Chem. *286*, 10690–10701.

Langelier, M.F., Planck, J.L., Roy, S., and Pascal, J.M. (2012). Structural basis for DNA damage-dependent poly(ADP-ribosyl)ation by human PARP-1. Science *336*, 728–732.

Lazebnik, Y.A., Kaufmann, S.H., Desnoyers, S., Poirier, G.G., and Earnshaw, W.C. (1994). Cleavage of poly(ADP-ribose) polymerase by a proteinase with properties like ICE. Nature *371*, 346–347.

Le Cam, E., Fack, F., Ménissier-de Murcia, J., Cognet, J.A., Barbin, A., Sarantoglou, V., Révet, B., Delain, E., and de Murcia, G. (1994). Conformational analysis of a 139 base-pair DNA fragment containing a single-stranded break and its interaction with human poly(ADP-ribose) polymerase. J. Mol. Biol. *235*, 1062–1071.

Lee, D., Hilty, C., Wider, G., and Wuthrich, K. (2006). Effective rotational correlation times of proteins from NMR relaxation interference. J. Magn. Reson. *178*, 72–76.

Lilyestrom, W., van der Woerd, M.J., Clark, N., and Luger, K. (2010). Structural and biophysical studies of human PARP-1 in complex with damaged DNA. J. Mol. Biol. *395*, 983–994.

Lord, C.J., and Ashworth, A. (2013). Mechanisms of resistance to therapies targeting BRCA-mutant cancers. Nat. Med. *19*, 1381–1388.

Luo, X., and Kraus, W.L. (2012). On PAR with PARP: cellular stress signaling through poly(ADP-ribose) and PARP-1. Genes Dev. *26*, 417–432.

Mackereth, C.D., Madl, T., Bonnal, S., Simon, B., Zanier, K., Gasch, A., Rybin, V., Valcárcel, J., and Sattler, M. (2011). Multi-domain conformational selection underlies pre-mRNA splicing regulation by U2AF. Nature *475*, 408–411.

Mansoorabadi, S.O., Wu, M., Tao, Z., Gao, P., Pingali, S.V., Guo, L., and Liu, H.W. (2014). Conformational activation of poly(ADP-ribose) polymerase-1 upon DNA binding revealed by small-angle X-ray scattering. Biochemistry *53*, 1779–1788.

Marchand, J.R., Carotti, A., Passeri, D., Filipponi, P., Liscio, P., Camaioni, E., Pellicciari, R., Gioiello, A., and Macchiarulo, A. (2014). Investigating the allosteric reverse signalling of PARP inhibitors with microsecond molecular dynamic simulations and fluorescence anisotropy. Biochim. Biophys. Acta *1844*, 1765–1772.

Mendes-Pereira, A.M., Martin, S.A., Brough, R., McCarthy, A., Taylor, J.R., Kim, J.S., Waldman, T., Lord, C.J., and Ashworth, A. (2009). Synthetic lethal targeting of PTEN mutant cells with PARP inhibitors. EMBO Mol. Med. *1*, 315–322.

Mendoza-Alvarez, H., and Alvarez-Gonzalez, R. (1993). Poly(ADP-ribose) polymerase is a catalytic dimer and the automodification reaction is intermolecular. J. Biol. Chem. *268*, 22575–22580.

Min, J.H., and Pavletich, N.P. (2007). Recognition of DNA damage by the Rad4 nucleotide excision repair protein. Nature *449*, 570–575.

Molinete, M., Vermeulen, W., Bürkle, A., Ménissier-de Murcia, J., Küpper, J.H., Hoeijmakers, J.H., and de Murcia, G. (1993). Overproduction of the poly(ADP-ribose) polymerase DNA-binding domain blocks alkylation-induced DNA repair synthesis in mammalian cells. EMBO J. *12*, 2109–2117.

Moreno-Herrero, F., de Jager, M., Dekker, N.H., Kanaar, R., Wyman, C., and Dekker, C. (2005). Mesoscale conformational changes in the DNA-repair complex Rad50/Mre11/Nbs1 upon binding DNA. Nature *437*, 440–443.

Murai, J., Huang, S.Y., Das, B.B., Renaud, A., Zhang, Y., Doroshow, J.H., Ji, J., Takeda, S., and Pommier, Y. (2012). Trapping of PARP1 and PARP2 by Clinical PARP Inhibitors. Cancer Res. *72*, 5588–5599.

Pion, E., Bombarda, E., Stiegler, P., Ullmann, G.M., Mély, Y., de Murcia, G., and Gérard, D. (2003). Poly(ADP-ribose) polymerase-1 dimerizes at a 5′ recessed DNA end in vitro: a fluorescence study. Biochemistry *42*, 12409–12417.

Pion, E., Ullmann, G.M., Amé, J.C., Gérard, D., de Murcia, G., and Bombarda, E. (2005). DNA-induced dimerization of poly(ADP-ribose) polymerase-1 triggers its activation. Biochemistry *44*, 14670–14681.

Polo, S.E., and Jackson, S.P. (2011). Dynamics of DNA damage response proteins at DNA breaks: a focus on protein modifications. Genes Dev. *25*, 409–433.

Sass, H.J., Musco, G., Stahl, S.J., Wingfield, P.T., and Grzesiek, S. (2001). An easy way to include weak alignment constraints into NMR structure calculations. J. Biomol. NMR *21*, 275–280.

Satoh, M.S., and Lindahl, T. (1992). Role of poly(ADP-ribose) formation in DNA repair. Nature *356*, 356–358.

Schreiber, V., Dantzer, F., Ame, J.C., and de Murcia, G. (2006). Poly(ADP-ribose): novel functions for an old molecule. Nat. Rev. Mol. Cell Biol. *7*, 517–528.

Schwieters, C.D., Kuszewski, J.J., Tjandra, N., and Clore, G.M. (2003). The Xplor-NIH NMR molecular structure determination package. J. Magn. Reson. *160*, 65–73.

Shen, Y., Aoyagi-Scharber, M., and Wang, B. (2015). Trapping Poly(ADP-Ribose) Polymerase. J. Pharmacol. Exp. Ther. *353*, 446–457.

Shoemaker, B.A., Portman, J.J., and Wolynes, P.G. (2000). Speeding molecular recognition by using the folding funnel: the fly-casting mechanism. Proc. Natl. Acad. Sci. USA *97*, 8868–8873.

Sonnenblick, A., de Azambuja, E., Azim, H.A., Jr., and Piccart, M. (2015). An update on PARP inhibitors–moving to the adjuvant setting. Nat. Rev. Clin. Oncol. *12*, 27–41.

Zhang, Y., Wang, J., Ding, M., and Yu, Y. (2013). Site-specific characterization of the Asp- and Glu-ADP-ribosylated proteome. Nat. Methods *10*, 981–984.

Molecular Cell, Volume *60*

Supplemental Information

Structural Basis of Detection and Signaling of DNA Single-Strand Breaks by Human PARP-1

Sebastian Eustermann, Wing-Fung Wu, Marie-France Langelier, Ji-Chun Yang, Laura E. Easton, Amanda A. Riccio, John M. Pascal, and David Neuhaus

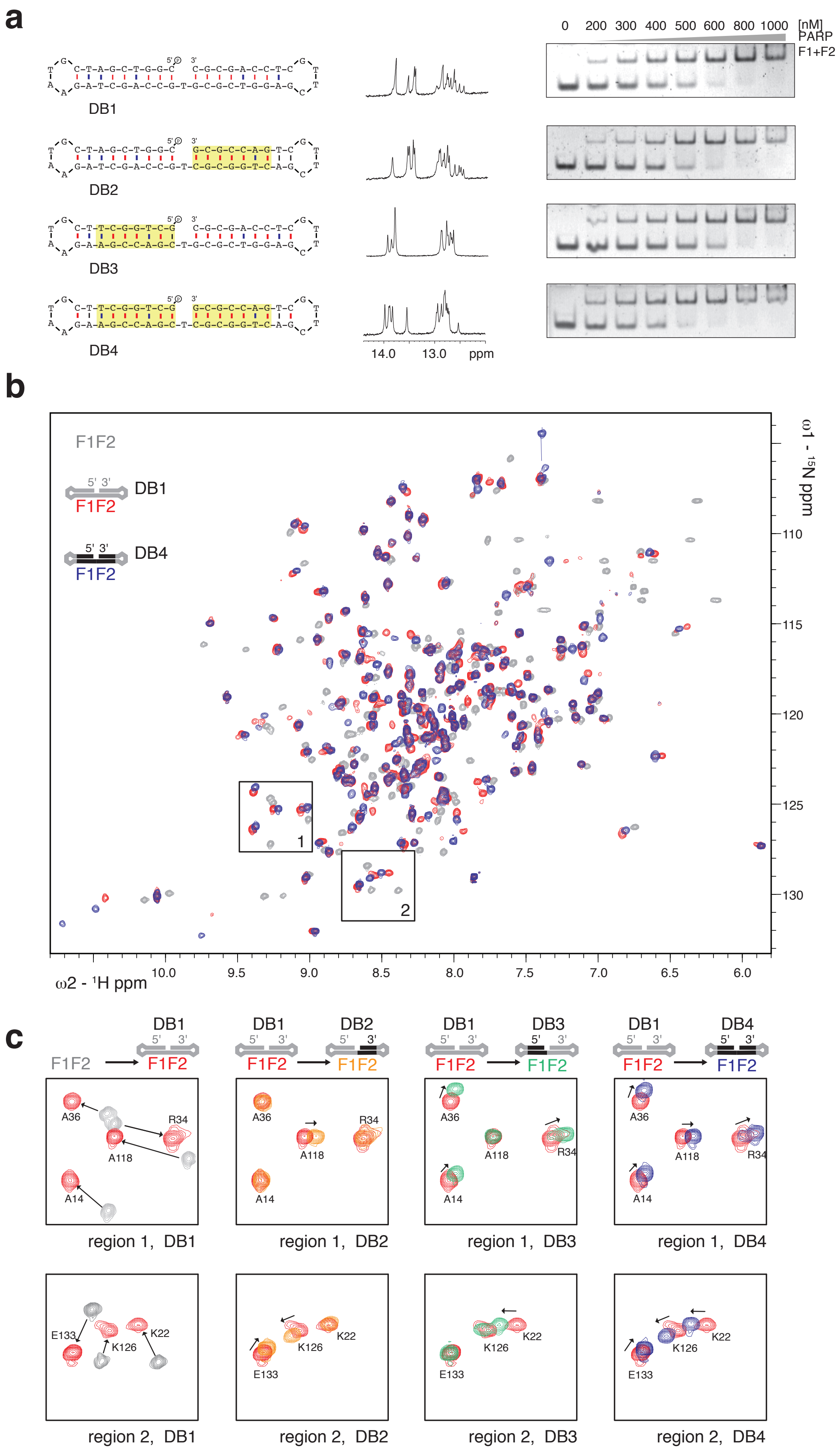

a
DB1
DB2
DB3
DB4
14.0
13.0
ppm
0 200 300 400 500 600 800 1000 [nM]
PARP
F1+F2
b
F1F2
DB1
F1F2
DB4
F1F2
ω1 - 15N ppm
ω2 - 1H ppm
1
2
c
region 1, DB1
region 1, DB2
region 1, DB3
region 1, DB4
region 2, DB1
region 2, DB2
region 2, DB3
region 2, DB4

Figure S1, Related to Figure 1:

Comparison of CSPs induced by different DNA dumbbell sequences reveals the orientation of F1F2 on the SSB ligand. a) DNA dumbbell sequences. DB1 was used in Eustermann *et al.* (2011), DB2 differs only in the 3' stem, DB3 differs only in the 5' stem, while DB4 (as used elsewhere in the present work, since complex F1F2-DB4 gave slightly higher quality spectra than did F1F2- DB1) combines both sets of variations. In each case, the imino region of the ^{1}H 1D spectrum confirms that basepairing in the stems is maintained, and an EMSA assay confirms that F1F2 binding occurs with comparable affinity. b) [^{15}N,^{1}H] correlation spectra of F1F2 in the free state (gray; HSQC), bound to DNA dumbbell ligand DB1 (red; TROSY) or bound to DNA dumbbell ligand DB4 (blue; TROSY); TROSY spectra were displaced by $^{1}J(^{15}N,^{1}H)/2$ in each dimension to make their peak positions comparable to those in the HSQC spectra. c) Expansions of regions 1 and 2 (defined in panel b) for complexes with dumbbells DB1-DB4; protein signal assignments are shown on the plots. The first box in each series shows CSPs of free protein signals on binding dumbbell DB1, while subsequent boxes compare complexes with different DNA dumbbell sequences. When the sequence of the 3' stem of the DNA dumbbell is varied, it is exclusively signals from F2 that are perturbed (A118, K126 and E133), whereas when the sequence of the 5' stem is varied only signals from F1 are perturbed (A14, K22, R34 and A36). When both are varied, protein perturbations are cumulative.

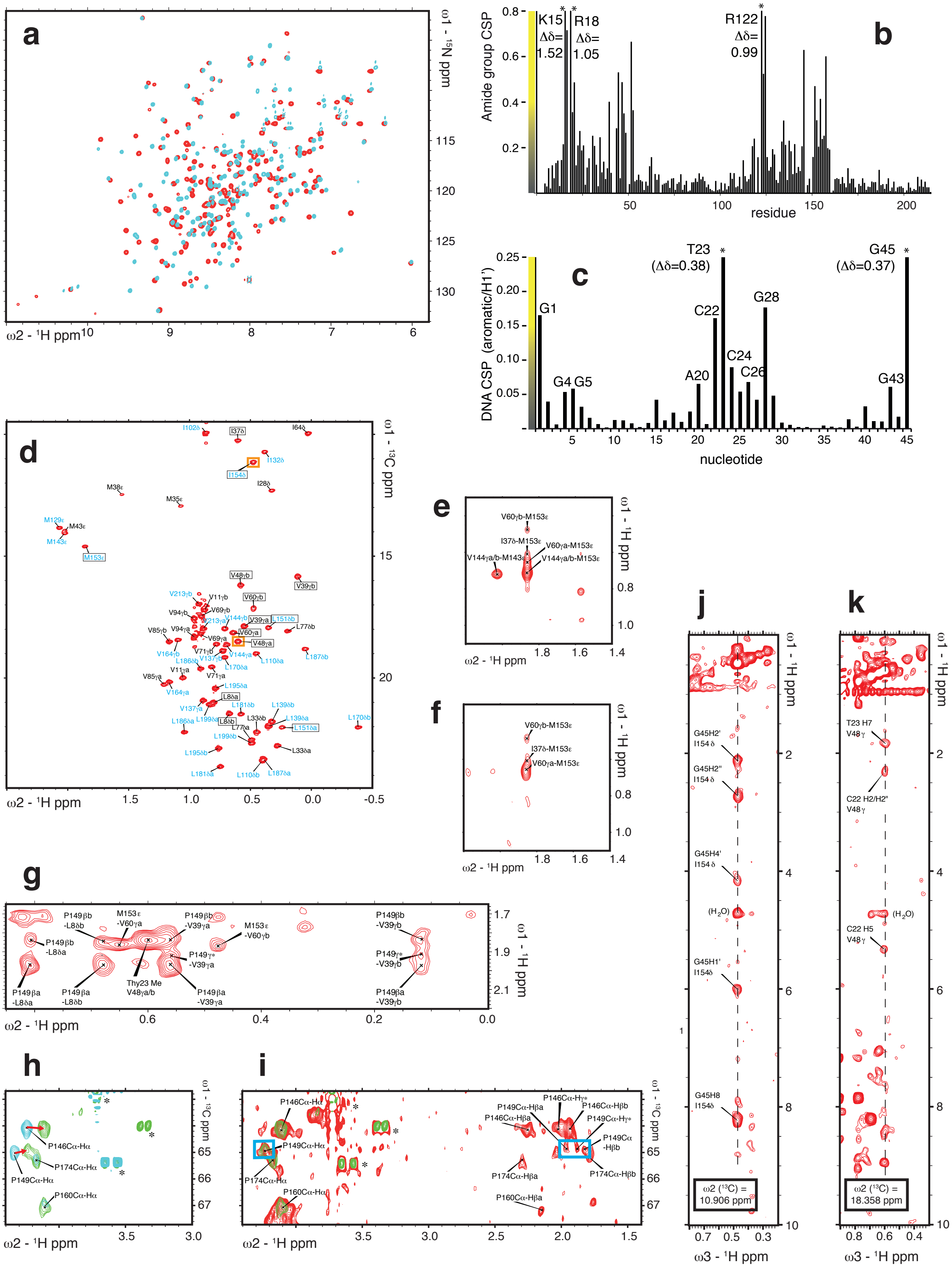

a
ω1 - ¹⁵N ppm
ω2 - ¹H ppm
b
Amide group CSP
residue
K15 Δδ= 1.52
R18 Δδ= 1.05
R122 Δδ= 0.99
c
DNA CSP (aromatic/H1')
nucleotide
T23 (Δδ=0.38)
G45 (Δδ=0.37)
G1
G4 G5
A20
C22
C24
C26
G28
G43
d
ω1 - ¹³C ppm
ω2 - ¹H ppm
e
V60γb-M153ε
I37δ-M153ε
V60γa-M153ε
V144γa/b-M143ε
V144γa/b-M153ε
ω1 - ¹H ppm
ω2 - ¹H ppm
f
V60γb-M153ε
I37δ-M153ε
V60γa-M153ε
g
ω1 - ¹H ppm
ω2 - ¹H ppm
h
P146Cα-Hα
P174Cα-Hα
P149Cα-Hα
P160Cα-Hα
ω1 - ¹³C ppm
ω2 - ¹H ppm
i
ω1 - ¹³C ppm
ω2 - ¹H ppm
j
ω1 - ¹H ppm
ω3 - ¹H ppm
ω2 (¹³C) = 10.906 ppm
k
ω1 - ¹H ppm
ω3 - ¹H ppm
ω2 (¹³C) = 18.358 ppm

Figure S2, Related to Figure 1:

Representative NMR data used during the signal assignment and structure determination of the F1F2-DNA dumbbell complex; all labeling schemes referred to below are defined in Supplemental Experimental Procedures. a) Overlay of [^{15}N,^{1}H] HSQC spectra of F1F2 in the free (cyan) and DNA-bound (red) states (for clarity, assignments are not marked), recorded at 800 MHz and 37°C. These data were used to calculate the CSP values shown in b), which in turn were used to generate the color ramp mapped on the molecular structure in Fig. 2; the color ramp runs from gray ($\Delta\delta = 0$) to yellow ($\Delta\delta = 0.4$; $\Delta\delta > 0.4$ shown yellow). c) Corresponding CSP values measured for the DNA, using the formula $((\Delta\delta(H1'))^2 + (\Delta\delta(H^{arom}))^2)^{1/2}$ (where H^{arom} is H8 for Ade, H6 for Cyt, H8 for Gua and H6 for Thy); in this case the color ramp runs from gray ($\Delta\delta = 0$) to yellow ($\Delta\delta = 0.15$; $\Delta\delta > 0.15$ shown yellow). d) Methyl region of a 2D [^{13}C,^{1}H] HMQC spectrum of the F1F2-DNA dumbbell complex; only methyl signals of Ile, Leu, Val and Met residues appear since just these are “reverse-labeled” in an otherwise deuterated background (labeling scheme IVa). Here methyls appear from both finger F1 (assignments labeled in black; all methyls assigned) and from finger F2 (assignments labeled in cyan; only Val202 unassigned), but these contributions were separated in other samples produced using sortase ligation, in which residues of only one finger were reverse-labeled. Methyls for which interdomain or intermolecular NOE interactions were used in the structure calculations appear boxed in black. Panels e) and f) show 2D NOESY experiments designed to confirm assignments of cross peaks linking Val60 and Met153. In e), the sample is reverse-labeled (Ile, Leu, Val and Met residues) in both fingers F1 and F2 (labeling scheme V), and the spectrum includes crosspeaks at positions corresponding to both intradomain (V144/M143) and interdomain (V60/M153 and I37/M153) interactions. Repeating the experiment using a sortase ligated sample in which only finger F2 was reverse-labeled, in this case only with Met (and Arg) residues, while finger F1 uniformly contained ^{1}H and ^{12}C (labeling scheme VIII) eliminates the crosspeaks due to Val/Met interactions within finger F2, leaving only the interdomain crosspeaks to Val60. These assignments (and others) were all confirmed by observation of corresponding crosspeaks in both 3D [^{1}H,^{13}C,^{1}H] NOESY-HMQC and 3D [^{13}C,^{13}C,^{1}H] HMQC-NOESY-HMQC spectra (Table S2). g) Part of a filtered 2D [^{1}H, ^{1}H] NOESY spectrum of F1F2-DNA dumbbell complex. Here F1F2 was produced by sortase ligation such that finger F1 was reverse-labeled ([^{13}C, ^{1}H] methyl groups for Ile, Leu, Val and Met in a deuterated background), while finger F2 uniformly contained ^{1}H and ^{12}C (labeling scheme VII), and in this filtered NOESY experiment only ^{13}C-bound protons were selected in $\omega 2$, while no selection was made in $\omega 1$. NOE crosspeaks in this spectrum therefore arise from interactions between ^{13}C-labeled methyls in finger F1 (appearing with shifts in $\omega 2$) and protons in either finger F2 or the DNA (appearing with shifts in $\omega 1$). The region shown contains, *inter alia*, crosspeaks linking methyls of Val39 and Leu8 to multiplets at 1.8-2.0 ppm assigned to Pro149. Panels h) and i) show spectra that were used to confirm the assignment of these multiplets to the Hβ and Hγ protons of Pro149 (asterisks indicate small molecule impurities). Back-labeling the prolines of (only) finger F2 with [^{13}C,^{1}H] in a deuterated context (labeling scheme XI) allowed unambiguous identification of the bound-state Hα and Cα signals of Pro149 (by comparing

free-state (green) and bound-state (cyan) HSQC spectra (panel h) in combination with knowledge of Cα assignments from backbone experiments in both free and bound states). This assignment was then extended to the Hβ and Hγ bound-state signals using a 2D [^{13}C,^{1}H] HSQC-NOESY spectrum of the complex (panel i); the Pro149 signals are shown boxed, and correspond closely to those seen for Pro149 in panel g). Panels j) and k) show parts of (ω1, ω3) planes from a 3D [^{1}H,^{13}C,^{1}H] NOESY-HMQC spectrum of the same sample as in panel d), showing intermolecular NOE cross peaks linking j) Ile154δ to protons of nucleotide G45, and k) both γ methyls of Val48 (too close together to be resolved in this spectrum) to protons of nucleotide C22; the positions of Ile154δ and Val48γ methyl signals are indicated with orange boxes in panel d).

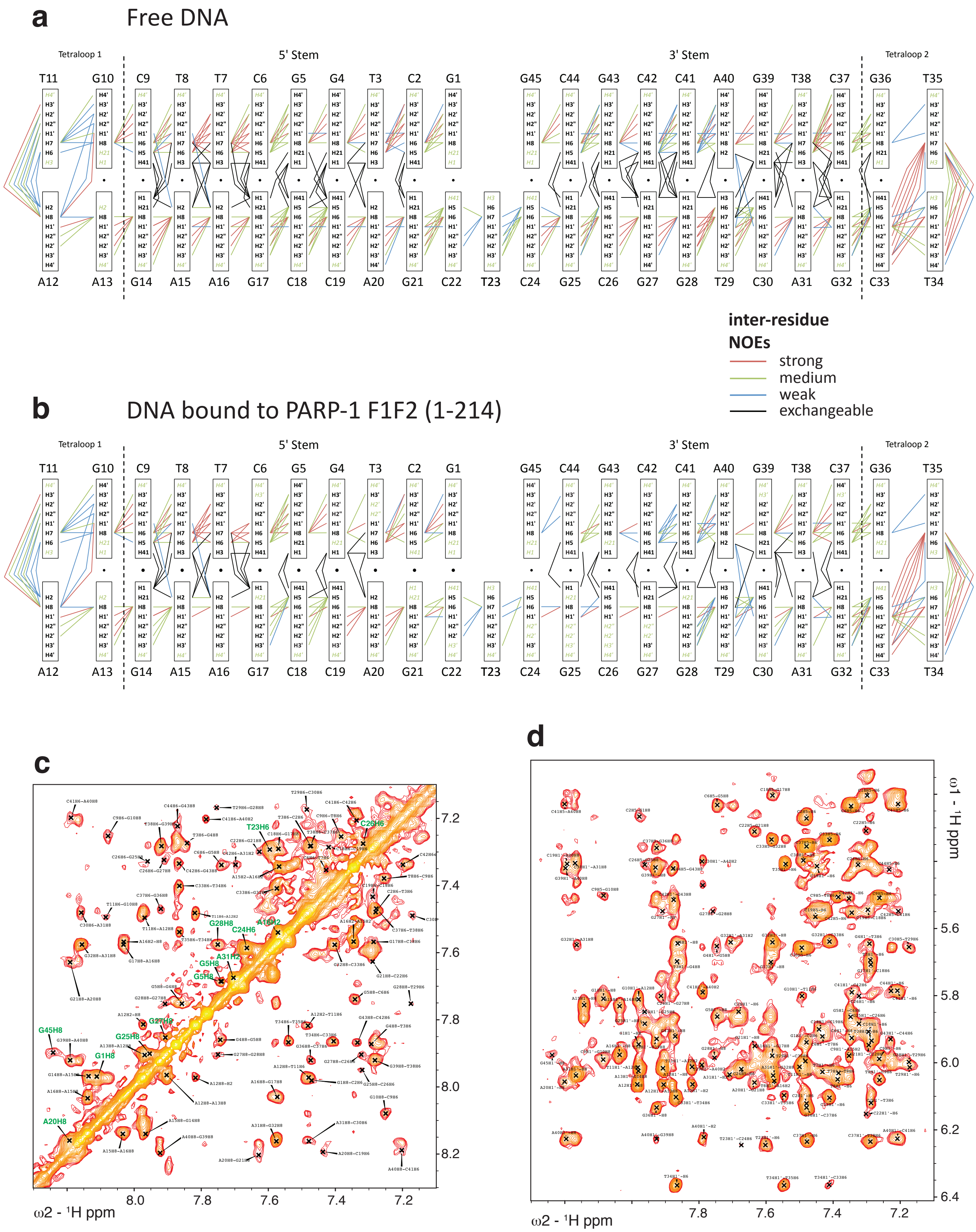
a
Free DNA
Tetraloop 1
5' Stem
3' Stem
Tetraloop 2
T11 G10 C9 T8 T7 C6 G5 G4 T3 C2 G1 G45 C44 G43 C42 C41 A40 G39 T38 C37 G36 T35
A12 A13 G14 A15 A16 G17 C18 C19 A20 G21 C22 T23 C24 G25 C26 G27 G28 T29 C30 A31 G32 C33 T34
inter-residue NOEs
strong
medium
weak
exchangeable
b
DNA bound to PARP-1 F1F2 (1-214)
Tetraloop 1
5' Stem
3' Stem
Tetraloop 2
T11 G10 C9 T8 T7 C6 G5 G4 T3 C2 G1 G45 C44 G43 C42 C41 A40 G39 T38 C37 G36 T35
A12 A13 G14 A15 A16 G17 C18 C19 A20 G21 C22 T23 C24 G25 C26 G27 G28 T29 C30 A31 G32 C33 T34
c
d
ω2 - ¹H ppm
ω1 - ¹H ppm

Figure S3, Related to Figure 1:

Comparison of free- and bound-state DNA signal assignments. Charts summarizing all the sequential NOE connectivities found during assignment of a) free and b) bound states of the 45nt gapped DNA dumbbell ligand. The similarity of the patterns (allowing for the reduced sensitivity and increased complexity of spectra of the complex) shows that the secondary structure of the dumbbell is maintained in the complex. Non-assigned protons are indicated in green italics (for simplicity, H5' and H5" signals are omitted from the schemes; in the free state, these were assigned for G1, G4, C6, T7, T8, G10 and T35, while for the bound state they were assigned only for T8). No attempt was made to quantitate crosspeaks involving exchangeable signals as the intensity of these varies greatly due to their different exchange rates.

Panels c) and d) show regions of a 2D [^{1}H,^{1}H] NOESY spectrum of the F1F2-DNA complex, showing data quality and extent of assignment. This sample contained a slight excess of DNA, which led to clear exchange crosspeaks linking corresponding free and bound signals (assignments shown in green on panel c); these were very useful in making assignments for those signals that shifted substantially upon complex formation.

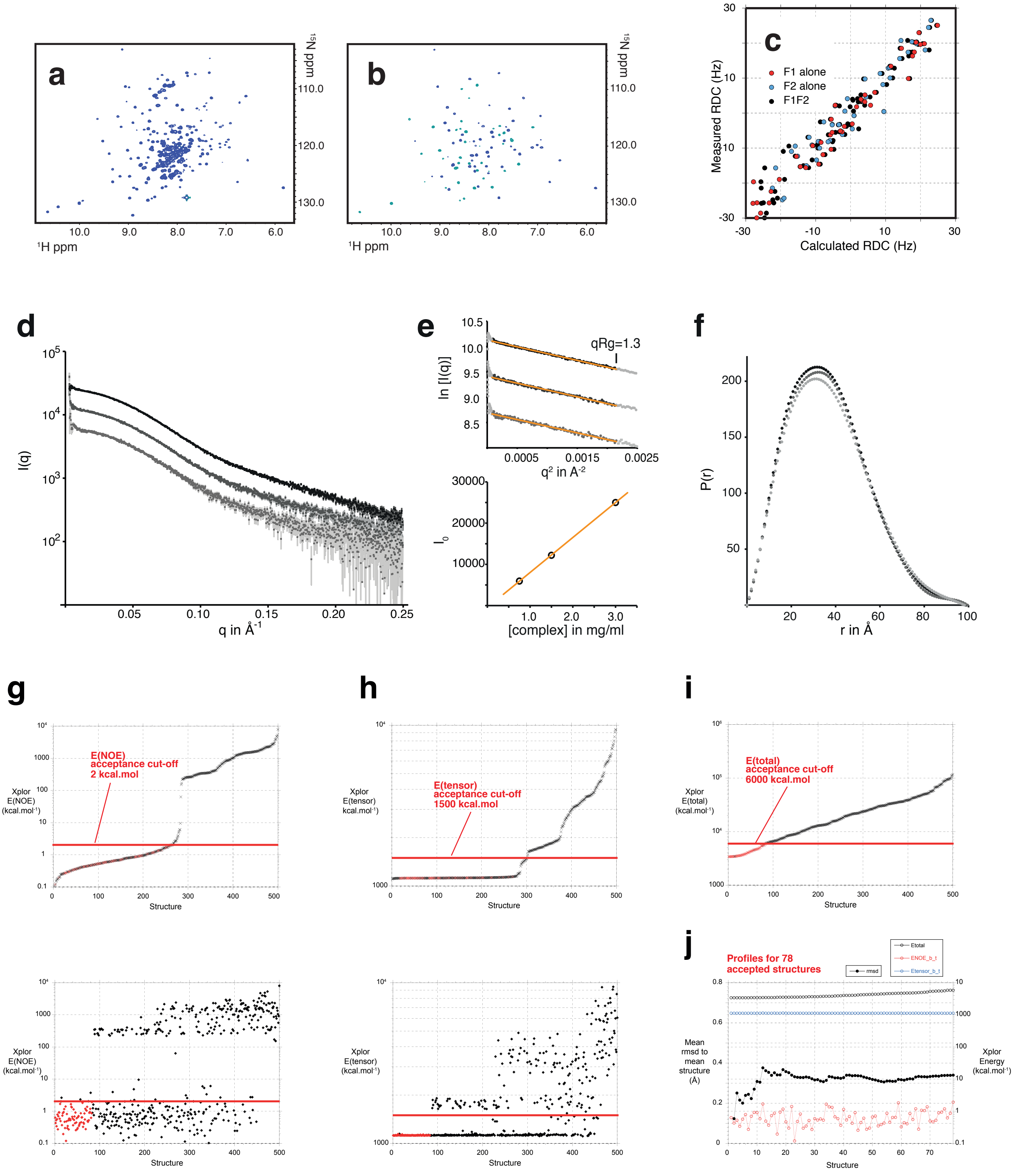

a
b
c
^{15}N ppm
110.0
120.0
130.0
10.0
9.0
8.0
7.0
6.0
^{1}H ppm
F1 alone
F2 alone
F1F2
Measured RDC (Hz)
Calculated RDC (Hz)
30
10
-10
-30
d
e
f
I(q)
q in Å^{-1}
0.05
0.10
0.15
0.20
0.25
qRg=1.3
ln [I(q)]
q^{2} in A^{-2}
0.0005
0.0015
0.0025
I$_{0}$
[complex] in mg/ml
1.0
2.0
3.0
P(r)
r in Å
20
40
60
80
100
g
h
i
E(NOE) acceptance cut-off 2 kcal.mol
Xplor E(NOE) (kcal.mol^{-1})
E(tensor) acceptance cut-off 1500 kcal.mol
Xplor E(tensor) (kcal.mol^{-1})
E(total) acceptance cut-off 6000 kcal.mol
Xplor E(total) (kcal.mol^{-1})
Structure
j
Profiles for 78 accepted structures
rmsd
Etotal
ENOE_b_t
Etensor_b_t
Mean rmsd to mean structure (Å)
Xplor Energy (kcal.mol^{-1})
Structure

Figure S4, Related to Figure 1:

a) and b) ARTSY spectra used to measure RDC values for the F1F2-DNA dumbbell complex. In the presence of pf1 phage, the sample becomes aligned and ^{15}N-^{1}H splittings become a composite of $^{1}J(^{15}N,^{1}H)$ and RDC values, which are measured indirectly using the intensity difference for a given peak between the “reference” (panel a) and “attenuated” (panel b) versions of the ARTSY spectrum (Fitzkee et al., 2010). c) Amide group RDC values measured for F1F2-DNA complex aligned by pf1 phage (see Supplemental Experimental Procedures) are plotted against corresponding values calculated using co-ordinates derived from crystal structures of F1-DNA complex (pdb 3ODA) and F2-DNA complex (pdb 3ODC); the RDC data used were restricted to residues shown to be rigid by the ^{15}N relaxation data and the conformations of which were invariant amongst different copies of the protein in the crystal structures. When F1 and F2 are treated separately (i.e. when each has its own independent alignment tensor), the Q values (18.9% for F1 and 29.9% for F2) show that in each case the structures were preserved to the degree expected for X-ray structures in this resolution range (2.6-2.8Å) (Bax et al., 2005), thereby demonstrating that the internal structures of F1 and F2 are similar in solution and in the crystals. When a single, combined tensor is used during the structure calculation protocol, this causes F1 and F2 to adopt a specific mutual orientation that optimises agreement with the RDC data. It is clear both from the plot and from the Q value (24.5%) that the fit obtained using a combined tensor is of comparable quality to that obtained using independent tensors, showing that the mutual orientation of the fingers in the calculated structure of the complex has converged to an optimal fit to the RDC data.

Panels d)-f) show measures of data quality for the Small Angle X-ray Scattering (SAXS) experiments. d) SAXS profiles of PARP-1 F1+F2 in complex with gapped 5'-phosphorylated DNA (DB4) at three different concentrations (3mg/ml black, 1.5mg/ml grey, 0.75 mg/ml light grey). Errors are indicated by light grey bars. e) Particle interference was shown to be absent using a Guinier analysis within the qRg<1.3 limit, as well as by demonstrating a direct proportionality between I_0 (derived from P(r)) and the sample concentrations used. f) Pair-distance distribution functions of the three data sets are largely consistent.

Panels g)-j) illustrate structure selection for the calculated structures of the F1F2-DNA dumbbell complex. Structures were included in the accepted ensemble if they simultaneously met the three criteria E(total) <= 6000 kcal.mol^{-1}, E(tensor) <=1500 kcal.mol^{-1} and E(NOE) <= 2 kcal.mol^{-1}; here E(total) represents the total XPLOR-NIH energy term (Schwieters et al., 2003), E(NOE) describes the quality of the fit to the NOE restraints and E(tensor) describes the quality of the fit to the RDC restraints. g)-i) Profiles showing the variation in energy terms g) E(NOE), h) E(tensor), and i) E(total) across all 500 calculated structures; accepted structures are shown in red, rejected in black, and the acceptance criteria for each energy term is shown with a horizontal red line. In g) the upper panel shows points ordered by increasing E(NOE) values, while the lower panel shows the same data points but reordered according to increasing E(total) values; similarly, in h) the upper panel shows points ordered by increasing E(tensor), the lower panel shows the same points re-ordered by increasing

E(total). These plots show how the acceptance criteria were chosen; for E(NOE) and E(tensor), it is clear that the accepted structures all lie within well-defined minimum plateau regions, but that these plateau regions also contain many structures with high E(total) values. E(total) varies over a wide range across the ensemble; this is not unexpected, since structures were calculated starting from conformers in which the 10 rotatable bonds of the DNA linker had been completely randomized and the ability of the calculation to converge on the lowest energy solution is strongly dependent on starting conformation. The acceptance criterion of E(total)<=6000 $kcal.mol^{-1}$ was chosen since this corresponds to roughly the highest value of E(total) for which structures with higher E(NOE) and E(tensor) values (outside the plateau regions) are largely excluded. However, as this single criterion was not sufficient to exclude a minority of structures with E(total)<=6000 $kcal.mol^{-1}$ but poor E(NOE) and/or E(tensor) values, we employed selection based on the three simultaneous criteria for E(total), E(NOE) and E(tensor) as described above. j) Profiles for the 78 accepted structures, showing values for E(total), E(NOE) and E(tensor); in all cases structures in this plot are arranged in order of increasing E(total). For the rmsd profile, values were independently calculated for each ensemble size using the program CLUSTERPOSE (Diamond, 1992, 1995), adding successive structures in order of increasing E(total).

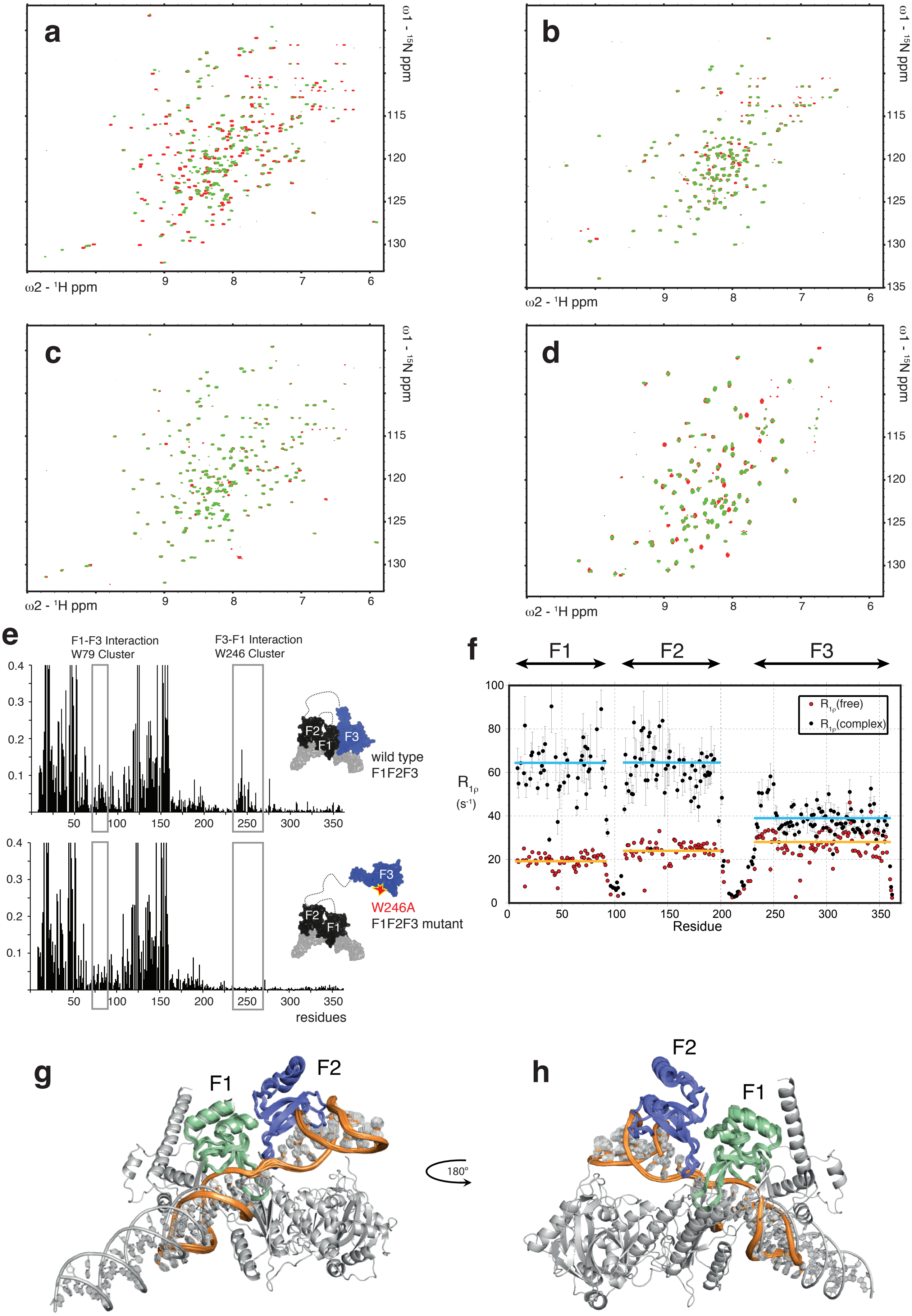
a
b
c
d
ω1 - ¹⁵N ppm
ω2 - ¹H ppm
9
8
7
6
115
120
125
130
135
e
F1-F3 Interaction
W79 Cluster
F3-F1 Interaction
W246 Cluster
0.4
0.3
0.2
0.1
50
100
150
200
250
300
350
F2
F1
F3
wild type
F1F2F3
W246A
F1F2F3 mutant
residues
f
F1
F2
F3
100
80
60
40
20
0
$R_{1\rho}$
(s^{-1})
$R_{1\rho}$(free)
$R_{1\rho}$(complex)
Residue
g
h
F1
F2
180°

Figure S5, Related to Figure 4:

Interactions of the F1F2-DNA dumbbell complex with the F3 and WGR domains of PARP-1. Panels a)–d) show the NMR data (all acquired at 800 MHz and 30°C) that were used to derive the chemical shift perturbations plotted in Figure 3. a) Overlay of TROSY spectra for the free (red) and DNA-bound (green) states of a sortase–ligated sample of F1F2F3 in which only F1F2 contributes signals (F1F2F3 labeling scheme II, see Supplemental Experimental Procedures). b) Overlay of TROSY spectra for the free (red) and DNA-bound (green) states of a sortase–ligated sample of F1F2F3 in which only F3 contributes signals. c) Overlay of TROSY spectra for the bound states of F1F2 (red) and F1F2F3 (green), signals from the latter being restricted to those from F1F2 by sortase ligation; these data were used to identify interaction surfaces between F3 and F1F2-DNA in the complex. d) Overlay of HSQC spectra for the free (red) and F1F2F3-DNA-bound (green) states of the WGR domain. e) CSP values observed upon DNA binding of the F1F2F3 W246A mutant (lower panel) plotted against corresponding CSP values for wild-type F1F2F3 (upper panel). The W246A mutation clearly abrogates the DNA-dependent interaction of F3 with F1 (CSPs in the W246 cluster are abolished in the mutant). The CSPs due to F3 interaction observed on F1 are also reduced in the mutant, though here interpretation is more difficult due to the large background of CSPs due to the DNA-F1 interaction. (For the wild-type protein, the data shown here were combined from measurements using the same two sortase ligated samples as used to construct the plots in Figure 3 of the main paper). f) ^{15}N $R_{1\rho}$ data ($1/T_{1\rho}$) for free and DNA-bound F1F2F3; average values are indicated for each domain in the free protein (orange) and the DNA-bound protein (cyan). The F1 and F2 domains show a strong enhancement of relaxation in the bound state, consistent with tumbling as a single entity in combination with the DNA, whereas the F3 signals show a more modest increase in relaxation rate, suggesting the interaction of F3 with the rest of the complex is relatively weak. The F1F2 and F2F3 linkers clearly remain flexible in the complex, as relaxation rates for linker residues are low.

g) and h) Superposition of the F1F2-DNA dumbbell structure with the crystal structure of F1, F3 and WGR-CAT bound to a DNA blunt end (pdb 4DQY); the structures were fitted using the backbone atoms of F1 (N, Cα and C' atoms of residues 2-7) and its associated DNA stem (C1', C2', C3', C4', C5', O3', O4' O5' and P of first 7 basepairs only). It is clear that this fit positions F2 and its associated DNA stem into a region of free space, without steric clashes.

Construct	Template used for (sub)cloning	Vector	Restriction sites used for cloning	Sequence boundaries	Expression/purification tag and protease site (N- and *C*-terminal)	Vector derived N- and *C*-terminal residues of purified construct
F1 (SL)	MGC clone IMAGE 5193735	pET28a	BamHI/NcoI	1-102	LPXTG sortase motif, His6	None<br>*LPETGGGRRHHHHHH*
F2 (SL)	MGC clone IMAGE 5193735	pET28a	BamHI/NcoI	105-214	His6 TEV site<br>*none*	GGG<br>*none*
F1F2	MGC clone IMAGE 5193735	pET13	BamHI/NcoI	1-214	none<br>*none*	none<br>*none*
F1F2 (SL)	codon-optimized PARP-1 gene (pQE7 plasmid; Qiagen)	pET28a-lip	NdeI/XhoI	1-214	none<br>*LPXTG sortase motif, His6*	none<br>*LPETGGGRRHHHHHH*
F3	MGC clone IMAGE 5193735	pGEX-6P1	BamHI/EcoRI	215-362	GST-domain<br>*none*	GPGS<br>*none*
F3 (SL)	codon-optimized PARP-1 gene (pQE7 plasmid; Qiagen)	pET28a-lip	NdeI/XhoI	215-362	His6, lipoyl domain, TEV<br>*none*	GG<br>*none*
F1F2F3	codon-optimized PARP-1 gene (pQE7 plasmid; Qiagen)	pET28a-lip	NdeI/XhoI	1-362	His6, lipoyl domain, TEV site<br>*none*	G<br>*none*
WGR	codon-optimized PARP-1 gene (pQE7 plasmid; Qiagen)	pET28a-lip	NdeI/XhoI	523-649	His6, lipoyl domain, TEV site<br>*none*	G<br>*none*
Full length PARP-1	(Langelier et al. 2008)	pET28a	NdeI/XhoI	1-1014	His6, Thrombin site<br>*none*	MGSSHHHHHHSSGLVPRGSH<br>*none*
Full length PARP-1	(Langelier et al. 2008)	pET28a-SMT	NdeI/XhoI	1-1014	His6, SUMO domain<br>*none*	His6, SUMO domain<br>*none*
GFP-PARP(F1)	(Langelier et al. 2011)	pEGFP-N1	NdeI/XhoI	1-234<br>Δ97-216	eGFP domain<br>*none*	Construct not purified; used for *in vivo* localization study
GFP-PARP(F2)	(Langelier et al. 2011)	pEGFP-N1	NdeI/XhoI	97-234	eGFP domain<br>*none*	Construct not purified; used for *in vivo* localization study
GFP-PARP(F1F2)	(Langelier et al. 2011)	pEGFP-N1	NdeI/XhoI	1-234	eGFP domain<br>*none*	Construct not purified; used for *in vivo* localization study
GFP-PARP(ΔF1F2)	(Langelier et al. 2011)	pEGFP-N1	NdeI/XhoI	201-1014	eGFP domain<br>*none*	Construct not purified; used for *in vivo* localization study
GFP-PARP(wt)	(Langelier et al. 2011)	pEGFP-N1	NdeI/XhoI	1-1014	eGFP domain<br>*none*	Construct not purified; used for *in vivo* localization study
wt Sortase Δ59 (Srt)	pGBMCS containing Srt Δ59 (gift Inagaki Lab)	pGBMCS (pet21b)	BamHI/XhoI	60-	GB1 domain<br>*His6*	GB1 domain<br>*HHHHHH*
Mutant Sortase Δ59 (SrtM) P94S, D160N, D165A, K196T	Synthesized gene (IDT) mutations according to (Liu et al. reference)	pGBMCS (pet21b)	BamHI/XhoI		GB1 domain<br>*His6*	GB1 domain<br>*HHHHHH*

Table S1, Related to Figure 1: PARP-1 protein constructs used in this work.

Intermolecular NOE	Expt./Sample		Interdomain NOE	Expt./Sample
Ile154 Hδ – G45 H1'	1, 2, 3, 4		Val60 Hγa – Met153 Hε	4, 5, 6, 7, 8, 9
Ile154 Hδ – G45 H2'	1, 2, 3		Val60 Hγb – Met153 Hε	4, 5, 6, 7, 8, 9
Ile154 Hδ – G45 H2"	1, 2, 3, 4		Ile37 Hδ – Val144 Hγa	8
Ile154 Hδ – G45 H3'	1, 2, 4		Ile37 Hδ – Val144 Hγb	8
Ile154 Hδ – G45 H4'	1, 2, 3, 4		Ile37 Hδ – Met153 Hε	5, 6, 7, 8
Ile154 Hδ – G45 H8	1, 2, 3, 4		Val39 Hγa – Pro149 Hβa	4
Leu151 Hδa - G45 H1'	1, 2, 3, 4		Val39 Hγa – Pro149 Hβb	4
Leu151 Hδa - G45 H2"	3		Val39 Hγb – Pro149 Hβa	4
Leu151 Hδb - G45 H1'	4		Val39 Hγb – Pro149 Hβb	4
Val48 Hγa – T23 M7	3, 4		Val39 Hγa – Pro149 Hγa/b	4
Val48 Hγb - T23 M7	3, 4		Val39 Hγb – Pro149 Hγa/b	4
Val48 Hγa – C22 H5	3, 4		Leu8 Hδa – Pro149 Hβa	4
Val48 Hγb – C22 H5	3, 4		Leu8 Hδa – Pro149 Hβb	4
Val48 Hγa/b – T23 H6	4		Leu8 Hδb – Pro149 Hβa	4
Val48 Hγa/b – C22 H1'	4		Leu8 Hδb – Pro149 Hβb	4
V48 Hγa – C22 H2'/H2"	3, 4			
V48 Hγb – C22 H2'/H2"	3, 4			

Table S2, Related to Figure 1: Intermolecular (left) and interdomain (right) NOE interactions observed for the F1F2-DNA dumbbell complex. For each of these assigned NOE interactions, the list of spectra in which the corresponding cross peaks were seen is not comprehensive, but lists cases where assignment was unambiguous. For definitions of isotope labelling schemes, see Supplemental Experimental Procedures.

Expt./Sample:

1) Experiment: 2D [^{1}H, ^{1}H] NOESY (τ_m = 200ms), filtered to accept only ^{13}C-bound ^{1}H signals in ω_2 (unfiltered in ω_1);
Sample: F1F2 isotope labelled according to scheme VII, in complex with 45nt gapped dumbbell DNA in ^2H_2O.

2) Experiment: 2D [^{1}H, ^{1}H] NOESY (τ_m = 200ms), filtered to accept only ^{13}C-bound ^{1}H signals in ω_2 (unfiltered in ω_1);
Sample: F1F2 isotope labelled according to scheme VII, in complex with 45nt gapped dumbbell DNA in ^2H_2O, no ^{13}C-decoupling applied in F_1 (only artefacts should differ between experiments 1 and 2).

3) Experiment: 3D [^{1}H, ^{13}C, ^{1}H] NOESY-HMQC (τ_m = 200ms);
Sample: F1F2 isotope labelled according to scheme IVa, in complex with 45nt gapped dumbbell DNA in H_2O.

4) Experiment: 2D [^{1}H, ^{1}H] NOESY (τ_m = 200ms), filtered to accept only ^{13}C-bound ^{1}H signals in ω_2 (unfiltered in ω_1);
Sample: F1F2 isotope labelled according to scheme IX, in complex with 45nt gapped dumbbell DNA in H_2O. Interdomain NOE crosspeaks listed for this experiment were all missing in a corresponding control spectrum acquired in the absence of DNA, showing that the interdomain interactions are DNA-dependent.

5) Experiment: 2D [^1H, ^1H] NOESY (τ_m = 200ms), filtered to accept only ^{13}C-bound ^1H signals in ω_2 (unfiltered in ω_1);
Sample: F1F2 isotope labelled according to scheme VIII, in complex with 45nt gapped dumbbell DNA in H_2O.

6) Experiment: 2D [^1H, ^1H] NOESY (τ_m = 200ms), filtered to accept only ^{13}C-bound ^1H signals in ω_2 (unfiltered in ω_1);
Sample: F1F2 isotope labelled according to scheme VIII, in complex with 45nt gapped dumbbell DNA in H_2O, no ^{13}C-decoupling applied in F_1 (only artefacts should differ between experiment 6 and 7).

7) Experiment: 2D [^1H, ^1H] NOESY (τ_m = 200ms), filtered to accept only ^{13}C-bound ^1H signals in ω_2 (unfiltered in ω_1);
Sample: F1F2 isotope labelled according to scheme V, in complex with 45nt gapped dumbbell DNA in ^2H_2O.

8) Experiment: 3D [^{13}C, ^{13}C, ^1H] HMQC-NOESY-HMQC (τ_m = 200ms);
Sample: F1F2 isotope labelled according to scheme V, in complex with 45nt gapped dumbbell DNA in ^2H_2O.

9) Experiment: 3D [^1H, ^{13}C, ^1H] NOESY-HMQC (τ_m = 200ms);
Sample: F1F2 isotope labelled according to scheme V, in complex with 45nt gapped dumbbell DNA in ^2H_2O.

Supplemental Experimental Procedures

Protein constructs:

Plasmids coding for human PARP-1 constructs were (sub)cloned from a human cDNA library, a MGC cDNA clone [IMAGE 5193735 (Geneservice,UK)] or from a PQE7 plasmid containing the human PARP-1 sequence codon-optimized for expression in *E. coli* (Qiagen); for details see Table S1. Full-length PARP-1 expression constructs were cloned in the pET28 vector as described (Langelier et al., 2008; Langelier et al., 2011), or into a pET28 vector modified to include the coding sequence for the SUMO polypeptide tag (SMT) in-frame at the N-terminus of PARP-1 (SMT-PARP-1). PARP-1 fusions with GFP (WT: 1-1014, ΔF1F2: 201-1014, F1F2: 1-234, F1:1-234 with 97-216 deleted, and F2: 97-234) were cloned in the pEGFP mammalian expression vector as described (Steffen et al., 2014). The plasmid for bacterial expression of wild-type Staphylococcus aureus Sortase Δ59 (SrtΔ59M) was a generous gift of Fuyuhiko Inagaki (Hokaido University, Japan). The vector (pGBMCS; http://www.addgene.org/21931/) was based on pET21b and contains a non-cleavable N-terminal GB1 fusion tag and a non-cleavable C-terminal His6 tag. The sortase gene for SrtΔ59M containing the mutations P94S, D160N, D165A, K196T (Chen et al., 2011) was synthesized by IDT (Integrated DNA Technologies), and cloned into pGBMCS using BamHI and XhoI restriction sites. Vectors (based on pET28a) containing DNA sequences encoding Bacillus stearothermophilus dihydrolipoamide acetyltransferase lipoyl domain (pet28a-lip) and Streptococcal protein GB1 (pET28a-GB1) as N-terminal expression tags with TEVpro cleavage sites were a generous gift from Peter Lukavsky (CEITEC, Masaryk University, Brno). Site-directed mutagenesis was achieved by PCR using QuikChange II methodology (Stratagene) using KOD Hot Start DNA Polymerase (Novagen).

Recombinant expression and isotope labeling of PARP-1 constructs:

Full-length PARP-1 WT and mutants, and SMT-PARP-1 WT and E988K mutant were expressed and purified using a published protocol (Langelier et al., 2010; Langelier et al., 2011) that uses Ni^{2+} and heparin affinity followed by size exclusion chromatography. Constructs of human PARP-1 derived from codon-optimized sequences were recombinantly expressed in *E.coli* BL21-DE3, while BL21-CodonPlus(DE3)-RP cells (Stratagene) were used for all other PARP-1 constructs. Unless otherwise stated all isotopically labeled compounds were obtained from from Sigma Isotec or Cambridge Isotope Laboratory.

Uniform ^{13}C and ^{15}N isotope labeling of protein constructs was essentially as described in Eustermann *et al.* (2011). PARP-1 fragments F1, F2 and F1F2 were “reverse” labeled using adapted protocols from (Marley et al., 2001; Takeuchi et al., 2007; Tugarinov et al., 2006). In each case a colony of freshly transformed cells was cultured in LB medium at 37°C to A_{600}=0.6, then cells were pelleted at 4°C, washed in M9-salt solution lacking all nitrogen and carbon sources, then pelleted as before and resuspended in M9 minimal medium supplemented with $^{15}NH_4Cl$ (0.5 g/L), $[^2H_7{}^{13}C_6]$-glucose

(8 g/L) and 98% ^2H_2O (all Sigma Aldrich Isotec). The volume of this culture was approx. one quarter that of the initial culture in LB, yielding A_{600} after resuspension of approximately 2.4. After incubation for 1h at 37°C, the medium was supplemented with sodium α-ketoisovalerate and sodium α-ketobutyrate (see schemes IVa and IVb in the NMR spectroscopy section for the labeling patterns used for these precursors) and/or [^{1}H,^{13}C,^{15}N] uniformly labeled amino acids (methionine [250 mg/liter], proline [156 mg/liter], arginine [120 mg/liter], lysine [120 mg/liter], tyrosine [120 mg/liter], phenylalanine [120 mg/liter] and/or ^{1}H,^{15}N uniformly labeled tryptophan [150 mg/liter]) (all compounds from Sigma Isotec or Cambridge Isotope Laboratories). After further incubation for 1h at 22°C, cultures were supplemented with 0.5 mM $ZnSO_4$ and protein expression induced by adding 0.5 mM IPTG, followed by incubation for 8-10h at 22°C, then cells were harvested and proteins purified as described. Metabolic scrambling during isotope labeling by residue type was minimized by using a protocol from Takeuchi *et al.* (2007) and supplementing, when required, the respective M9 medium with 8.5 g/l deuterated ^{13}C ^{15}N Celtone (Cambridge Isotope Laboratories).

Uniform ^{2}H, ^{13}C and ^{15}N labeling of F1, F2. F1F2, F1F2F3, F3 and WGR was achieved using similar protocols as described above for reverse labeling. Cells for expression of F1F2F3, F3 and WGR were grown in 2xTY medium until an A_{600} of 2 was reached, then exchanged into 98% ^2H_2O M9 medium supplemented with [$^2H_7{}^{13}C_6$]-glucose (4 g/L) and $^{15}NH_4Cl$ (0.5 g/L) as the sole carbon and nitrogen sources. After additional incubation for 45 min at 37°C followed by 45min at 22°C, expression was induced as described above.

These protocols yielded protein-deuteration levels of >95% (assayed by NMR), and selective ^{13}C labeling and protonation were sufficiently efficient for the NMR experiments described to succeed. Amide protons were essentially fully back-exchanged by overnight incubation in aqueous buffer. Segmental labeling was achieved by using Sortase-mediated protein ligation as described below.

Protein purification:

All purification steps were carried out at 4°C or on ice. Proteins carrying N- or C-terminal residues for sortase ligation (see below) are designated (SL). F1 (SL), F2 (SL) and F1F2 were purified as described in Eustermann *et al.* (2011). Harvested cells were resuspended in lysis buffer containing 50 mM Tris pH 7.4, 150 μM $ZnSO_4$, 4 mM DTT, 25% (w/v) sucrose and protease inhibitor mix (Roche Complete Protease Inhibitor Cocktail EDTA free; 1 tablet per 50 ml). After sonication, the cell lysate was cleared by centrifugation and filtered using 0.22 μM PVDF Stericup filter (Millipore). Initial protein purification employed ion-exchange chromatography using SP-Sepharose (GE-Healthcare), eluting with a linear NaCl gradient in 50 mM Tris pH 7.4, 150 μM $ZnSO_4$, 4 mM DTT and protease inhibitor mix (Roche Complete Protease Inhibitor Cocktail EDTA free; 1 tablet per 1 L). Eluted protein was exchanged to the same buffer without NaCl and purified further using a HiTrap Heparin HP column (GE-Healthcare), again eluting with a linear NaCl gradient. F2 (SL) was subjected to TEV cleavage and NiNTA affinity purification, as described below, to produce the N-terminal triple glycine motif required

for sortase ligation. Finally, all proteins were purified to homogeneity by size exclusion chromatography using a Superdex-S75 column (GE Healthcare) equilibrated with 50 mM Tris pH 7.4, 200 mM NaCl, 150 μM $ZnSO_4$, 4 mM DTT.

For purification of GST-tagged F3, cells were harvested after expression and resuspended in glutathione binding buffer (50 mM Tris pH 7.4, 1M NaCl, 150 μM $ZnSO_4$ and 1 mM DTT) containing EDTA free Complete Protease Inhibitor Cocktail (Roche) and lysed by sonication. The lysate was cleared by centrifugation and the supernatant incubated with Glutathione-Sepharose 4B (GE Healthcare), equilibrated in gluthathione binding buffer. Protein-bound beads were washed thoroughly with binding buffer and equilibrated into cleavage buffer (50 mM Tris pH 7.4, 150 mM NaCl, 100 μM $ZnSO_4$ and 1 mM DTT). The GST fusion protein was cleaved on the Glutathione-Sepharose beads by GST-tagged Precission Protease (GE Healthcare), leaving N-terminal vector-derived residues Gly-Pro-Gly-Ser N-terminal. Protein in supernatant was cleared off from gluthione sepharose 4B, equilibrated to gelfiltration buffer (50 mM Tris pH 7.4 , 250 mM NaCl, 100 μM $ZnSO_4$ and 1mM DTT) and concentrated using Vivaspin 20 MWCO 3000 (Sartorius-Stedim Biotech). Finally, F3 was purified to homogeneity by gelfiltration using Superdex S-75 equilibrated in the respective gelfiltration buffer.

For purification of F1F2F3, F1F2 (SL), F3 (SL) and WGR, harvested cells were resuspended in NiNTA binding buffer (50 mM Na-HEPES pH 7.5, 1 M NaCl, 4 mM β-mercaptoethanol, 20 mM imidazole, 5% glycerol) containing EDTA free Complete Protease Inhibitor Cocktail (Roche) as well as additional protease inhibitors (4 μg/ml aprotinin, 10 μg/ml leupeptin, 4 μg/ml pepstatin A, 4 μg/ml E64 and 1 mM PMSF). Cells were lysed by sonication, the lysate cleared off by centrifugation and the filtered supernatant loaded on a HisTrap HP Ni-affinity column (GE Healthcare). The His6-tagged proteins were eluted using a linear gradient of imidazole and dialysed overnight in 50 mM Na-HEPES pH 7.0, 0.2 M NaCl, 1 mM DTT, 5% glycerol. If required, the N-terminal fusion tag and His6 Tag were cleaved off by addition of His-tagged TEVpro during dialysis and the sample passed through a HisTrap HP Ni-affinity column to remove the protease and the cleaved tags. Following this, F1F2F3, F1F2 (SL), F3 (SL) were loaded on a HiTrap Heparin HP column (GE-Healthcare) and eluted with a linear NaCl gradient. Finally, all proteins were purified to homogeneity by size exclusion chromatography using a Superdex-S75 column (GE Healthcare) equilibrated with 50 mM Na-HEPES pH 7.5, 0.2 M NaCl, 2 mM DTT, 5% glycerol.

Sortase ligation:

Segmental labeling of PARP-1 constructs was achieved by sortase mediated protein ligation. *Staphylococcus aureus* Sortase Δ59 as well as a mutant variant Sortase Δ59 M (P94S, D160N, D165A, K196T), that has been evolved by yeast display to be catalytically more active (Chen et al., 2011), were purified as described previously (Kobashigawa et al., 2009). For each ligation, two protein starting materials were purified: one carrying a C-terminal LPXTG sortase recognition cleavage site as well as a His6 Tag, the other an N-terminal triple glycine motif, which has been previously shown to be well suited for efficient ligation(Huang et al., 2003) (see Table S1). The latter was produced by TEV

digestion of a N-terminal GENLYFQGGG motif. These constructs were mixed at a ratio of 1:1.25-2 at 100 μM in ligation buffer (50 mM Na-HEPES pH 7.5, 500 mM NaCl, 10 mM $CaCl_2$, 50 μM $ZnSO_4$, 2 mM DTT) and incubated in the presence of 2 μM Sortase Δ59 or Sortase Δ59 M for 20h at either 20C or 4C, respectively. The C-terminal cleavage by-product (GGGRRHHHHHH) was removed by dialysis against ligation buffer by using Slide-A-Lyzer cassettes with a MWCO of 3.500 or 10.000 (Pierce), as it would otherwise be a substrate for the back-reaction. The ligation mixture was then loaded onto a 5 ml HisTrap HP column (GE Healthcare) equilibrated in the ligation buffer without $CaCl_2$. The ligation product as well as the unligated C-terminal by-product were collected in the flow-through, while the other components were bound to the column and could be used for another round of ligation after elution with imidazol. Finally, the ligation product was purified to homogeneity by ion exchange chromatography using a MonoS 16/60 column.

Preparation of DNA dumbbell ligands:

5'-phosphorylated DNA dumbbell ligands were obtained from the in-house DNA synthesis facility of the MRC Laboratory of Molecular Biology or from Integrated DNA Technologies. The sequences of the DNA dumbbell ligands are:

DB1 5'-P-CGGTCGATCGTAAGATCGACCGTGCGCTGGAGCTTGCTCCAGCGC-3'

DB2 5'-P-CGGTCGATCGTAAGATCGACCGTCGCGGTCAGCTTGCTGACCGCG-3'

DB3 5'-P-GCTGGCTTCGTAAGAAGCCAGCTGCGCTGGAGCTTGCTCCAGCGC-3'

DB4 5'-P-GCTGGCTTCGTAAGAAGCCAGCTCGCGGTCAGCTTGCTGACCGCG-3'

Unless otherwise stated all experiments were performed with DB4. For AUC as well as fluorescence anisotropy experiments, a DNA dumbbell was synthesized that contained a fluoresceine derivatized thymidine at sequence position 8. DNA ligands have been further purified using denaturing polyacrylamide gel electrophoresis according to the protocol of Price *et al.* (1998). Correct folding of DNA ligands into a monomeric dumbbell conformation was verified using NMR as well as native PAGE.

Isothermal titration calorimetry:

ITC experiments employed a MicroCal iTC200 calorimeter (GE Healthcare). PARP-1 F1F2 solutions of 100-200 μM were titrated into 5-10 μM gapped DNA dumbbell ligand at 25° C. Typically, one initial injection of 0.5 μl was followed by 19 injections of 2 μl with 120 seconds between each step. Before each experiment, protein and DNA solutions were dialyzed overnight at 4 °C into ITC buffer (50 mM Tris pH 7.4, 50 μM $ZnSO_4$, 1 mM DTT and 200 mM NaCl) using Slide-A-Lyzer cassettes

(Pierce) with a MWCO of 3500Da. Protein and DNA concentrations were determined photometrically and adjusted by dilution. Excess heat upon each injection was integrated using an automatically adjusted base line and derived values corrected by heat of dilution. The latter was derived from the endpoints of protein-DNA titrations under saturating conditions. Data analysis and curve fitting was performed using the Origin 7 software package provided with the Microcal iTC200 calorimeter. The iTC200 was calibrated with EDTA-Ca^{2+} titrations prior to use according to the manufacturer's recommendations.

Fluorescence anisotropy:

Fluorescence polarization experiments were performed as described (Langelier et al., 2010) in 12 mM HEPES (pH 8.0), 60 mM KCl, 0.05 mg/ml BSA, 8 mM $MgCl_2$, and 4 % glycerol and using fluorescently labeled dumbbell DNA DB4.

Analytical ultracentrifugation:

Analytical ultra-centrifugation (AUC) experiments were performed and analyzed in SEDFIT(Schuck, 2005) as described(Langelier et al., 2012) in 25 mM Tris (pH 8.0), 150 mM NaCl, 1 mM EDTA, and 0.1 mM TCEP using full-length PARP-1 (4 μM) and fluorescently labeled dumbbell DNA DB4 (2 μM).

Activity assays:

The SDS-PAGE automodification assay was performed as described(Langelier et al., 2012) in 20 mM Tris (pH 7.5), 50 mM NaCl, 5 mM $MgCl_2$, and 0.1 mM TCEP using 1 μM protein, 5mM NAD^+ and 0.5 or 1 μM DNA depending on the template. When two proteins were combined in the same reaction 1 μM of each protein was used. Reactions containing plasmid DNA used the pUC19 vector modified to include a single nicking enzyme site (Nb.BsmI). The colorimetric assay of PARP-1 activity was performed as described (Langelier et al., 2010; Langelier et al., 2011) in 18 mM HEPES (pH 8.0), 150 mM NaCl, 0.5 mM TCEP, and 10 μg/ml BSA using 40 nM of dumbbell DNA DB4 and 20 nM PARP-1 WT or mutants, and 500 μM NAD^+.

Live cell imaging and laser irradiation:

HeLa cell transfection, sensitization and microirrradiation were performed essentially as described (Langelier et al., 2010; Steffen et al., 2014). Live cell images (512 x 512 pixels) were collected at 1 sec intervals on a Zeiss LSM-510 Meta Confocal laser scanning microscope by excitation with a 488 nm argon laser (8% power) focused through a 40x oil-immersion lens. The nuclei selected

for analysis had a starting GFP intensity ranging from 70 to 160 arbitrary units under this setup. A defined region of interest (ROI) in the nucleus was scanned with 5 iterations of a 405 nm diode laser (100% transmission) to create localized DNA damage. Images were processed and quantified using LSM Image (Zeiss). For quantification of fluorescence intensity changes, the ROI intensity was compared to the intensity of a non-irradiated region of the nucleus, providing a ratio of intensities (ROI/non-irradiated) that was averaged over ≥ 4 cells from a representative experiment.

Small-angle X-ray scattering:

SAXS data was acquired at 20°C at a wavelength of 1.24Å at the EMBL P12 beamline (Petra III, DESY, Hamburg) equipped with a photon counting Pilatus 2M pixel X-ray detector (Blanchet et al., 2015). F1F2-DNA complex was reconstituted as described for NMR spectroscopy using natural abundance protein and DNA (DB4 sequence). Prior to the SAXS measurement F1F2-DNA dumbbell complex was reconstituted as described for NMR spectroscopy and then subjected to another size exclusion chromatography step using a S75 column (GE Healthcare) equilibrated in 50 mM Tris pH 7.2, 200 mM NaCl, 1 mM DTT, 100 μM $ZnSO_4$ to yield monodisperse 1:1 complex. A series of concentrations was measured (adjusted to 3 mg/ml, 1.5 mg/ml and 0.75 mg/ml); the gelfiltration buffer also served as reference sample for the SAXS measurements. Data processing and analysis was performed by using the ATSAS program package: After averaging, data reduction and buffer subtraction, the program PRIMUS (Konarev et al., 2003) was used for evaluation of data quality by Guinier analysis (Fig. S4k). Radius of Gyration Rg as well as forward scattering I(0) were calculated using the entire scattering data using the program GNOM (Svergun, 1992), providing also pair distribution functions P(r) (Fig. S4m). Theoretical X-ray scattering profiles of individual NMR ensemble structures were calculated using the program Crysol (Svergun et al., 1995). The average profile shown in Fig. 1h is a linear combination of all 78 theoretical profiles calculated for the whole ensemble.

NMR spectroscopy:

NMR data were acquired using Bruker Avance I 800, Avance II+ 700, DMX600 and DRX500 spectrometers (Bruker BioSpin GmbH), each equipped with a cryogenically cooled triple resonance ($^{1}H/^{15}N/^{13}C$) 5mm probe. Solutions contained protein, DNA or protein-DNA complexes at 100-300 μM in either (A) 50 mM [$^{2}H_{11}$]-tris, 1 mM [$^{2}H_{10}$] DTT and 100 μM $ZnSO_4$ at pH7.2, $H_2O/^{2}H_2O$ 95:5 or $^{2}H_2O$ 98%, or (B) the same buffer with addition of 200 mM NaCl. Conditions (A) were used for NMR experiments involving F1F2-DNA complex or free DNA. Since constructs including the F3 domain were prone to precipitation at low ionic strength, conditions (B) were used for all experiments involving F1F2F3 free protein, F1F2F3-DNA complex, and for WGR and F1F2F3-DNA titrations with WGR; in addition, some assignment experiments for the F1F2-DNA complex were repeated under conditions (B) to allow chemical shift differences to the F1F2F3-DNA complex to be assessed under identical

conditions. When necessary, restricted volume NMR tubes were used to optimize sensitivity; for samples under conditions (A) these were Shigemi microtubes (Shigemi), while for samples under conditions (B) shaped NMR tubes (Bruker BioSpin GmbH) were used. Water suppression was achieved using the WATERGATE 3-9-19 pulse-sequence element (both for samples in H_2O and in ^2H_2O) (Piotto et al., 1992). Spectra were recorded at 25°C, 30°C or 37°C; ^1H chemical shifts were calibrated using sodium 3,3,3-trimethylsilylpropionate (TSP) as an external ^1H reference; ^{15}N and ^{13}C chemical shifts were indirectly referenced to the ^1H shifts using the ratio of gyromagnetic ratios. Spectra were processed using the program Topspin (Bruker BioSpin GmbH) and the NMRpipe package (Delaglio et al., 1995), while spectral analysis was performed in Sparky (Goddard) or the CCPN analysis package (Vranken et al., 2005).

Protein-DNA complexes were reconstituted using chemically synthesized DNA at natural abundance and recombinant protein isotopically labeled according to one of the following schemes:

F1F2 scheme I: Uniform [^2H,^{15}N,^{13}C];

F1F2 scheme II: Uniform [^2H (approx. 70%),^{15}N,^{13}C];

F1F2 scheme III: Uniform [^2H,^{15}N];

F1F2 scheme IVa: Uniform [^2H,^{15}N,^{13}C], back-labeled with [^1H,^{13}C] in the δ-methyl groups of Ile and all methyl groups of Leu and Val residues, using sodium [4-^{13}C, 3,3-$^2H_2$]α-ketobutyrate and sodium [3-^2H, 4,4'-$^{13}C_2$]α-ketoisovalerate as precursors to maximize protonation of methyl groups, for use in NOE experiments; sodium [3-^2H, 4,4'-$^{13}C_2$]α-ketoisovalerate was prepared from sodium [4,4'-$^{13}C_2$]α-ketoisovalerate by exchange with ^2H_2O at pH 12.5 and 45°C for 3 hrs (Goto et al., 1999);

F1F2 scheme IVb: Uniform [^2H,^{15}N,^{13}C], back-labeled with [^1H,^{13}C] in the δ-methyl groups of Ile and all methyl groups of Leu and Val residues, using sodium [3,3-$^2H_2$,$^{13}C_4$]α-ketobutyrate and sodium [3-^2H,$^{13}C_5$]α-ketoisovalerate as precursors to produce linear chains of ^{13}C in the sidechains of Val and Leu, for use in assignment experiments to link methyl signals to Cα signals;

F1F2 scheme V: Uniform [^2H,^{15}N,^{13}C], back-labeled with [^1H,^{13}C] in the methyl groups of Met residues in addition to Ile, Leu and Val methyl groups as in (IVa);

F1F2 scheme VI: Uniform [^2H,^{15}N,^{13}C]; back-labeled with [^1H,^{13}C] in the methyl groups of Ile, Leu and Val methyl groups as in (IVa) and [^1H,^{15}N,^{13}C] Phe residues;

F1F2 scheme VII: [^1H,^{15}N,^{12}C] F1 sortase ligated to [^2H,^{15}N,^{13}C] F2 back labeled with [^1H,^{13}C] Ile, Leu Val and Met methyl groups labeled as in (V);

F1F2 scheme VIII: [^1H,^{15}N,^{12}C] F1 sortase ligated to [^2H,^{15}N,^{13}C] F2 back labeled with [^1H,^{13}C] Met methyl groups as in (V) and [^1H,^{15}N,^{13}C] Arg residues;

F1F2 scheme IX: [^2H,^{15}N,^{13}C] F1 back-labeled with Ile, Leu and Val methyl groups labeled as in (IVa) and [^1H,^{15}N,^{13}C] Arg residues sortase ligated to [^1H,^{15}N,^{12}C] F2;

F1F2 scheme X: [^{2}H,^{15}N,^{13}C] F1 back-labeled with Ile, Leu and Val methyl groups labeled as in (IVa) and [^{1}H,^{15}N,^{13}C] Phe residues sortase ligated to [^{1}H,^{15}N,^{12}C] F2;

F1F2 scheme XI: [^{2}H,^{15}N,^{13}C] F1 back-labeled with Ile, Leu and Val methyl groups labeled as in (IVa) sortase ligated to [^{2}H,^{15}N,^{12}C] F2 back-labeled with [^{1}H,^{15}N,^{13}C] Pro;

F1F2F3 scheme I: Uniform [^{2}H,^{15}N,^{13}C];

F1F2F3 scheme II: F1F2 [^{2}H,^{15}N,^{13}C] sortase ligated to F3 at natural abundance;

F1F2F3 scheme III: F1F2 at natural abundance sortase ligated to F3 [^{2}H, ^{15}N, ^{13}C].

NMR assignment:

Essentially complete amide group assignments of PARP-1 constructs, both free and in complex with dumbbell DNA ligands, were obtained using the following strategy. First, full sets of assignments were obtained for the isolated domains; in the cases of F1 and F2, backbone and sidechain assignments were transferred from those reported in Eustermann et al., (2011), and in the cases of the isolated F3 and WGR domains, assignments were made using [^{15}N,^{13}C] labeled protein and a standard suite of NMR experiments ([^{15}N,^{1}H] HSQC, [^{13}C,^{1}H] HSQC, CBCANH, CBCA(CO)NH, HBHANH and HBHA(CO)NH). These assignments were then used as a starting point for making sequential assignments of the amide signals of multidomain PARP-1 fragments, in both their free and DNA-bound states. Assignments were made using [^{2}H,^{15}N,^{13}C] labeled protein in conjunction with TROSY-based experiments ([^{15}N,^{1}H] TROSY, [^{13}C-^{1}H] HMQC, TROSY-NHCACB (optimized for CB), TROSY-HNCA and TROSY-HN(CO)CA); chemical shifts of amide signals recorded using TROSY-based experiments were corrected by subtraction of 47Hz (=$^{1}J(^{15}N,^{1}H)/2$) in each dimension. Severe spectral crowding was overcome by using sortase-mediated protein-protein ligation as described above to prepare samples in which the observed signals were limited to those from particular domains; thus, in different samples, NMR signals were restricted to the F1, F2, F1F2 or F3 domains and their respective linker regions, while in each case the other parts of the same polypeptide chain were kept NMR silent (this corresponds to labeling schemes F1F2 VII-XI and F1F2F3 II and III as listed above). Segmental labeling of F1F2F3 was similarly used to relieve overlap and facilitate protein backbone assignments for ternary F1F2F3-WGR-DNA complexes; different samples were made that comprised F1F2F3 labeled according to schemes I, II or III in complex with DNA and WGR domain, or alternatively [^{2}H,^{15}N,^{13}C] labeled WGR domain in complex with DNA and (natural abundance) F1F2F3. Using this strategy, amide signals in the various fragments and complexes were assigned to the following extents: F1F2-DNA, 200/204, 98.0%%; F1F2F3, 331/346, 95.7%%; F1F2F3-DNA, 330/346, 95.4%; F1F2F3-WGR-DNA, 324/346, 93.6%; F1F2F3(W246A), 327/346 94.5%; F1F2F3(W246A)-DNA, 325/346. 93.9%; F1F2F3(W246A)-DNA-WGR, 320/346, 92.5%.

All δ-methyl groups of Ile, all Leu and Val methyl groups (with the sole exception of the methyls of Val202) as well as all Met methyl groups of DNA-bound F1F2 were assigned (see Figure S2). The

majority of the methyls of Ile, Val and Leu were assigned using HMCM(CG)CBCA experiments (Tugarinov and Kay, 2003), to link to the corresponding Cα signals (that had themselves been previously assigned using the backbone experiments), recorded using DNA-bound F1F2 labeled according to scheme IVb and in $^{2}H_2O$ buffer. These methyl assignments were confirmed and extended using NOE-based data, particularly 3D [^{1}H,^{13}C,^{1}H] NOESY-HMQC and 3D [^{13}C,^{13}C,^{1}H] HMQC-NOESY-HMQC experiments (τ_c=200ms) recorded using DNA-bound F1F2 labeled according to scheme IVa and in $^{2}H_2O$ buffer; these experiments, together with 2D [^{1}H,^{1}H] NOESY and ^{15}N NOESY-HSQC experiments recorded in H_2O buffer, established an extensive network of NOE connectivities, all of which were consistent with the known structures of the DNA-unbound proteins. A further check on the assignments was provided by segmental labeling of F1 and F2 (F1F2 schemes VII-XI), which established unambiguously from which finger any given signal originated. Assignments for the methionine methyl signals were made using NOE connectivities to Ile, Val and Leu methyl signals (in experiments recorded in $^{2}H_2O$) and to backbone amide signals (in experiments recorded in H_2O), again using sortase ligated samples to distinguish from which finger given signals originated. In addition, assignments were obtained for the aromatic signals of phenylalanine residues in F1 and for sidechain signals of Pro residues in F2. The Phe44 aromatic signals were identified using unambiguous NOEs to a methyl of Val48 and to backbone amide signals of Met43 and Asp45 in experiments using DNA-bound F1F2 labeled according to scheme X and in $^{2}H_2O$ buffer. Sidechain signals of prolines in F2 were assigned starting from Cα assignments previously established in backbone experiments, using samples of DNA-bound F1F2 labeled according to scheme XI together with 2D [^{13}C,^{1}H] HSQC experiments recorded in $^{2}H_2O$ to extend these to the Hα signals and then using 2D [^{13}C,^{1}H] HSQC-NOESY (τ_c=100ms) recorded on the same samples to further extend the assignments onto corresponding Hβ and Hγ signals (Figure S2); this process did not allow a full assignment of all proline sidechain protons, but unambiguous assignments were obtained for most Hβ and Hγ signals, including those of Pro149 (Figure S2). Samples containing back-labeled Arg, Lys, Trp and Tyr residues were also made in an effort to measure further intermolecular protein-DNA and inter-domain NOEs (see below). However, any signals from these residues in the NOE-based experiments were below the detection threshold, presumably because they have more complicated multiplet structures and suffer faster relaxation than do the methyl signals; consequently, we did not pursue their assignment, except for sidechain NH signals of Trp.

DNA assignment:

Non-exchangeable signals of the 45 nucleotide free DNA ligand (sequence DB4) were assigned following standard protocols for B-form DNA (Wüthrich, 1986) using 2D (^{1}H,^{1}H) NOESY, TOCSY and DQF-COSY spectra recorded in $^{2}H_2O$ (recorded at 800 MHz and 37°C, τ_m=200ms), and these assignments were extended to base-paired imino- and amino- signals using cross-peaks in NOESY spectra recorded in H_2O. A complete assignment was obtained for the H1', H2', H2" and H3' and aromatic protons (excepting only A13 H2), while for the H4' protons, and particularly for the H5' and

H5" protons, signal overlap limited the extent of assignment; imino and H-bonded amino proton assignments were obtained for all of the A:T and G:C basepairs except for the G:C pairs on either side of the gap. Signals in the two tetraloops were assigned unambiguously using connectivity patterns observed in the ^2H_2O spectra; in addition, those of the CTTG loop were very similar to assignments previously published for a small circular DNA dumbbell containing a CTTG loop (Ippel et al., 1992, 1995). DNA signals in the complex were assigned using patterns of connectivities in 2D (^1H,^1H) NOESY spectra filtered to remove ^{13}C-bound signals in $\omega 2$, where possible making sequential walks characteristic for B-form DNA, and comparing these with corresponding patterns and assignments in the free DNA. During this process, a number of factors were helpful in overcoming the severe crowding in the 2D NOESY spectra of the complex; comparison of spectra at different temperatures and in either H_2O or ^2H_2O helped in resolving many ambiguities due to overlap, while signals arising from the tetraloops and adjacent basepairs, away from the protein-binding region, occurred at very similar chemical shifts to those for the free DNA. A particularly powerful approach for signals that changed shift substantially upon protein-binding, and would thus otherwise have been the most difficult to assign, was to use exchange cross-peaks observed in 2D NOESY spectra from samples containing a slight excess of DNA (protein:DNA ratio, 0.95:1). Under the conditions used, the kinetics of the complex were such that exchange cross-peaks linking corresponding DNA signals in the free and bound states were of comparable intensity to strong NOE cross-peaks (see Figure S3); for this to occur k_{off} must presumably be comparable to or faster than $1/T_1$ (Combrisson et al., 1971; Neuhaus and Williamson, 2000). Assignments made using such cross-peaks could additionally be confirmed by the further presence of exchange-relayed NOE cross-peaks in some cases, as well as by comparison with spectra from samples lacking excess DNA where cross-peaks caused by free/bound exchange were missing. The extent of the DNA assignment free and bound to PARP-1 F1F2 is summarized in Figure S3.

Chemical Shift Perturbation analysis:

Using the assignments described above, chemical shift perturbations upon protein-DNA interactions were calculated according to the formula $((\Delta\delta(^1H))^2 + (\Delta\delta(^{15}N))^2/5)^{1/2}$ for protein amide groups, $((\Delta\delta(^1H))^2 + (\Delta\delta(^{13}C))^2/10)^{1/2}$ for protein methyl groups and $((\Delta\delta(H1'))^2 + (\Delta\delta(H^{arom}))^2)^{1/2}$ for DNA chemical shift perturbations (where H^{arom} is H8 for Ade, H6 for Cyt, H8 for Gua and H6 for Thy).

Residual Dipolar Couplings:

RDCs were measured for DNA-bound [^2H,^{15}N] labeled PARP-1 F1F2 protein using the ARTSY pulse sequence (Fitzkee and Bax, 2010) at 600 MHz, recorded in an interleaved manner with ^1H dephasing durations of 5.375 ms and 10.75 ms. ^1JNH were extracted from measured ARTSY peak intensity ratios (Fitzkee and Bax, 2010) of an isotropic sample adjusted to buffer condition A, while

^{1}JNH+^{1}DNH values were determined for a weakly aligned sample made by addition of filamentous phage Pf1 (ASLA Biotech) to a final concentration of approximately 8mg/ml. The RDC data used in the structure calculations were restricted to values for residues shown to be rigid by the ^{15}N relaxation data and for which the conformations were invariant amongst different copies of the protein in the crystal structures. For the RDC analysis of the crystal structures of F1 and F2 we used the ISAC protocol (Sass et al., 2001) as implemented in XPLOR-NIH (Schwieters et al., 2003). In order to judge the quality of correlation between the RDCs back-calculated for respective structures and the experimentally determined values, we calculated Q values ($Q=RMS(D_i^{obs}-D_i^{calc})/RMS(D_i^{obs})$) as described by Bax and Grishaev (2005).

Relaxation analysis:

For the DNA-bound F1F2 complex (labeling scheme V), per-residue effective correlation times ($\tau_{c(effective)}$) at 37°C were determined at 800 MHz by using ^{15}N TRACT experiments (Lee et al., 2006) that measure the rate difference between the sharp and broad components of TROSY multiplets. Experiments were carried out in an interleaved manner with relaxation periods of 2, 2, 4, 8, 14, 14, 20, 22, 32, 48, 66, 96, 96, 140 and 240ms. Relaxation rates were determined using peak analysis scripts within the NMRpipe software package (Delaglio et al., 1995). Associated errors were estimated using data from the duplicated delays, and per-residue effective correlation times were then calculated according to (Lee et al., 2006). For F1F2F3 complexes, the broad TROSY component decayed too quickly for TRACT experiments to be feasible; instead ^{15}N T_1, $T_{1\rho}$ and steady-state {^{1}H}^{15}N NOE data were acquired at 800 MHz and 30°C, using the optimized pulse sequences of Lakomek et al. (2012). The relaxation delays used were as follows: T_1 (free protein): 0, 40, 40, 100, 180, 320, 320, 500, 800, 1200ms; T_1 (complex): 0, 60, 60, 140, 240, 400, 700, 1200, 2000ms; $T_{1\rho}$ (free protein): 0.2, 5, 5, 9, 17, 25, 40, 40, 70, 100ms; $T_{1\rho}$ (complex): 0.2, 1, 3, 3, 6, 10, 16, 25, 40ms; steady-state {^{1}H}^{15}N NOE saturation/relaxation period, 6.6s. $T_{1\rho}$ values were corrected for resonance offset effects (spin-lock tilt-angle) as described by Lakomek et al. (2012). Curve-fitting of these data was carried out using routines within the CCPN analysis software package (Vranken et al., 2005), and errors were estimated using data from the duplicated delays.

NOE-based restraints:

NOE restraints provided key atom-specific information required to characterize the architecture of the F1F2-DNA complex. Initial attempts to measure intermolecular NOE interactions between uniformly [^{1}H,^{13}C,^{15}N] labeled protein and natural abundance DNA by using conventional filtered NOE experiments all failed; not unexpectedly, given the size of the system, rapid transverse relaxation rendered the signals undetectable. To overcome this we turned to reverse labeling schemes in which various combinations of selected amino acid types (Phe, Tyr, Trp, Lys, Arg, Pro and Met) as

well as methyl groups of Ile, Val and Leu were protonated and ^{13}C-labeled in an otherwise deuterated protein background (see isotope labeling schemes F1F2 I–XI described above), thereby reducing simultaneously spectral complexity and transverse relaxation. Although it necessarily reduced the number available, this approach made it possible to detect NOE interactions at high resolution using 2D [^{1}H-^{1}H] NOESY experiments having only a single isotope half-filter, set to accept only ^{13}C-bound ^{1}H signals in ω2, and thereby restricting DNA signals to appear only in ω1. Spectral complexity was further reduced by segmental labeling of F1 and F2 (see below), and assignment of intermolecular NOE interactions was further aided by using 3D [^{1}H,^{13}C,^{1}H] NOESY-HMQC and 3D [^{13}C,^{13}C,^{1}H] HMQC-NOESY-HMQC experiments. Intriguingly, exchange-relayed intermolecular NOE crosspeaks were also observed in samples that contained an excess of DNA (see DNA assignment); once correctly identified, these were useful in confirming assignments. Identification of artifacts in the 2D spectra was aided by recording data separately with and without ^{13}C decoupling in ω1; unwanted intramolecular protein crosspeaks due to breakthrough gain a splitting in ω1 in the undecoupled dataset, whereas genuine intermolecular cross-peaks remain un-split. Intermolecular NOE crosspeaks that were detected using this strategy were largely restricted to interactions involving methyl groups; attempts to measure intermolecular NOE interactions involving sidechain $^{13}C^{1}H_2$ or $^{15}N^{1}H$ groups of Arg or Lys residues, that could have helped characterize protein interactions with the DNA phosphate backbone, were all unsuccessful, as were attempts to detect NOE interactions of the aromatic rings of [$^{1}H^{13}C$]-labeled Phe residues intended to detect intermolecular interactions of Phe44. The assigned intermolecular NOE crosspeaks and the experiments and samples that were used to detect them are summarized in Table S2.

A similar strategy was employed for identification of the DNA-dependent interdomain NOE interactions linking F1 and F2. In order to isolate these from others, samples of the F1F2-DNA complex were made that contained sortase-ligated protein in which one domain was reverse-labeled and deuterated while the other was [^{1}H,^{12}C,^{15}N] labeled, according to one of the schemes VII-XI. Such samples allowed detection of interdomain NOE crosspeaks at high resolution when used in similar experiments to those described for detecting the intermolecular NOE interactions. 2D [^{1}H-^{1}H] NOESY experiments were recorded with and without heteronuclear decoupling in ω1, as well as with a single half-filter set to accept only ^{13}C-bound ^{1}H signals in ω2, so as to restrict signals from ^{12}C attached protons (from the [^{1}H,^{12}C,^{15}N] labeled finger and the DNA) to the ω1 dimension. As an additional control, some spectra were recorded for the protein in the free state, in order to show that the observed interdomain contacts were only present in the DNA-bound state of the protein. Unambiguous assignment of interdomain NOE interactions between methyl groups was further aided by using 3D [^{1}H,^{13}C,^{1}H] NOESY-HMQC and 3D [^{13}C,^{13}C,^{1}H] HMQC-NOESY-HMQC experiments recorded using DNA-bound F1F2 labeled according to scheme IVa and in $^{2}H_2O$ buffer. The assigned interdomain NOE crosspeaks and the experiments and samples that were used to detect them are summarized in Table S2.

In order to use these assigned NOE interactions as restraints in the structure calculations, their associated upper distance bounds were uniformly set to 6.0Å. A true, experiment-based calibration of these NOE intensities would be extremely difficult to envisage, as the nature of the various interacting pairs varies considerably; in most cases the interaction spans between a deuterated and a non-deuterated domain or the DNA, involves a methyl group sometimes at one site and sometimes at both, and in addition many of the protein signal intensities are likely to be strongly modulated by differing label incorporation efficiencies at the different sites. Setting the upper bounds to 6Å results in negligible violations in the best fitting structures, and reducing the upper-bound distances uniformly to 5Å has a negligible impact on the calculated co-ordinates and the violations (data not shown), from which we conclude that use of 6Å upper bounds represents a conservative approach. Lower bounds were uniformly set to zero, and r^{-6} averaging was employed for equivalent and non-stereoassigned groups. Methyl signals of Leu and Val residues were not stereoassigned..

Structure calculations; i) template structures:

The starting point for calculations were the deposited co-ordinates of PARP-1 F1 and F2 domains in complex with DNA double-strand breaks; for the F1 complex the co-ordinates of chains B, I and J from pdb 3ODA were used, and for the F2 complex those of chains B, E and F from pdb 3ODC. The program XPLOR-NIH was used for all calculations. Hydrogen atoms were first added according to standard geometries, and the structures then subjected to 3000 cycles of Powell minimization while rigidly fixing the co-ordinates of the N, C' and O atoms of every peptide bond in the protein as well as all atoms of the DNA. This allowed the protein structures largely to equilibrate with XPLOR-NIH force field while exactly preserving the relative orientations of the individual amide bond vectors, as well as the relationship of the protein to the DNA. Only very small movements of the protein backbone occurred during this minimization; for F1 (residues 6-91) the backbone co-ordinate shift (rmsd for N, Cα, C') was 0.081Å, while for F2 (residues 109-200) the corresponding shift was 0.077Å. We refer to the resulting structures as F1-DNA$_{min}$ and F2-DNA$_{min}$ respectively.

To model the DNA dumbbell, initially a simulated annealing protocol was used to calculate an ensemble of 50 structures from torsion-angle randomized starting conformations, restraining the stems to reproduce ideal B-form geometry and the tetraloops, in the absence of corresponding deposited DNA structures, to reproduce the RNA tetraloop structures 1MSY (for G10-A13) and 1RNG (for C33-G36; the lowest energy structure of 1RNG was used). Experimental NOE connectivities measured for the DNA dumbbell in both free and bound states (summarized in Figure S3) are largely consistent with these RNA tetraloop structures and suggest they are an adequate approximation to the true solution conformations. One difference concerns the base of T35, which is clearly in the anti conformation in the DNA case. This is not unexpected, as the syn conformation of the corresponding uridine in 1RNG is maintained by an H-bond to the 2'OH group of the preceding ribose, which clearly cannot form in the DNA case; consequently the χ angle restraint used for T35 in the calculations was

altered from -155° to +25°. Overall, the restraints used comprised 478 dihedral angle restraints (set to ranges of ±5°; of these, 383 were in the stems and 95 in the tetraloops), 55 H-bonding distance restraints (49 in the stems set to ranges of ±0.1Å, and 6 in the tetraloops set to ±0.2Å), 18 weak basepair planarity restraints (force constant 50 $kcal.mol^{-1}.Å^{-2}$, except for the stem-closing basepairs G1:C22 and C24:G45 where the force constant was 200 $kcal.mol^{-1}.Å^{-2}$) for basepairs in the stems as well as for G10:A13 and C33:G36 in the tetraloops, and in the stems additional 1-3 phosphorus-phosphorus lower limit constraints (distance > 10.5Å) to aid convergence (Cerdan et al., 2001).

The lowest energy structure from this ensemble was then subjected to further annealing using a set of constraints designed to reproduce almost exactly, in the context of the dumbbell, the backbone conformation of the DNA in the complexes 3ODA and 3ODC within the region of the protein footprints. We refer here to the stem of the dumbbell which binds to F1 and contains the 5' terminus as stem 1, and that which binds F2 and contains the 3' terminus as stem 2. The restraints applied to stem 1 were based on target values measured for chains I and J in 3ODA, and comprised 193 dihedral angles in nucleotides 1-9 and 14-22, in addition to 48 long-range intra- and inter-strand distances in nucleotides 1-7 and 16-22 (these spanned between 2 and 7 basepairs, and comprised 24 intra-strand O3'-O5', 12 inter-strand O3'-O3' and 12 inter-strand O5'-O5' distances), while those applied to stem 2 were based on target values measured for chains E and F in 3ODC, and comprised 168 dihedral angles in nucleotides 24-31 and 38-45, in addition to 48 long-range intra- and inter-strand distances in nucleotides 24-30 and 45-39; dihedral angle restraints were applied with a range of ±5° and force constant increasing during the protocol to reach 100 $kcal.mol^{-1}$, and the long-range distance restraints were exact (±0Å), applied with a force constant of 50 $kcal.mol^{-1}$. Dihedral angle restraints in the tetraloops as well as basepair H-bond distance restraints throughout were applied as before (GC H-bond restraints: G(N1)-C(N3), 2.95 ± 0.1 Å; G(N2)-C(O2), 2.86 ± 0.1 Å; G(O6) – C(N4), 2.91 ± 0.1 Å. AT H-bond restraints: A(N1)-T(N3), 2.82 ± 0.1 Å; A(N6)-T(O4), 2.95 ± 0.1 Å; all force constants 50 $kcal.mol^{-1}$). Basepair planarity restraints were applied very weakly (force constant 20 $kcal.mol^{-1}.Å^{-2}$ in the stems, and 10 $kcal.mol^{-1}.Å^{-2}$ for basepairs G10:A13 and C33:G36 in the tetraloops). We refer to the resulting structure as DNA-$dumbbell_{anneal}$.

Structure calculations; ii) ensemble calculations:

To calculate an ensemble of models for the F1F2-DNA complex, 500 different random starting conformations were first created by randomizing the ten rotatable bonds in the continuous strand of the DNA linker between C22 O3' and C24 O5' within DNA-$dumbbell_{anneal}$. Next, the common DNA backbone atoms in the 7-basepair region of each protein domain's DNA-binding footprint were used to fit F1-DNA_{min} and F2-DNA_{min} onto the corresponding DNA backbone atoms of the stems of DNA-$dumbbell_{anneal}$, thereby carrying each protein domain accurately into the same spatial relationship to the appropriate stem of the dumbbell as it had with blunt-ended DNA in complex 3ODA (for F1) or 3ODC (for F2); the fits obtained are shown below:

Stem 1		
3ODA, chain I	DNA-dumbbell$_{anneal}$ stem 1	rmsd
nucleotide 1:<br>C1', C2', C3', C4', C5', O3', O4' and P<br>*nucleotides 2-7:*<br>C1', C2', C3', C4', C5', O3', O4', O5', P	*nucleotide 1:*<br>C1', C2', C3', C4', C5', O3', O4' and P<br>*nucleotides 2-7:*<br>C1', C2', C3', C4', C5', O3', O4', O5', P	0.234Å
3ODA, chain J	DNA dumbbell$_{anneal}$ stem 1	
nucleotide 4:<br>C1', C2', C3', C4', C5', O3', O4'<br>*nucleotides 5-10:*<br>C1', C2', C3', C4', C5', O3', O4', O5', P	*nucleotide 16:*<br>C1', C2', C3', C4', C5', O3', O4'<br>*nucleotides 17-22:*<br>C1', C2', C3', C4', C5', O3', O4', O5', P	
Stem 2		
3ODA, chain F	DNA-dumbbell$_{anneal}$ stem 2	rmsd
nucleotide 1:<br>C1', C2', C3', C4', C5', O3', O4' and P<br>*nucleotides 2-7:*<br>C1', C2', C3', C4', C5', O3', O4', O5', P	*nucleotide 24:*<br>C1', C2', C3', C4', C5', O3', O4' and P<br>*nucleotides 25-30:*<br>C1', C2', C3', C4', C5', O3', O4', O5', P	0.202Å
3ODA, chain E	DNA dumbbell$_{anneal}$ stem 2	
nucleotide 2:<br>C1', C2', C3', C4', C5', O3', O4'<br>*nucleotides 3-8:*<br>C1', C2', C3', C4', C5', O3', O4', O5', P	*nucleotide 39:*<br>C1', C2', C3', C4', C5', O3', O4'<br>*nucleotides 40-45:*<br>C1', C2', C3', C4', C5', O3', O4', O5', P	

Once these fits had been carried out, the original DNA atoms derived from 3ODA and 3ODC were deleted, leaving only the F1 and F2 domains in their bound conformations on the dumbbell. The structures were then subjected to 50 cycles of Powell minimization while holding the positions of the peptide bond atoms (N, C', O and HN) rigidly fixed to preserve relative amide bond orientations.

These starting structures of the dumbbell with bound F1 and F2 domains were then subjected to a simulated annealing protocol to generate an ensemble of conformers consistent with the RDC, intermolecular and interdomain NOE restraints. The protocol comprised 4000 cycles of Powell

minimization, followed by 40,000 steps of Langevin dynamics at 500K with reduced timestep of 0.0001ps, then 40,000 steps of cooling to 300K with timestep 0.001ps, and 5000 steps of final Powell minimization. For calculating the RDC energy terms a single alignment tensor was used for the whole complex, and the tensor parameters were allowed to float for best fit during the calculation, as previously described (Sass et al., 2001). The force constant used for the NOE restraints was 50 kcal.mol^{-1}. During these calculations strong non-crystallographic symmetry (NCS) constraints were used to maintain the internal structure of the DNA stems, the F1 and F2 domains, and the relationship of each finger to the stem to which it is bound. To achieve this the entire structure was duplicated, the co-ordinates of one copy rigidly fixed and groups of NCS constraints defined between the fixed and unfixed copies so as to maintain these elements of the structure in the evolving co-ordinates of the unfixed copy. Once preliminary rounds of calculation had established likely regions where contacts between F1 and F2 might occur, the NCS terms for just these regions were restricted to backbone atoms and were applied with a much reduced force constant; the NCS constraints used in the final rounds of calculations are shown below:

NCS constraint group	Atoms constrained	Force constant (kcal.mol^{-1})
F1/stem1	All atoms of protein residues 9-36, 49-59 and 66-89;<br>All atoms of DNA nucleotides 1-22.	10,000
	Backbone heavy atoms (N, Cα, C') of protein residues 6-8, 37-48, 60-65 and 90-91;<br>All carbon atoms of protein residue 44.	50
F2/stem2	All atoms of protein residues 114-140 and 155-199;<br>All atoms of DNA nucleotides 25-45.<br>All atoms except O5', P, O1P and O2P of DNA nucleotide 24.	10,000
	Backbone heavy atoms (N, Cα, C') of residues 109-113, 141-154 and 200-201.	50

Structures were included in the accepted ensemble if they met simultaneously all of the following criteria: E(total) <= 6000 kcal.mol^{-1}, E(tensor) <=1500 kcal.mol^{-1}, and E(NOE) <= 2 kcal.mol^{-1}.

Finally, the remaining atoms of the protein N- and C-terminal tails (residues 1-5 and 202-214) and the F1-F2 linker (residues 92-108) were added in a separate simulated annealing protocol. Initially

all atoms of the full structure (residues 1-214 of the protein and 1-45 of the DNA, including the 5' terminal phosphate group) were placed at fully randomized positions within a 200Å cube, then for those residues included in the previous stages of the calculations (i.e. residues 6-91 and 109-201 of the protein and nucleotides 1-45 of the DNA, not including the 5' terminal phosphate group) the randomized co-ordinates were replaced by the previously calculated values and rigidly fixed in place. The structures were then subjected to 200 cycles of Powell minimization, followed by 20,000 steps of Langevin dynamics at 500K with timestep 0.001ps and reduced Van der Waals radii and force constant, followed by 152,000 steps during which the Van der Waals radii and force constant were gradually increased and 102,000 steps during which the temperature was lowered to 300K, and a final 1000 steps of Powell minimization. This protocol for adding the tails and linker was repeated independently 5 times for each input structure using a different randomization seed each time, and the structure with the lowest value of E(total) retained.

As a control, an ensemble of 100 calculations was run in which the DNA was omitted and only the RDC constraints applied, to verify that the under these circumstances the protocol resulted in a statistical distribution of the four possible mutual orientations of F1 and F2 consistent with the RDC data. Analysis of the results (data not shown) demonstrated that there was essentially complete convergence to the four possible relative orientations, the four groups having populations of 23, 25, 25 and 27.

Parallel calculations were also run for alternative models:

1) Ensembles in which the template structure for F1 and its associated DNA stem were extracted from pdb 4DQY (chain A, with DNA backbones of chains M 1-10 and N 13-22). This was done in order to refine the fitting of the F3, WGR and CAT domains from 4DQY to the hybrid model; because the duplex DNA conformations in 3ODA and 4DQY differ somewhat, the quality of the fit is degraded when the template used for calculation of the hybrid structure is derived from 3ODA (Figure S5g,h). Alternatively, if 4DQY is itself used as the source of the template structures for F1 and its associated DNA stem, then a very precise fit is obtained, allowing more reliable modeling of the relationship between the domains. The lowest energy structure from an ensemble calculated in this way was used to prepare Figures 2, 3 and 4 of the main paper.

2) Ensembles in which the linker was shortened by deletion of residues 94-102 as described by (Ali et al., 2012), showing that the shorter linker is easily able to connect the F1 and F2 domains in the model (Figure S4j).

Structure analysis:

Structural superpositions optimized for simultaneous best fits across each ensemble were calculated using the program CLUSTERPOSE (Diamond, 1992, 1995). F1 fits refer to protein residues 6-91, F2 fits to protein residues 109-201, DNA stem 1 fits to nucleotides 1-22 and DNA stem 2 fits to residues 24-45 (excluding C24 O5' and P). For backbone fits the N, Cα and C' atoms of protein residues and the C1', C2', C3', C4', C5', O3', O4', O5' and P atoms of DNA were used. Ramachandran statistics for the protein were calculated using the program Procheck-NMR (Laskowski et al., 1996); these statistics demonstrated that the backbone geometry of the crystal structures 3ODA and 3ODC was maintained through the calculation protocol. DNA bend angles were calculated using the program 3DNA (Lu and Olson, 2003), using co-ordinates of basepairs 1:22-5-18 to define the 5' stem and 24:45-28-41 to define the 3' stem, and interface areas were calculated using the program PISA (Krissinel and Henrick, 2007). Structures were visualized using the program PyMol (DeLano, 2002).

Supplementary References

Blanchet, C.E., Spilotros, A., Schwemmer, F., Graewert, M.A., Kikhney, A., Jeffries, C.M., Franke, D., Mark, D., Zengerle, R., Cipriani, F., *et al.* (2015). Versatile sample environments and automation for biological solution X-ray scattering experiments at the P12 beamline (PETRA III, DESY). Journal of applied crystallography *48*, 431-443.

Cerdan, R., Payet, D., Yang, J.C., Travers, A.A., and Neuhaus, D. (2001). HMG-D complexed to a bulge DNA: an NMR model. Protein Sci *10*, 504-518.

Chen, I., Dorr, B.M., and Liu, D.R. (2011). A general strategy for the evolution of bond-forming enzymes using yeast display. Proc Natl Acad Sci U S A *108*, 11399-11404.

Combrisson, S., Roques, B., Rigny, P., and Basselier, J.J. (1971). Description de l'effet Overhauser dans les systèmes en échange: Application à l'analyse conformationnelle d'aldéhydes hétérocycliques. Canadian Journal of Chemistry *49*, 904-913.

Delaglio, F., Grzesiek, S., Vuister, G.W., Zhu, G., Pfeifer, J., and Bax, A. (1995). NMRPipe: a multidimensional spectral processing system based on UNIX pipes. J Biomol NMR *6*, 277-293.

DeLano, W.L. (2002). The PyMOL Molecular Graphics System (Palo Alto, CA: DeLano Scientific).

Diamond, R. (1992). On the multiple simultaneous superposition of molecular structures by rigid body transformation. *Protein Sci 1*, 1279-1287.

Diamond, R. (1995). Coordinate-based cluster analysis. Acta Cryst D *51*, 127-135.

Fitzkee, N.C., and Bax, A. (2010). Facile measurement of (1)H-(1)5N residual dipolar couplings in larger perdeuterated proteins. J Biomol NMR *48*, 65-70.

Goddard, T.D., Kneller, D. G. SPARKY 3 (San Francisco: University of California).

Goto, N.K., Gardner, K.H., Mueller, G.A., Willis, R.C., and Kay, L.E. (1999). A robust and cost-effective method for the production of Val, Leu, Ile (delta 1) methyl-protonated 15N-, 13C-, 2H-labeled proteins. J Biomol NMR *13*, 369-374.

Huang, X., Aulabaugh, A., Ding, W., Kapoor, B., Alksne, L., Tabei, K., and Ellestad, G. (2003). Kinetic mechanism of Staphylococcus aureus sortase SrtA. Biochem *42*, 11307-11315.

Ippel, J.H., Lanzotti, V., Galeone, A., Mayol, L., van den Boogaart, J.E., Pikkemaat, J.A., and Altona, C. (1992). An NMR study of the conformation and thermodynamics of the circular dumbbell d [formula: see text] Slow exchange between two- and four-membered hairpin loops. Journal of Biomolecular Structure & Dynamics *9*, 821-836.

Ippel, J.H., Lanzotti, V., Galeone, A., Mayol, L., Van den Boogaart, J.E., Pikkemaat, J.A., and Altona, C. (1995). Slow conformational exchange in DNA minihairpin loops: a conformational study of the circular dumbbell d<pCGC-TT-GCG-TT>. Biopolymers *36*, 681-694.

Konarev, P.V., Volkov, V.V., Sokolova, A.V., Koch, M.H.J., and Svergun, D.I. (2003). PRIMUS: a Windows PC-based system for small-angle scattering data analysis. Journal of applied crystallography *36*, 1277-1282.

Krissinel, E., and Henrick, K. (2007). Inference of macromolecular assemblies from crystalline state. J Mol Biol *372*, 774-797.

Lakomek, N.A., Ying, J., and Bax, A. (2012). Measurement of (1)(5)N relaxation rates in perdeuterated proteins by TROSY-based methods. J Biomol NMR *53*, 209-221.

Langelier, M.F., Planck, J.L., Servent, K.M., and Pascal, J.M. (2011). Purification of human PARP-1 and PARP-1 domains from Escherichia coli for structural and biochemical analysis. Methods in molecular biology *780*, 209-226.

Langelier, M.F., Ruhl, D.D., Planck, J.L., Kraus, W.L., and Pascal, J.M. (2010). The Zn3 domain of human poly(ADP-ribose) polymerase-1 (PARP-1) functions in both DNA-dependent poly(ADP-ribose) synthesis activity and chromatin compaction. J Biol Chem *285*, 18877-18887.

Langelier, M.F., Servent, K.M., Rogers, E.E., and Pascal, J.M. (2008). A third zinc-binding domain of human poly(ADP-ribose) polymerase-1 coordinates DNA-dependent enzyme activation. J Biol Chem *283*, 4105-4114.

Laskowski, R.A., Rullmannn, J.A., MacArthur, M.W., Kaptein, R., and Thornton, J.M. (1996). AQUA and PROCHECK-NMR: programs for checking the quality of protein structures solved by NMR. J Biomol NMR *8*, 477-486.

Lee, D., Hilty, C., Wider, G., and Wuthrich, K. (2006). Effective rotational correlation times of proteins from NMR relaxation interference. J Magn Reson *178*, 72-76.

Lu, X.J., and Olson, W.K. (2003). 3DNA: a software package for the analysis, rebuilding and visualization of three-dimensional nucleic acid structures. Nucleic Acids Res *31*, 5108-5121.

Marley, J., Lu, M., and Bracken, C. (2001). A method for efficient isotopic labeling of recombinant proteins. J Biomol NMR *20*, 71-75.

Neuhaus, D., and Williamson, M.P. (2000). The nuclear Overhauser effect in structural and conformational analysis; Second edition (New York: Wiley-VCH), pp. 136-143.

Piotto, M., Saudek, V., and Sklenar, V. (1992). Gradient-tailored excitation for single-quantum NMR spectroscopy of aqueous solutions. J Biomol NMR *2*, 661-665.

Price, S., Oubridge, C., Varani, G., and Nagai, K. (1998). Preparation of RNA:protein complexes for X-ray crystallography and NMR. In RNA:protein interactions : a practical approach, C.W.J. Smith, ed. (Oxford ; New York: Oxford University Press), pp. 37-72.

Schuck, P. (2005). In “Modern Analytical Ultracentrifuge: Techniques and Methods”, D.J. Scott, S.E. Harding, and A.J. Rowe, eds. (Cambridge: The Royal Society of Chemistry), pp. 26-60.

Schwieters, C.D., Kuszewski, J.J., Tjandra, N., and Clore, G.M. (2003). The Xplor-NIH NMR molecular structure determination package. J Magn Reson *160*, 65-73.

Steffen, J.D., Tholey, R.M., Langelier, M.F., Planck, J.L., Schiewer, M.J., Lal, S., Bildzukewicz, N.A., Yeo, C.J., Knudsen, K.E., Brody, J.R., *et al.* (2014). Targeting PARP-1 allosteric regulation offers therapeutic potential against cancer. Cancer Res *74*, 31-37.

Svergun, D. (1992). Determination of the regularization parameter in indirect-transform methods using perceptual criteria. Journal of applied crystallography *25*, 495-503.

Svergun, D., Barberato, C., and Koch, M.H.J. (1995). CRYSOL - A program to evaluate x-ray solution scattering of biological macromolecules from atomic coordinates. Journal of applied crystallography *28*, 768-773.

Takeuchi, K., Ng, E., Malia, T.J., and Wagner, G. (2007). 1-C-13 amino acid selective labeling in a (HN)-H-2-N-15 background for NMR studies of large proteins. J Biomol NMR *38*, 89-98.

Tugarinov, V., Kanelis, V., and Kay, L.E. (2006). Isotope labeling strategies for the study of high-molecular-weight proteins by solution NMR spectroscopy. Nature protocols *1*, 749-754.

Tugarinov, V., and Kay, L.E. (2003). Ile, Leu, and Val methyl assignments of the 723-residue malate synthase G using a new labeling strategy and novel NMR methods. J Am Chem Soc *125*, 13868-13878.

Vranken, W.F., Boucher, W., Stevens, T.J., Fogh, R.H., Pajon, A., Llinas, M., Ulrich, E.L., Markley, J.L., Ionides, J., and Laue, E.D. (2005). The CCPN data model for NMR spectroscopy: development of a software pipeline. Proteins *59*, 687-696.

Wüthrich, K. (1986). NMR of Proteins and Nucleic Acids (New York: John Wiley & Sons).
